# Use and detailed metric properties of patient-reported outcome measures for rheumatoid arthritis: a systematic review covering two decades

**DOI:** 10.1136/rmdopen-2021-001707

**Published:** 2021-08-10

**Authors:** Ayşe A Küçükdeveci, Atilla H Elhan, Beyza D Erdoğan, Şehim Kutlay, Derya Gökmen, Can Ateş, Selcen Yüksel, Asa Lundgren-Nilsson, Reuben Escorpizo, Gerold Stucki, Alan Tennant, Philip G Conaghan

**Affiliations:** 1 Department of Physical Medicine and Rehabilitation, Ankara University, Faculty of Medicine, Ankara, Turkey; 2 Department of Biostatistics, Ankara University, Faculty of Medicine, Ankara, Turkey; 3 Department of Biostatistics, Aksaray University, School of Medicine, Aksaray, Turkey; 4 Department of Biostatistics, Ankara Yıldırım Beyazıt University, Faculty of Medicine, Ankara, Turkey; 5 Institute of Neuroscience and Physiology, Sahlgrenska Academy, University of Gothenburg, Goteborg, Sweden; 6 Department of Rehabilitation and Movement Science, University of Vermont, Burlington, Vermont, USA; 7 Department of Health Sciences and Health Policy, University of Lucerne, Luzern, Switzerland; 8 Leeds Institute of Rheumatic and Musculoskeletal Medicine, University of Leeds and NIHR Leeds Biomedical Research Centre, Leeds, UK

**Keywords:** arthritis, rheumatoid, patient reported outcome measures, qualitative research

## Abstract

**Introduction:**

The importance of patient-reported outcome measures (PROMs) for rheumatoid arthritis (RA) clinical studies has been recognised for many years. The current study aims to describe the RA PROMs used over the past 20 years, and their performance metrics, to underpin appropriate tool selection.

**Methods:**

The study included a systematic search for PROMs that have been in use over the period 2000–2019, with detailed documentation of their psychometric properties, and a user-friendly presentation of the extensive evidence base.

**Results:**

125 PROMs were identified with psychometric evidence available. The domains of pain, fatigue, emotional functions, mobility, physical functioning and work dominated, with self-efficacy and coping as personal factors. Domains such as stiffness and sleep were poorly served. The most frequently used PROMs included the Health Assessment Questionnaire Disability Index (HAQ), the Short Form 36 (SF-36), the EuroQoL and the Modified HAQ which, between them, appeared in more than 3500 papers. Strong psychometric evidence was found for the HAQ, and the SF-36 Physical Functioning and Vitality (fatigue) domains. Otherwise, all domains except stiffness, sleep, education and health utility, had at least one PROM with moderate level of psychometric evidence.

**Conclusion:**

There is a broad range of PROMs for measuring RA outcomes, but the quality of psychometric evidence varies widely. This work identifies gaps in key RA domains according to the biopsychosocial model.

**Key messages**

**What is already known about this subject?**

- A wide range of patient-reported outcome measures (PROMs) are commonly used for rheumatoid arthritis (RA) outcomes.
- Some PROMs, for example, Health Assessment Questionnaire Disability Index, are well known.

**What does this study add?**

- This is the first comprehensive review of all RA PROMs in published studies, including trials.
- This work provides a detailed psychometric analysis of RA PROMs, and highlights deficits in measurement of several domains.

**How might this impact on clinical practice or further developments?**

- Existing PROMs encompass a wide range of domains, and some lesser-known outcomes that may be useful for clinicians.
- This work enables appropriate selection of PROMs, based on their performance for assessing particular domains.

## Introduction

**Figure 1:**
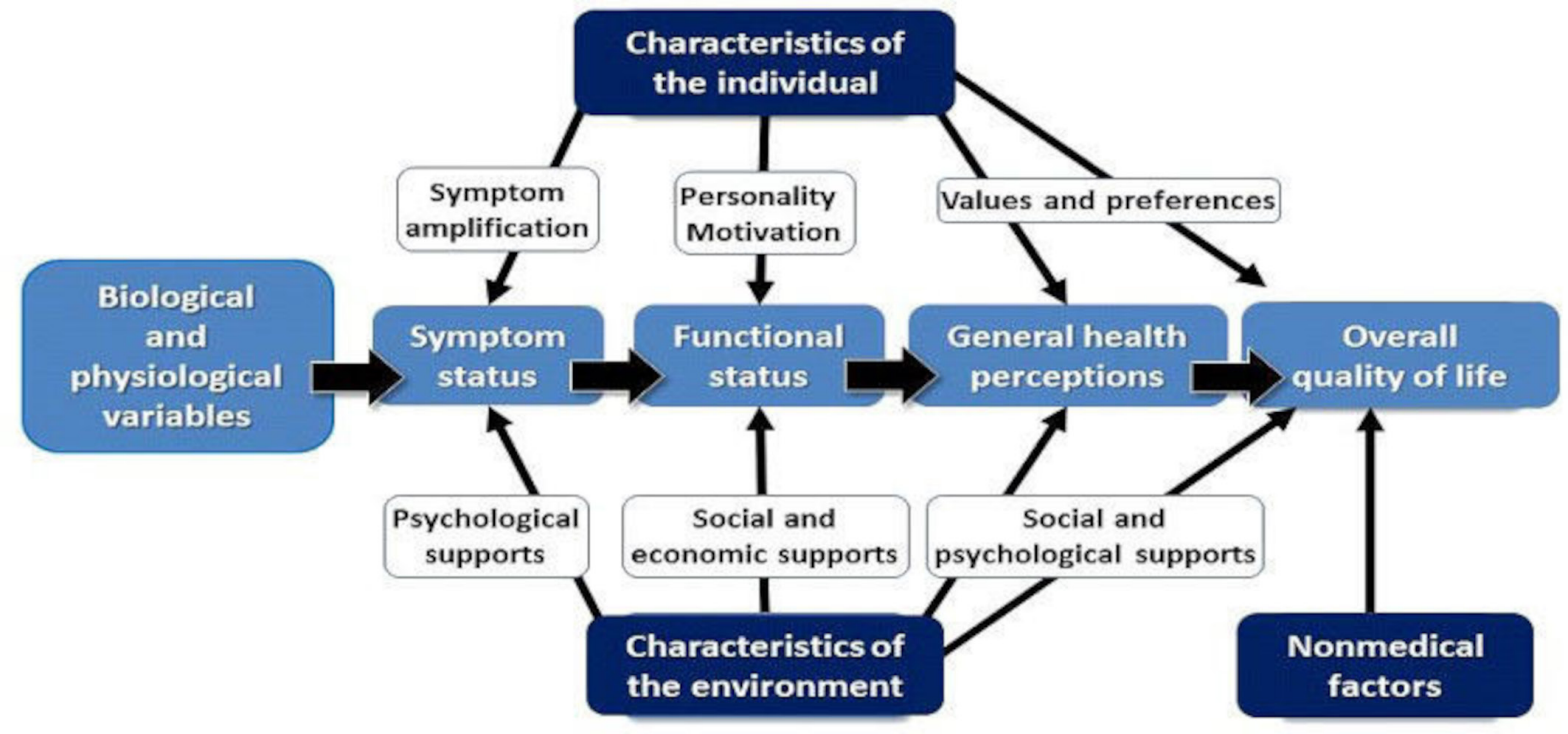
The biopsychosocial model of Wilson and Cleary.

The impact of rheumatoid arthritis (RA) on the health status of the individual has long been understood.^1–3^ With an increased understanding of the conceptual basis of outcomes in general, the concentration on physical aspects associated with early studies has expanded to include psychological and mental health consequences, and also to examine contextual factors which may influence the impact of the condition.^4–6^ As a result, an increasing biopsychosocial perspective is often to be found.^7^ Thus, for routine clinical monitoring, clinical trials, clinical epidemiology and other types of health services research, a range of outcomes will be used. For this work, an ‘outcome’ is defined as any indicator (variable) which can be used to either describe, or detect change in health status, psychological aspects or quality of life. Many outcomes used to monitor the progression of RA over the past 40 years have consisted of patient-reported outcome measures (PROMs) involving self-completed questionnaires.^1 8^ These would focus on symptoms such as pain and fatigue, as well as disability and quality of life. Most aspects can be catalogued through the International Classification of Functioning, Disability and Health (ICF) which has been recommended as one option for health recording in eHealth Informatics,^9^ for example, pain (b280: sensation of pain), mobility (d4) or work (eg, d8451—maintaining a job). Quality of life is a separate domain consistent with the Wilson and Cleary model.^4^ Together with the environmental and personal factors, they constitute the biopsychosocial model which defines the individual’s lived experience of RA (figure 1).^7 10–12^

Various systematic reviews have been published to help select the most appropriate PROM with the best evidence, however, these are frequently associated with a single domain or specific joints.^13–16^ Given the substantial number of domains that could be considered, and their associated PROMs, a catalogue of available PROMs across all relevant domains should be of value. Consequently, the current EULAR funded study, aimed to review all available PROMs used in RA, together with their performance metrics to help make informed choices about the most appropriate PROM for a given purpose.

## Methods

The study set out to identify the PROMs that have been in use over a 20-year period (2000–2019), and to systematically catalogue their psychometric properties. This is a sister study of the earlier paper on PROMs used in osteoarthritis, using the same methodology which is described later.^17^

### Definition of PROM

In this study we define PROM as any patient-(or proxy-) completed questionnaire where a set of items are summated to give a total score, or a series of subscale scores, or both.

### Systematic search

#### Search strategy

Electronic searches were performed in databases indexing health-related journals using Medline via PubMed and Scopus. Three different searches were used; the first to identify PROMs in use during the specified period (2000–2019); the second to identify papers for a specific PROM where some form of psychometric evidence was present; the third to count the number of times a PROM was used during the search period. An example of search 1, as PubMed search criteria, is presented in online supplemental file 1. The second search simply adds the name of the PROM using ‘AND’ as Boolean operator to the first part of the search, but without giving a specified period, as the psychometric evidence could arise from any period following the construction of the PROM. This was to identify the relevant psychometric evidence associated with the PROM. The third search removes the psychometric parameters to simply count the use of the PROM in RA in PubMed during the period 2000–2019. Targeted hand searching of reference lists and other supplementary sources, such as textbooks, was also performed.

10.1136/rmdopen-2021-001707.supp1 Supplementary data

#### Process of selection and data extraction

Potential papers with a candidate PROM identified in search 1 were then screened by two independent reviewers (AAK, SK). This included independent screening of the titles and abstracts. For search 2, having added the name of the PROM to the search criteria without date restriction, papers were included if they met the following criteria: (1) the subjects related to the evidence had RA and the evidence was RA specific; (2) one or more of the chosen psychometric criteria specific to the PROM (or its subscales) in question (eg, reliability) were reported in the article; (3) the article was in English and (4) it was available in full text. These selected papers were again reviewed by two independent reviewers and any disagreements were discussed and resolved with a third reviewer (AT).

### Reporting

The results are reported in a series of hierarchically structured tables and spreadsheets: (i) overall summary table—main paper; (ii) PROM-specific summaries—online supplemental file 2; (iii) references for the PROMs—online supplemental file 3; (iv) references used for evidence—online supplemental file 4 and (v) those scales excluded due to lack of evidence—online supplemental file 5. The summaries are catalogued according to domains such as pain or quality of life, with associated ICF classification following, where relevant, in parentheses. Those PROMs with subscales will have evidence presented at the subscale level and for total scores where relevant and, as such, some PROMs will appear more than once under subscale-specific evidence and at some level of aggregation. Evidence for validity of a subscale is accepted at the total PROM level (conditional on it being for RA) as this could, for example, be part of a factor analysis of domain structures. Reliability must be specific to the subscale or aggregate domain, and where several studies report, for example, internal consistency reliability (α), the average of those values will be used to determine the reporting level for reliability. Evidence must be condition-specific; so, while a generic PROM may have considerable evidence of validity in other conditions or in mixed samples, if there is no specific evidence within RA, it will be rated as such.

10.1136/rmdopen-2021-001707.supp2 Supplementary data

10.1136/rmdopen-2021-001707.supp3 Supplementary data

10.1136/rmdopen-2021-001707.supp4 Supplementary data

10.1136/rmdopen-2021-001707.supp5 Supplementary data

### Psychometric evidence

An independent full-text review of each paper identified the psychometric evidence. This was collated in accord with the domains of the COnsensus-based Standards for the selection of health Measurement INstruments checklist^18^ (see online supplemental file 4 for the evidence papers associated with a given PROM), and summarised according to the OMERACT filter of truth (validity), discrimination (reliability) and feasibility (see online supplemental file 2 for this level of analysis).^19^ Consequently, evidence is collated which informs on whether the PROM is generic-specific or disease-specific, the number of items and their response options, its overall use and reliability (internal consistency, test–retest reliability, intraclass correlation coefficient and measurement error), validity (content, construct, criterion) and feasibility of use.

**Figure 2:**
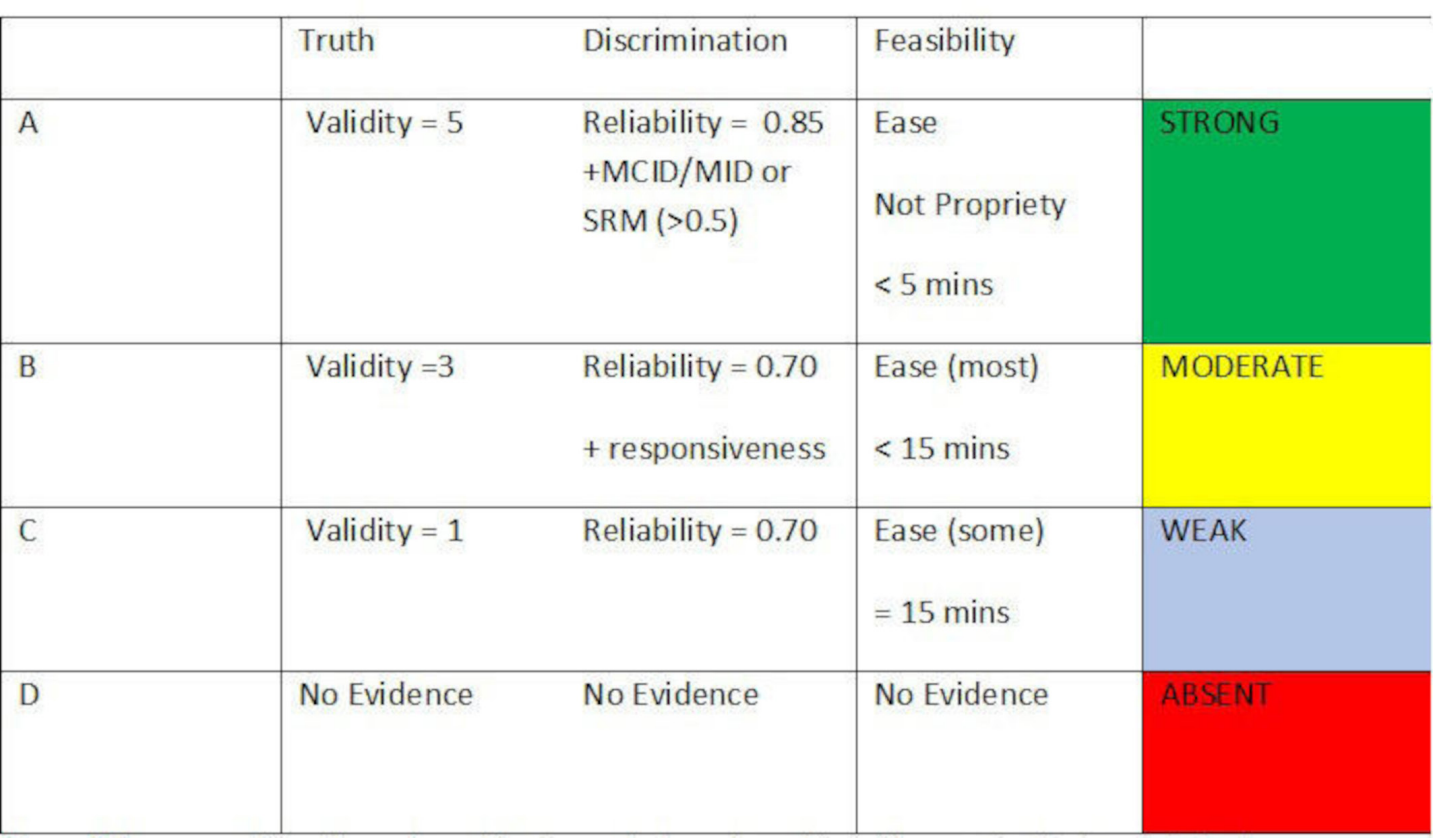
Summary of quality and quantity of reported psychometric evidence of patient-reported outcome measures (based on the OMERACT filter). Validity: quantity of evidence (this must be separate papers providing appropriate supportive evidence). Discrimination: reliability is a requirement, and reflects the degree of discrimination available. Minimally Important Difference (MID)/Minimal Clinically Important Difference (MCID) and Standardised Response Mean (SRM) regarded as best quality for responsiveness. Feasibility: understandable and quick to complete from the patient perspective. Availability irrespective of resources.

Discrimination is evaluated by the magnitude of internal consistency reliability, and whether or not some form of Minimally Important Difference (MID)/Minimal Clinically Important Difference (MCID) and Standardised Response Mean (SRM) is presented. For validity, certain PROMs may have been developed originally, for example, for arthritis in general, and subsequently validated for RA, then we designate them as a ‘hybrid’ disease-specific PROM (marked D* in the PROM-specific summary tables in online supplemental file 2). For feasibility, in the current study the focus is on how easy it is to understand and how quickly the PROM can be completed, as rated by a patient research partner educated in research by the Swedish patient organisation. This, together with the proprietary status of the PROM allows for summarising under the feasibility aspect of the OMERACT filter. The summary is presented in a colour coded format (figure 2). Consequently, a PROM which has more than five separate pieces of evidence of validity, has both reliability and responsiveness evidence at the highest levels, can be completed in less than 5 min with ease, and is free for use in all not-for-profit settings, will be rated green on all three OMERACT filter parameters, and its summary rating will be green. If on the other hand, the PROM was proprietary, then the feasibility rating would be yellow, and so would be the summary rating, which cannot be higher than the lowest rating of any of the three filter categories.

## Results

**Figure 3:**
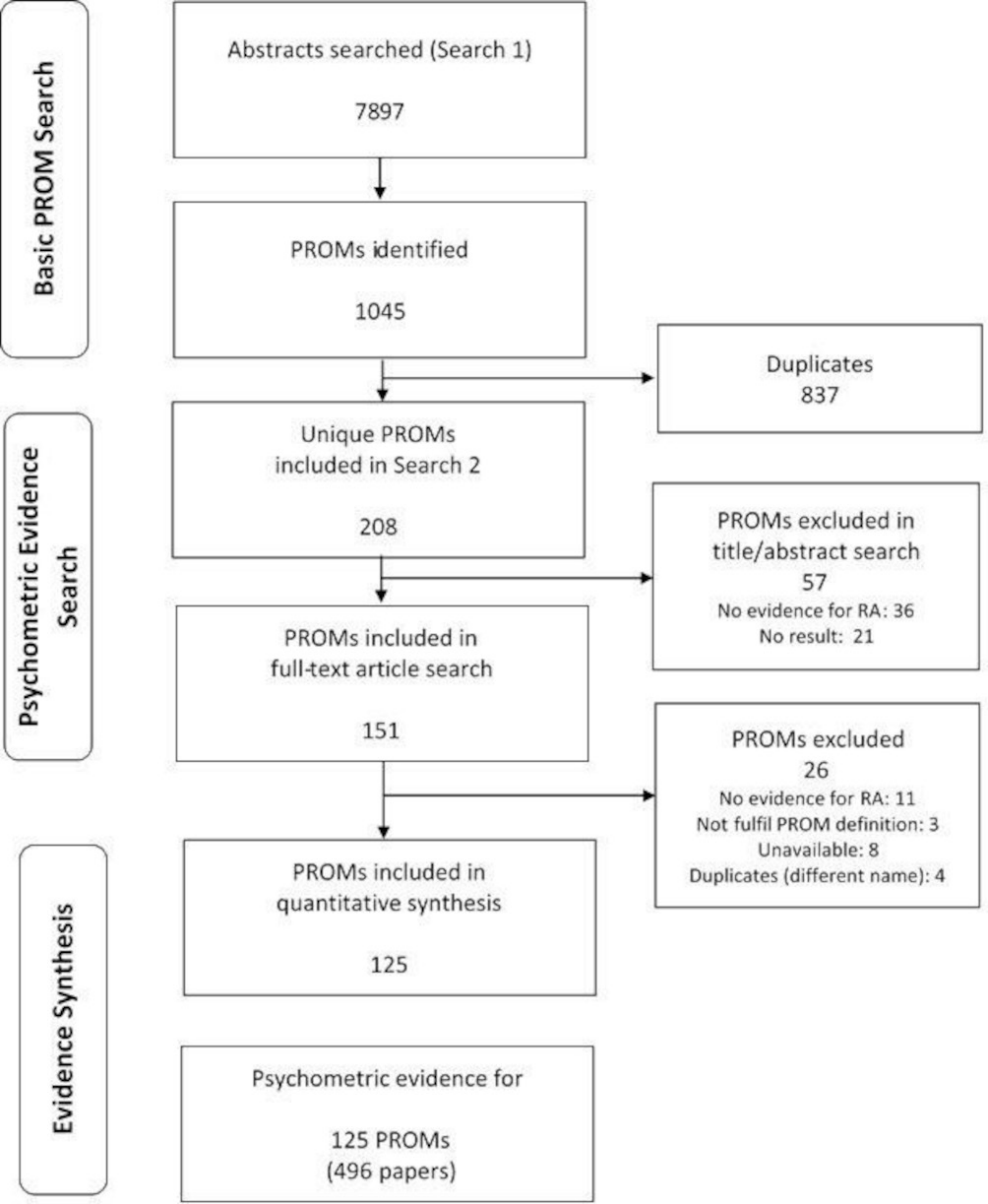
Flow diagram of search results. PROMs, patient-reported outcome measures; RA, rheumatoid arthritis.

Search 1 identified 7897 abstracts with potential PROMs (figure 3). These revealed 1045 PROMs. After excluding duplicates, 208 unique instruments satisfying the above definition of PROM were remained and put into search 2. Then 57 of these were excluded in title/abstract screening stage and further 26 in full-text screening stage due to various reasons. For example, no psychometric evidence, specific to RA, was available for 47 of these PROMs (online supplemental file 5). Some of them, such as the AUSCAN-stiffness with one item only, did not fulfil the PROM definition. Some of them were duplicates with two separate names (eg, Cochin Hand Function Scale and Duruöz Hand Index) therefore decreased to one scale in the list.

Eventually, 125 PROMs were identified where psychometric evidence was available. Given the evaluation of both subscales and total scores, this led to 263 separate assessments of scales/subscales, the overall summary of which can be seen in table 1. Most domains represented in tables 1–18 in online supplemental file 2 had one or more disease-specific PROMs. Some of them were hybrid (D*), having been developed in another condition, and then revalidated for RA.

**Table 1:** Summary of overall evidence for PROMs

Domain(s)	PROMs (n)		Strong (green)	Moderate (yellow)	Weak (blue)	Absent (red)	ICF	Detailed reporting tables in online supplemental file 2
	All	Disease-specific						
Pain	20	11	0	4	10	6	b280, b289	1
Fatigue	24	15	1	3	12	8	b130, b4552	2
Stiffness	2	1	0	0	1	1	b780	3
Emotional functions and mental health	23	13	0	10	11	2	b126, b152	4
Sleep	5	1	0	0	4	1	b134	5
Composite/other symptoms (impairments)	12	7	0	3	6	3	b, s	6
Mobility	21	18	0	4	10	7	d4	7
Self-care	12	7	0	1	8	3	d5	8
Domestic	7	7	0	1	6	0	d6	9
Work	14	11	0	3	7	4	d840–d859	10
Physical functioning	40	31	2	9	21	8	d3, d4, d5, d6	11
Social functioning	11	8	0	2	3	6	d7, d8, d9	12
Physical and social functioning (composite: impairments, activities, participation, personal, well-being)	24	16	1	2	18	3	b, d	13
Environmental	6	6	0	1	4	1	e	14
Personal (eg, self-efficacy, coping)	17	14	0	1	12	4		15
Education (knowledge and needs)	10	10	0	0	3	7		16
Quality of life, including well-being and general health	10	6	0	2	5	3		17
Health utilities	5	2	0	0	4	1		18
Total	263	184	4	46	145	68		

ICF, International Classification of Functioning; PROM, patient-reported outcome measure.

**Table 2:** Fifteen most frequently used PROMs in rheumatoid arthritis published papers: 2000–2019

No	Name	Acronym	Reference (online supplemental file 3)
1	Health Assessment Questionnaire	HAQ	71
2	Medical Outcomes Study Short Form 36-Item	SF-36	18
3	EuroQol	EQ-5D	121
4	Modified Health Assessment Questionnaire	MHAQ	78
5	Routine Assessment of Patient Index Data 3	RAPID3	93
6	Multidimensional Health Assessment Questionnaire	MDHAQ	77
7	Hospital Anxiety and Depression Scale	HADS	38
8	Disabilities of the Arm, Shoulder and Hand	DASH	69
9	Rheumatoid Arthritis Quality of Life Scale	RAQoL	120
10	Western Ontario McMaster Osteoarthritis Index	WOMAC	20
11	Functional Assessment of Chronic Illness Therapy Fatigue Scale	FACIT-F	24
12	Medical Outcomes Study Short Form 6D	SF-6D	125
13	Arthritis Impact Measurement Scales 2	AIMS2	2
14	Medical Outcomes Study Short Form 12-Item	SF-12	49
15	Rheumatoid Arthritis Impact of Disease	RAID	92

A total of 496 papers were reviewed to ascertain the psychometric evidence. Some papers had evidence for more than one PROM, therefore would appear more than once. Pain, fatigue, emotional functions, mobility, physical functioning, work and personal factors (eg, self-efficacy and coping) dominated the measured domains (table 1). The domain, physical functioning, which measures two or more of the underlying domains such as self-care and mobility, included 40 scales/subscales representing the highest number among all domains.

The majority of (sub)scales in use were disease-specific, either originally designed as such, or subsequently validated for RA. The lack of ‘strong’ evidence for the PROMs, would render the classification ‘moderate’ (yellow), although this may have been affected by proprietary status. There were only two domains, fatigue and physical functioning (either alone or composite), that had PROMs with strong evidence. Almost all domains had a range of PROMs fulfilling ‘moderate’ criteria on the OMERACT filter. Likewise, emotional functions (eg, depression and anxiety) were also represented with a high number of PROMs (n=23), ten of which had moderate evidence. The domain ‘work’ was served by 14 PROMs, three of which with moderate level of evidence. In contrast, the domains of stiffness, sleep, education and health utility were poorly served with respect to psychometric evidence.

Table 2 shows the most frequently used PROMs, dominated by the Health Assessment Questionnaire Disability Index (HAQ), the Short Form 36 (SF-36), the EuroQoL and the Modified HAQ which, taken together, appeared in more than 3500 papers, with more use during the search period than the remaining PROMs taken together. Strong psychometric evidence was found for only the HAQ, SF-36_Physical Functioning and SF-36_Vitality subscales. Those scales listed in table 2 show the dominance of physical functioning in its various guises, with only the Hospital Anxiety and Depression Scale (HADS), for anxiety and depression, and the Rheumatoid Arthritis Quality of Life Scale for needs-based quality of life offering a different focus. It is interesting to find Western Ontario McMaster Osteoarthritis Index (WOMAC), a disease-specific PROM for osteoarthritis, as being one of the most used scales in RA. For WOMAC, there are only two papers of psychometric evidence showing only weak validity evidence for pain and function subscales in RA (see online supplemental file 4). Despite this, it has been used 57 times during the search period (online supplemental file 2) and almost all of these papers are about surgery of the knee and hip in patients with RA. This finding highlights the fact that WOMAC is commonly used in RA for evaluating outcomes of lower extremity function after knee and hip surgery.

Domain-specific assessments are presented in tables 1–18 in online supplemental file 2. Where the domain-specific evidence is obtained from a subscale, this is indicated as such within parentheses. Otherwise, the PROM is designated ‘Total’ to indicate the evidence arises from the total score. In these tables, ‘Use’ represents the number of identified studies reporting use of the PROM in patients with RA. The PROM-specific references are presented in online supplemental file 3, and the papers contributing to the detailed psychometric evidence in online supplemental file 4, catalogued in the same order as the PROM-specific references.

## Discussion

In papers published from 2000 to 2019, 125 PROMs were found with some psychometric evidence, and these were categorised based on the variety of commonly used domains. Almost all domains included at least one PROM rated as at least ‘moderate’ (yellow) on the OMERACT filter summary.

The most dominant domains were those of pain (ICF-b280), fatigue (b1300, b4552), emotional functions (b152), physical functioning (d4–d6) either as a composite or as its domains, for example, mobility (d4), along with work (d8451). This is not surprising as these are the common aspects of RA which patients report, and include potentially modifying factors relevant for intervention.^20–22^ Therefore, these domains represent good candidates for consideration in various studies along with the quality of life, which is also regarded as an important domain from a ‘whole person’ perspective.^23 24^

There are particular issues related to the inclusion of PROMs in Randomised Controlled Trials (RCTs), routine clinical practice and observational epidemiological studies. Regarding the RCTs, it has been argued that the selection of PROs for trials depends on the study objective as well as the viewpoint of the stakeholder.^25^ It is further argued that there needs to be agreed on prioritisation across all stakeholders about what is most important to collect in a trial, which is why a prioritisation and selection process is necessary. For routine clinical practice, how and which PROMs should be incorporated into rheumatology practice as part of the clinical decision-making process is still thought to be controversial.^26^ Historically the HAQ has often been integrated into routine clinical monitoring.^27^ Recently the American College of Rheumatology has produced recommendations for Functional Assessment Instruments in RA suitable for routine clinical use.^28^ These include the HAQ-II, Multidimensional Health Assessment Questionnaire and the PROMIS Physical Function Short Form -10 (PROMIS PF SF-10), the former two rated yellow in the current study, the latter blue. Other PROMs, such as the HADS^29^ and the Rheumatoid Arthritis Work Instability Scale (RA-WIS),^30^ both of which have ‘cut points’ to inform potential referral, may offer useful information in a routine clinical setting.

For the future, combining PROs with technology, such as computerised adaptive testing, electronic patient-reported systems, web-based platforms and patient dashboards, could further help PROM integration into routine rheumatology clinical practice.^26^ For observational studies, the theory underlying the study is critical. The Wilson and Cleary model, which incorporates the WHO ICF model (although the former published some 6 years earlier), and extends health status (functioning) to include perceived health and perceived quality of life, provides the opportunity to examine the factors which moderate and/or mediate the relationship between health status (symptoms and functioning) and quality of life, fully operationalising a biopsychosocial perspective of the lived experience of those with RA.^4 31^ For this approach, the specification of the focal relationship (eg, the primary hypothesis) defines the type of all other third variables (eg, mediator, independent contextual variable), and so informs on the data and associated PROMs to be collected.^32 33^

The results from this review provide a domain-specific catalogue which can help in consideration of the choice of PROMs to be used. PROMs that have either yellow or green indicators will be worth considering, conditional on the year of publication. Yellow may indicate a propriety status if the feasibility indicator is also yellow, and it will be essential to check the status of any PROM to ascertain its current propriety status. If necessary, the relevant published papers listed in online supplemental file 4 can be reviewed and, if required, the detailed psychometric evidence on a PROM-specific spreadsheet can be accessed (available from the first author). As new scales and psychometric evidence for all scales are emerging continuously, a quick search to update (post-2019) the existing evidence for any chosen PROM would be wise, especially where existing evidence appears weak in the current search, and/or the scale is relatively new.

The lack of adequate PROMs to evaluate and define symptoms such as stiffness and sleep problems, is of concern, as both of these are common in RA.^34–37^ An OMERACT initiative is currently underway that hopes to address the shortfall in stiffness measurement.^38^ A PROMIS short-form for sleep disturbance (consistent with the current study definition of PROM) may offer an opportunity, although not yet apparently validated for RA.^39^ Several PROMs measuring personal factors were identified, most of which were disease-specific, but there was a disappointing level of psychometric evidence associated with those PROMs, with only one PROM achieving a moderate status. Yet concepts such as self-efficacy, resilience and coping could be hypothesised to play important moderating/mediating roles in understanding, for example, the impact of health status on the quality of life, or between, for example, functional limitations and work.^11 40 41^ In addition, only one simple summated scale the Rheumatoid Arthritis Disease Activity Index (RADAI) was found for disease activity. This is not surprising as the majority of assessments incorporate provider/physician input, and therefore would be not included as a PROM in the current study.^15^

This study was one of several EULAR funded initiatives to catalogue the available PROMs across several rheumatic diseases, leading to the EULAR Outcome Measures Library.^42 43^ There were several limitations to the current study. For example, PROMs are usually not administered to patients at the subscale level, and so the judgement is always based on the full PROM from the patient perspective. Only the reliability evidence was subscale specific in this study. Validity was usually judged by the whole PROM, but sometimes the evidence was also available about a related subscale. It is important to note that for some scales in RA the total scale and its subscales do not have the same psychometric properties. For example, the World Health Organisation Disability Assessment Schedule (WHODAS-II) total is blue whereas its subscales have red, also the Bristol Rheumatoid Arthritis Fatigue Multidimensional Questionnaire (BRAF-MDQ) and Arthritis Impact Measurement Scales have different psychometric properties for their subscales and total scores. In addition, no attempt was made to judge the quality of the evidence presented, just the weight of evidence in support of the PROM in the case of truth (validity). Finally, the feasibility was judged by just one person with RA.

Some confusion also arose concerning the SF-36. It was originally developed by the RAND corporation and known as the RAND-Short Form 36 (RAND-36), being a short form of much longer survey. It subsequently became known as the SF-36, with items identical to the RAND-36, but where the scoring of the general health and pain scales differed.^44^ Both are free for use and the latter is still in widespread use.^45^ Subsequently, the SF-36 was revised and became the SF-36 V.2.0 which is propriety. It has a recall period of both 4 weeks (standard) and 1 week (acute), rather than just the 4 weeks in the earlier version. In the literature, it is quite often difficult to ascertain which version of the SF-36 has been used. The psychometric summary of the SF-36 makes no distinction between versions but is described as though it was V.1.0. In this paper, we considered RAND-36 and SF-36 as separate PROMs, given that they have separate names and scoring systems. As such there was no RA-specific psychometric evidence attributable to the RAND-36 with only one evidence paper (see online supplemental file 4) and the use count of 20 during the search period.

In conclusion, a significant array of PROMs is available to populate RCTs; routine clinical data collection and observational studies in RA. The evidence presented here provides a domain-specific catalogue of those PROMs available until 2019, but readers are encouraged to check post-2019 for any new scale or evidence emerging in their domain of interest for those PROMs already catalogued. Initiatives such as the EULAR Outcome Measures Library will further facilitate knowledge about available PROMs, and initiatives such as that from OMERACT should facilitate the development of new PROMs where shortfalls have been shown to exist.

## Data Availability

All data relevant to the study are included in the article or uploaded as supplementary information. There are no unpublished data.
