# Supplementary material for: Use and detailed metric properties of patient-reported outcome measures for rheumatoid arthritis: a systematic review covering two decades
Source: RMD Open. 2021 Aug 10;7(2):e001707. doi: 10.1136/rmdopen-2021-001707 (PMC8356163; doi:10.1136/rmdopen-2021-001707)
Supplement: Supplementary data [file rmdopen-2021-001707supp001.pdf]

("Rheumatoid arthritis"[Title/Abstract]) AND ((Reliability[Title/Abstract] OR Validity[Title/Abstract] OR Responsiveness[Title/Abstract] OR Instrumentation[Title/Abstract] OR "validation studies"[Title/Abstract] OR "reproducability of results"[Title/Abstract] OR Reproducib*[Title/Abstract] OR psychometrics [Title/Abstract] OR psychometr* [Title/Abstract] OR "observer variation" [Title/Abstract] OR reliab* [Title/Abstract] OR valid* [Title/Abstract] OR "internal consistency" [Title/Abstract] OR (cronbach*[Title/Abstract] AND (alpha[Title/Abstract] OR alphas[Title/Abstract])) OR "item correlation" [Title/Abstract] OR "item correlations" [Title/Abstract] OR "item selection" [Title/Abstract] OR "item selections" [Title/Abstract] OR "item reduction" [Title/Abstract] OR agreement [Title/Abstract] OR precision [Title/Abstract] OR imprecision [Title/Abstract] OR "precise values" [Title/Abstract] OR test--retest [Title/Abstract] OR (test AND retest) [Title/Abstract] OR (reliab* AND (test OR retest)) [Title/Abstract] OR interrater [Title/Abstract] OR inter-rater[Title/Abstract] OR intrarater[Title/Abstract] OR intra-rater [Title/Abstract] OR interobserver[Title/Abstract] OR inter-observer [Title/Abstract] OR intraobserver[Title/Abstract] OR intra-observer [Title/Abstract] OR interexaminer[Title/Abstract] OR inter-examiner[Title/Abstract] OR intraexaminer[Title/Abstract] OR intra-examiner[Title/Abstract] OR interindividual [Title/Abstract] OR inter-individual[Title/Abstract] OR intraindividual[Title/Abstract] OR intra-individual[Title/Abstract] OR interparticipant[Title/Abstract] OR inter-participant[Title/Abstract] OR intraparticipant[Title/Abstract] OR intra-participant[Title/Abstract] OR kappa[Title/Abstract] OR kappa's[Title/Abstract] OR kappas[Title/Abstract] OR "coefficient of variation"[Title/Abstract] OR repeatab*[Title/Abstract] OR ((replicab* OR repeated) [Title/Abstract] AND (measure OR measures[Title/Abstract] OR findings[Title/Abstract] OR result[Title/Abstract] OR results[Title/Abstract] OR test[Title/Abstract] OR tests[Title/Abstract])) OR generaliza* [Title/Abstract] OR generalisa*[Title/Abstract] OR concordance[Title/Abstract] OR (intraclass[Title/Abstract] AND correlation*[Title/Abstract]) OR discriminative[Title/Abstract] OR "factor analysis"[Title/Abstract] OR "factor analyses" [Title/Abstract] OR "factor structure"[Title/Abstract] OR "factor structures"[Title/Abstract] OR dimensionality[Title/Abstract] OR subscale* [Title/Abstract] OR "multitrait scaling analysis" [Title/Abstract] OR "multitrait scaling analyses" [Title/Abstract] OR "item discriminant"[Title/Abstract] OR "interscale correlation" [Title/Abstract] OR "interscale correlations" [Title/Abstract] OR ((error[Title/Abstract] OR errors[Title/Abstract]) AND (measure* [Title/Abstract] OR correlat*[Title/Abstract] OR evaluat*[Title/Abstract] OR accuracy[Title/Abstract] OR accurate[Title/Abstract] OR precision[Title/Abstract] OR mean[Title/Abstract])) OR (uncertainty[Title/Abstract] AND (measurement[Title/Abstract] OR measuring[Title/Abstract])) OR "standard error of measurement" [Title/Abstract] OR sensitiv*[Title/Abstract] OR responsive*[Title/Abstract] OR interpretab*[Title/Abstract] OR (small*[Title/Abstract] AND (real[Title/Abstract] OR detectable[Title/Abstract]) AND (change[Title/Abstract] OR difference[Title/Abstract])) OR "meaningful change" [Title/Abstract] OR "minimal important change"[Title/Abstract] OR "minimal important difference" [Title/Abstract] OR "minimally important change"[Title/Abstract] OR "minimally important difference"[Title/Abstract] OR "minimal detectable change" [Title/Abstract] OR "minimal detectable difference" [Title/Abstract] OR "minimally detectable change" [Title/Abstract] OR "minimally detectable difference"[Title/Abstract] OR "ceiling effect"[Title/Abstract] OR "floor effect"[Title/Abstract] OR "Item response model"[Title/Abstract] OR irt[Title/Abstract] OR rasch[Title/Abstract] OR "Differential item functioning"[Title/Abstract] OR dif[Title/Abstract] OR "computer adaptive testing"[Title/Abstract] OR "item bank"[Title/Abstract] OR "cross-cultural equivalence "[Title/Abstract] OR "content validity"[Title/Abstract] OR "structural validity"[Title/Abstract] OR "cross-cultural validity"[Title/Abstract] OR "criterion validity"[Title/Abstract] OR "hypotheses testing"[Title/Abstract])) AND (("2000/01/01"[Date - Create] : "2019"[Date - Create])) AND English[Language]
