# Supplementary material for: Use and detailed metric properties of patient-reported outcome measures for rheumatoid arthritis: a systematic review covering two decades
Source: RMD Open. 2021 Aug 10;7(2):e001707. doi: 10.1136/rmdopen-2021-001707 (PMC8356163; doi:10.1136/rmdopen-2021-001707)
Supplement: Supplementary data [file rmdopen-2021-001707supp002.pdf]

Supplementary File 2

Summary of PROM-Specific Evidence

Supplementary Table 1: General characteristics and summary psychometric review of all versions of identified PROMs:
Pain (b280, b289)

Name (Domain)	Acronym	First Author/ Year	REF (A)	Generic/ Disease Specific	N of items	Response Options	Recall period	Use	Psychometric Summary			
									Validity	Discrimination	Feasibility	Overall
Arthritis Impact Measurement Scales (Pain)	AIMS	Meenan RF. 1980	1	D*	4	6	Past month	8				
Arthritis Impact Measurement Scales 2 (Arthritis pain)	AIMS2	Meenan RF. 1992	2	D	5	5	Past month	52				
Australian/Canadian Hand Osteoarthritis Index (Pain)	AUSCAN	Bellamy N. 2002	3	D*	5	5, 100 (VAS)	48 hours	2				
Foot Function Index (Pain)	FFI	Budiman-Mak E. 1991	4	D	9	10	Past week	35				
Foot Health Status Questionnaire (Foot pain)	FHSQ	Bennett PJ. 1998	5	G	4	5	Past week	4				
Michigan Hand Outcomes Questionnaire (Pain)	MHQ	Chung KC. 1998	6	D*	5	5	Past week	37				
Medical Outcomes Study Pain Intensity scale (Total)	MOS-PI	Gonzalez VM. 1995	7	G	5	11/6	Previous week	1				
Nottingham Health Profile (Pain)	NHP	Hunt SM. 1985	8	D*	8	2	At the moment	38				
Oxford Elbow Score (Pain)	OES	Dawson J. 2008	9	G	4	5	Past 4 weeks	4				
PainDETECT Questionnaire (Total)	PDQ (PainDetect)	Freynhagen R. 2006	10	G	12	6, 11	4 Weeks	21				

Patient-Reported Outcomes Measurement Information System – 29 Profile (Pain Interference)	PROMIS-29-Profile	Hinchcliff M. 2011	11	G	4	5	7 days	2					
Patient-Reported Outcomes Measurement Information System Pain Intensity 3a-SF (Total)	PROMIS-PI 3a-SF	Stone AA. 2016	12	G	3	5	Past 7 days	2					
RAND 36-Item Health Survey (Pain)	RAND-36	Hays RD. 1993	13	G	2	5-6	Past 4 weeks	20					
Rheumatoid and Arthritis Outcome Score (Pain)	RAOS	Bremander AB. 2003	14	D	9	5	Last week	6					
Rheumatoid Arthritis Pain Scale (Total)	RAPS	Anderson DL. 2001	15	D	24	7	Last week	5					
Score for Assessment and Quantification of Chronic Rheumatic Affections of the Hands (Pain)	SACRAH	Leeb B. 2003	16	D	4	VAS	48 hours	2					
Self-Administered Foot Evaluation Questionnaire (Pain and Pain Related)	SAFE-Q	Niki H. 2011	17	D*	9	5	Past week	8					
Medical Outcomes Study Short Form 36-Item (Bodily Pain)	SF-36	Ware JE. 1992	18	D*	2	5	Past 4 weeks	562					
Short Form McGill Pain Questionnaire (Pain)	SF-MPQ	Melzack R. 1987	19	G	15	3-5	At the moment	7					
Western Ontario McMaster Osteoarthritis Index (Pain)	WOMAC	Bellamy N. 1988	20	G	5	5	Currently	57					

Evidence: Strong; Moderate; Weak; Absent

Supplementary Table 2: General characteristics and summary psychometric review of all versions of identified PROMs:
Fatigue (b130, b4552)

Name (Domain)	Acronym	First Author/ Year	REF (A)	Generic/ Disease Specific	N of items	Response Options	Recall period	Use	Psychometric Summary			
									Validity	Discrimination	Feasibility	Overall
Bristol Rheumatoid Arthritis Fatigue Multidimensional Questionnaire (Cognition)	BRAF-MDQ	Nicklin J. 2010	21	D	5	3, 11	Past 7 days	17				
Bristol Rheumatoid Arthritis Fatigue Multidimensional Questionnaire (Emotion)	BRAF-MDQ	Nicklin J. 2010	21	D	4	3, 11	Past 7 days	17				
Bristol Rheumatoid Arthritis Fatigue Multidimensional Questionnaire (Living)	BRAF-MDQ	Nicklin J. 2010	21	D	7	3, 11	Past 7 days	17				
Bristol Rheumatoid Arthritis Fatigue Multidimensional Questionnaire (Physical)	BRAF-MDQ	Nicklin J. 2010	21	D	4	3, 11	Past 7 days	17				
Bristol Rheumatoid Arthritis Fatigue Multidimensional Questionnaire (Total)	BRAF-MDQ	Nicklin J. 2010	21	D	20	3, 11	Past 7 days	17				
Chalder Fatigue Scale (Mental fatigue)	CFS	Chalder T. 1993	22	G	4	4	Last month	3				
Chalder Fatigue Scale (Physical fatigue)	CFS	Chalder T. 1993	22	G	7	4	Last month	3				
Chalder Fatigue Scale (Total)	CFS	Chalder T. 1993	22	G	11	4	Last month	3				
Checklist Individual Strength (Total)	CIS8R	Vercoulen J. 1994	23	G	8	7	Two weeks	10				
Functional Assessment of Chronic Illness Therapy Fatigue Scale (Total)	FACIT-F	Yellen SB. 1997	24	D*	13	5	Past 7 days	55				

Fatigue Severity Scale (Total)	FSS	Krupp LB. 1989	25	G	9	7	Last week	18
Multidimensional Assessment of Fatigue (Intensity)	MAF	Tack B. 1991	26	D	3	10	Past week	15
Multidimensional Assessment of Fatigue (Interference)	MAF	Tack B. 1991	26	D	11	10	Past week	15
Multidimensional Assessment of Fatigue (Total)	MAF	Tack B. 1991	26	D	15	4, 10	Past week	15
Multi-Dimensional Fatigue Inventory (Total)	MFI	Smets E. 1995	27	G	20	5	Lately	9
Nottingham Health Profile (Energy)	NHP	Hunt SM. 1985	8	D*	3	2	At the moment	38
Profile of Mood States - Fatigue (Total)	POMS-Fatigue	McNair DM. 1971	28	G	7	5	Past week	6
Patient-Reported Outcomes Measurement Information System Fatigue SF 4a (Total)	PROMIS Fatigue 4a-SF	Bingham III CO. 2019	29	D*	4	5	Past 7 days	1
Patient-Reported Outcomes Measurement Information System Fatigue SF 7a (Total)	PROMIS Fatigue 7a-SF	Bingham III CO. 2019	30	D*	7	5	Past 7 days	2
Patient-Reported Outcomes Measurement Information System Fatigue SF 8a (Total)	PROMIS Fatigue 8a-SF	Bingham III CO. 2019	31	D*	8	5	Past 7 days	2
Patient-Reported Outcomes Measurement Information System – 29 Profile (Fatigue)	PROMIS-29-Profile	Hinchcliff M. 2011	11	G	4	5	7 days	2
RAND 36-Item Health Survey (Vitality)	RAND-36	Hays RD. 1993	13	G	4	6	Past 4 weeks	20

Medical Outcomes Study Short Form 36-Item (Vitality)	SF-36	Ware JE. 1992	18	D*	4	5	Past 4 weeks	562					
Subjective Vitality Scale (Total)	SVS	Ryan RM. 1997	32	D*	7	7	Now	1					

Evidence: Strong; Moderate; Weak; Absent

Supplementary Table 3: General characteristics and summary psychometric review of all versions of identified PROMs:
Stiffness (b780)

Name (Domain)	Acronym	First Author/ Year	REF (A)	Generic/ Disease Specific	N of items	Response Options	Recall period	Use	Psychometric Summary			
									Validity	Discrimination	Feasibility	Overall
Score for Assessment and Quantification of Chronic Rheumatic Affections of the Hands (Stiffness)	SACRAH	Leeb B. 2003	16	D	2	0-100	48 hours	2				
Western Ontario McMaster Osteoarthritis Index (Stiffness)	WOMAC	Bellamy N. 1988	20	G	2	5	Currently	57				

Evidence: Strong; Moderate; Weak; Absent

Supplementary Table 4: General characteristics and summary psychometric review of all versions of identified PROMs:
Emotional functions and mental health (b152, b126)

Name (Domain)	Acronym	First Author/ Year	REF (A)	Generic/ Disease Specific	N of items	Response Options	Recall period	Use	Psychometric Summary			
									Validity	Discrimination	Feasibility	Overall
Arthritis Impact Measurement Scales (Anxiety)	AIMS	Meenan RF. 1980	1	D*	6	2-6	Past month	8				
Arthritis Impact Measurement Scales (Depression)	AIMS	Meenan RF. 1980	1	D*	6	2-6	Past month	8				
Arthritis Impact Measurement Scales 2 (Mood)	AIMS2	Meenan RF. 1992	2	D	5	5	Past month	52				
Arthritis Impact Measurement Scales 2 (Level of tension)	AIMS2	Meenan RF. 1992	2	D	5	5	Past month	52				
Arthritis Impact Measurement Scales 2-SF (Affect)	AIMS2-SF	Guillemin F. 1997	33	D	5	5	Past 4 weeks	14				
Center for Epidemiologic Studies Depression Scale (Total)	CES-D	Radloff LS. 1977	34	G	20	4	Past week	28				
Cedars-Sinai Health-Related Quality of Life for Rheumatoid Arthritis Instrument (Emotional well-being)	CSHQ-RA	Weisman MH. 2003	35	D	8	5	Past 4 weeks	5				
Depression, Anxiety and Stress Scale (Anxiety)	DASS	Lovibond SH. 1995	36	G	14	4	Past week	3				
Depression, Anxiety and Stress Scale (Depression)	DASS	Lovibond SH. 1995	36	G	14	4	Past week	3				
Depression, Anxiety and Stress Scale (Stress)	DASS	Lovibond SH. 1995	36	G	14	4	Past week	3				
Emotional Intimacy Scale (Total)	EIS	Sinclair VG. 2005	37	D	5	5	Currently	1				

Hospital Anxiety and Depression Scale (Anxiety)	HADS	Zigmond AS. 1983	38	G	7	4	Past week	81				
Hospital Anxiety and Depression Scale (Depression)	HADS	Zigmond AS. 1983	38	G	7	4	Past week	81				
Nottingham Health Profile (Emotional reactions)	NHP	Hunt SM. 1985	8	D*	9	2	At the moment	38				
Nottingham Health Profile (Social isolation)	NHP	Hunt SM. 1985	8	D*	5	2	At the moment	38				
Patient-Reported Outcomes Measurement Information System – 29 Profile (Anxiety)	PROMIS-29-Profile	Hinchcliff M. 2011	11	G	4	5	7 days	2				
Patient-Reported Outcomes Measurement Information System – 29 Profile (Depression)	PROMIS-29-Profile	Hinchcliff M. 2011	11	G	4	5	7 days	2				
Psychological Vulnerability Scale (Total)	PVS	Sinclair VG. 1999	39	D	6	5	Not specified	1				
RAND 36-Item Health Survey (Emotional well-being)	RAND-36	Hays RD. 1993	13	G	5	6	Past 4 weeks	20				
RAND 36-Item Health Survey (Role emotional)	RAND-36	Hays RD. 1993	13	G	3	2	Past 4 weeks	20				
Medical Outcomes Study Short Form 36-Item (Mental Health)	SF-36	Ware JE. 1992	18	D*	5	5	Past 4 weeks	562				
Medical Outcomes Study Short Form 36-Item (Role-Emotional)	SF-36	Ware JE. 1992	18	D*	3	5	Past 4 weeks	562				
World Health Organisation Quality of Life Instrument Short Form (Psychological)	WHOQoL-Bref	Harper H. 1998	40	D*	6	5	Past 4 weeks	15				

Evidence: Strong; Moderate; Weak; Absent

Supplementary Table 5: General characteristics and summary psychometric review of all versions of identified PROMs:
Sleep (b134)

Name (Domain)	Acronym	First Author/ Year	REF (A)	Generic/ Disease Specific	N of items	Response Options	Recall period	Use	Psychometric Summary			
									Validity	Discrimination	Feasibility	Overall
Athens Insomnia Scale (Total)	AIS	Soldatos CR. 2000	41	G	8	4	Last month	7				
Jenkins Sleep Evaluation Scale (Total)	JSS	Jenkins DC. 1988	42	G	4	6	30 days	1				
Medical Outcomes Study Sleep Questionnaire (Sleep disturbance scale)	MOS-Sleep	Hays RD. 1992	43	G	4	5-6	Past 4 weeks	12				
Nottingham Health Profile (Sleep)	NHP	Hunt SM. 1985	8	D*	5	2	At the moment	38				
Patient-Reported Outcomes Measurement Information System – 29 Profile (Sleep Disturbance)	PROMIS-29-Profile	Hinchcliff M. 2011	11	G	4	5	7 days	2				

Evidence: Strong; Moderate; Weak; Absent

Supplementary Table 6: General characteristics and summary psychometric review of all versions of identified PROMs:
Composite/Other symptoms (Impairments) (b, s)

Name (Domain)	Acronym	First Author/ Year	REF (A)	Generic/ Disease Specific	N of items	Response Options	Recall period	Use	Psychometric Summary			
									Validity	Discrimination	Feasibility	Overall
Arthritis Body Experience Scale (Body Physical Function)	ABES	Williams B. 2001	44	G	2	10	Not specified	1				
Arthritis Body Experience Scale (Body Self Consciousness)	ABES	Williams B. 2001	44	G	3	10	Not specified	1				
Arthritis Body Experience Scale (Body Totality)	ABES	Williams B. 2001	44	G	4	10	Not specified	1				
Arthritis Impact Measurement Scales 2-SF (Symptoms: Pain and stiffness)	AIMS2-SF	Guillemin F. 1997	33	D	3	5	Past 4 weeks	14				
Foot Impact Scale (Impairment Footwear)	FIS	Helliwell P. 2005	45	D	21	2	At the moment	20				
Michigan Hand Outcomes Questionnaire (Aesthetics)	MHQ	Chung KC. 1998	6	D*	4	5	Past week	37				
Perceived Deficits Questionnaire - Short Form (Total)	PDQ-SF	Sullivan JJ. 1990	46	G	5	5	Past week	1				
Rheumatoid Arthritis Disease Activity Index-5	RADAI-5	Leeb BF.	47	D	5	0-10	6 months, today, yesterday morning	13				
Rheumatoid and Arthritis Outcome Score (Symptoms)	RAOS	Bremander AB. 2003	14	D	7	5	Last week	6				

Rheumatoid Arthritis Symptom Questionnaire (Total)	RASQ	Banderas B. 2017	48	D	8	11	7 days	2					
Self-Administered Foot Evaluation Questionnaire (Shoe Related)	SAFE-Q	Niki H. 2011	17	D*	3	5	Past week	8					
Medical Outcomes Study Short Form 12-Item (Mental component)	SF-12	Ware JE. 1996	49	G	6	2-6	Four weeks	44					

Evidence: Strong; Moderate; Weak; Absent

Supplementary Table 7: General characteristics and summary psychometric review of all versions of identified PROMs:
Mobility (d4)

Name (Domain)	Acronym	First Author/ Year	REF (A)	Generic/ Disease Specific	N of items	Response Options	Recall period	Use	Psychometric Summary			
									Validity	Discrimination	Feasibility	Overall
Arthritis Impact Measurement Scales (Dexterity)	AIMS	Meenan RF. 1980	1	D*	5	2-6	Past month	8				
Arthritis Impact Measurement Scales (Mobility)	AIMS	Meenan RF. 1980	1	D*	4	2-6	Past month	8				
Arthritis Impact Measurement Scales (Physical activity)	AIMS	Meenan RF. 1980	1	D*	5	2-6	Past month	8				
Arthritis Impact Measurement Scales 2 (Arm function)	AIMS2	Meenan RF. 1992	2	D	5	5	Past month	52				
Arthritis Impact Measurement Scales 2 (Hand and finger function)	AIMS2	Meenan RF. 1992	2	D	5	5	Past month	52				
Arthritis Impact Measurement Scales 2 (Mobility level)	AIMS2	Meenan RF. 1992	2	D	5	5	Past month	52				
Arthritis Impact Measurement Scales 2 (Walking and bending)	AIMS2	Meenan RF. 1992	2	D	5	5	Past month	52				
Australian/Canadian Hand Osteoarthritis Index (Function)	AUSCAN	Bellamy N. 2002	3	D*	9	5	48 hours	2				
Cedars-Sinai Health-Related Quality of Life for Rheumatoid Arthritis Instrument (Dexterity)	CSHQ-RA	Weisman MH. 2003	35	D	7	5	Past 4 weeks	5				
Cedars-Sinai Health-Related Quality of Life for Rheumatoid Arthritis Instrument (Mobility)	CSHQ-RA	Weisman MH. 2003	35	D	8	5	Past 4 weeks	5				

Evaluation of daily activity questionnaire (Moving & transfers)	EDAQ	Nordenskiöld U. 1996	50	D	6	4	Last 2 weeks	3					
Evaluation of daily activity questionnaire (Moving around indoors)	EDAQ	Nordenskiöld U. 1996	50	D	12	4	Last 2 weeks	3					
Foot Function Index (Disability)	FFI	Budiman-Mak E. 1991	4	D	9	10	Past week	35					
Foot Health Status Questionnaire (Foot function)	FHSQ	Bennett PJ. 1998	5	G	4	5	Past week	4					
Foot Impact Scale (Activities-Participation)	FIS	Helliwell P. 2005	45	D	30	2	At the moment	20					
International Physical Activity Questionnaire Short Form (Total)	IPAQ-SF	Craig CL. 2003	51	G	7	3	Last 7 days	1					
Michigan Hand Outcomes Questionnaire (Overall hand function)	MHQ	Chung KC. 1998	6	D*	5	5	Past week	37					
Rheumatoid and Arthritis Outcome Score (Sports/Recreation)	RAOS	Bremander AB. 2003	14	D	5	5	Last week	6					
Recent-Onset Arthritis Disability Index (Lower extremity function)	ROAD	Salaffi F. 2005	52	D	4	5	One week	6					
Recent-Onset Arthritis Disability Index (Upper extremity function)	ROAD	Salaffi F. 2005	52	D	5	5	One week	6					
World Health Organisation Disability Assessment Schedule –II (Getting around)	WHODAS-II	WHO 2004	53	G	5	5	Last 30 days	5					

Evidence: Strong; Moderate; Weak; Absent

Supplementary Table 8: General characteristics and summary psychometric review of all versions of identified PROMs:
Self-Care (d5)

Name (Domain)	Acronym	First Author/ Year	REF (A)	Generic/ Disease Specific	N of items	Response Options	Recall period	Use	Psychometric Summary			
									Validity	Discrimination	Feasibility	Overall
Arthritis Impact Measurement Scales 2 (Self-care)	AIMS2	Meenan RF. 1992	2	D	4	5	Past month	52				
Compliance Questionnaire-Rheumatology (Total)	CQR	de Klerk E. 1999	54	G	19	4	Not specified	23				
Compliance Questionnaire-Rheumatology - 5 Item Version (Total)	CQR-5	Hughes LD. 2013	55	D	5	4	Not specified	3				
Evaluation of daily activity questionnaire (Bathing/showering)	EDAQ	Nordenskiöld U. 1996	50	D	11	4	Last 2 weeks	3				
Evaluation of daily activity questionnaire (Eating/drinking)	EDAQ	Nordenskiöld U. 1996	50	D	10	4	Last 2 weeks	3				
Evaluation of daily activity questionnaire (Getting dressed/undressed)	EDAQ	Nordenskiöld U. 1996	50	D	11	4	Last 2 weeks	3				
Evaluation of daily activity questionnaire (In the bathroom / personal care)	EDAQ	Nordenskiöld U. 1996	50	D	12	4	Last 2 weeks	3				
Medication Adherence Report Scale-5 (Total)	MARS-5	Horne R. 2002	56	G	5	5	Not specified	6				
Medication Adherence Report Scale-9 (Total)	MARS-9	Salt E. 2012	57	G	9	5	Not specified	1				
Medication Adherence Scale (Total)	MAS	Morisky DE. 1986	58	G	4	2	Not specified	10				

Shoulder Function Assessment Scale (ADL)	SFA	Ende van den CHM. 1996	59	D	4	5	Not specified	3					
World Health Organisation Disability Assessment Schedule –II (Self-care)	WHODAS-II	WHO 2004	53	G	4	5	Last 30 days	5					

Evidence: Strong; Moderate; Weak; Absent

Supplementary Table 9: General characteristics and summary psychometric review of all versions of identified PROMs:
Domestic (d6)

Name (Domain)	Acronym	First Author/ Year	REF (A)	Generic/ Disease Specific	N of items	Response Options	Recall period	Use	Psychometric Summary			
									Validity	Discrimination	Feasibility	Overall
Arthritis Impact Measurement Scales (Household activity)	AIMS	Meenan RF. 1980	1	D*	7	2-6	Past month	8				
Arthritis Impact Measurement Scales 2 (Household tasks)	AIMS2	Meenan RF. 1992	2	D	4	5	Past month	52				
Evaluation of daily activity questionnaire (Caring)	EDAQ	Nordenskiöld U. 1996	50	D	9	4	Last 2 weeks	3				
Evaluation of daily activity questionnaire (Cleaning the house)	EDAQ	Nordenskiöld U. 1996	50	D	9	4	Last 2 weeks	3				
Evaluation of daily activity questionnaire (Cooking)	EDAQ	Nordenskiöld U. 1996	50	D	14	4	Last 2 weeks	3				
Evaluation of daily activity questionnaire (Gardening & household maintenance)	EDAQ	Nordenskiöld U. 1996	50	D	7	4	Last 2 weeks	3				
Evaluation of daily activity questionnaire (Laundry & clothes care)	EDAQ	Nordenskiöld U. 1996	50	D	9	4	Last 2 weeks	3				

Evidence: Strong; Moderate; Weak; Absent

Supplementary Table 10: General characteristics and summary psychometric review of all versions of identified PROMs:
Work (d840-d859)

Name (Domain)	Acronym	First Author/ Year	REF (A)	Generic/ Disease Specific	N of items	Response Options	Recall period	Use	Psychometric Summary			
									Validity	Discrimination	Feasibility	Overall
Arthritis Impact Measurement Scales 2 (Work)	AIMS2	Meenan RF. 1992	2	D	5	5	Past month	52				
Arthritis Impact Measurement Scales 2-SF (Work)	AIMS2-SF	Guillemin F. 1997	33	D	2	5	Past 4 weeks	14				
Endicott Work Productivity Scale (Total)	EWPS	Endicott J. 1997	60	G	25	5	One week	2				
Michigan Hand Outcomes Questionnaire (Work performance)	MHQ	Chung KC. 1998	6	D*	5	5	Past week	37				
Rheumatoid Arthritis Work Instability Scale (Total)	RA-WIS	Gilworth G. 2003	61	D	23	2	At the moment	22				
Stanford Presenteeism Scale (Total)	SPS-6	Koopman C. 2002	62	G	6	5	Past month	3				
Valuation of Lost Productivity Questionnaire (Total)	VOLP	Zhang W. 2011	63	D	6	2-6	Currently	2				
Workplace Activity Limitations Scale (Total)	WALS	Gignac MA. 2004	64	G	12	4	In general	4				
Work Functioning Impairment Scale (Total)	Wfun	Fujino Y. 2015	65	D*	7	5	Currently	2				
Work Limitations Questionnaire (Mental-interpersonal demands)	WLQ	Lerner D. 2001	66	D*	9	5	Past 2 weeks	17				

Work Limitations Questionnaire (Output demands)	WLQ	Lerner D. 2001	66	D*	5	5	Past 2 weeks	17					
Work Limitations Questionnaire (Physical demands)	WLQ	Lerner D. 2001	66	D*	6	5	Past 2 weeks	17					
Work Limitations Questionnaire (Time management)	WLQ	Lerner D. 2001	66	D*	5	5	Past 2 weeks	17					
Work Limitations Questionnaire (Total)	WLQ	Lerner D. 2001	66	D*	25	5	Past 2 weeks	17					

Evidence: Strong; Moderate; Weak; Absent

Supplementary Table 11: General characteristics and summary psychometric review of all versions of identified PROMs:
Physical functioning (d3, d4, d5, d6)

Name (Domain)	Acronym	First Author/ Year	REF (A)	Generic/ Disease Specific	N of items	Response Options	Recall period	Use	Psychometric Summary			
									Validity	Discrimination	Feasibility	Overall
ABILHAND (Total)	ABILHAND	Penta M. 1998	67	G	27	3	Within the last three months	6				
Arthritis Impact Measurement Scales (ADL)	AIMS	Meenan RF. 1980	1	D*	4	2-6	Past month	8				
Arthritis Impact Measurement Scales 2-SF (Physical: Self-care and mobility)	AIMS2-SF	Guillemin F. 1997	33	D	12	5	Past 4 weeks	14				
Cochin Hand Function Scale (Total)	CHFS	Duruöz M. 1996	68	D	18	6	Not specified	16				
Disabilities of the Arm, Shoulder and Hand (Total)	DASH	Hudak PL. 1996	69	G	30	5	1 week	62				
Evaluation of daily activity questionnaire (Communication)	EDAQ	Nordenskiöld U. 1996	50	D	6	4	Last 2 weeks	3				
Evaluation of daily activity questionnaire (Moving around outdoors / shopping)	EDAQ	Nordenskiöld U. 1996	50	D	13	4	Last 2 weeks	3				
Foot Function Index (Activity Limitation)	FFI	Budiman-Mak E. 1991	4	D	5	10	Past week	35				
Foot Function Index – Revised Short Form (Total)	FFI-RS	Budiman-Mak E. 2006	70	D	34	4	Past week	1				
Health Assessment Questionnaire (Total)	HAQ	Fries JF. 1980	71	D	20	4	Past week	2556				
Health Assessment Questionnaire-II (Total)	HAQ-II	Wolfe F. 2004	72	D	10	4	One week	17				

Joint Protection Behaviour Short Form (Total)	JPBA-S	Hammond A. 1999	73	D	10	5	At the moment	2					
McMaster Toronto Arthritis Patient Preference Disability Questionnaire (Total)	MACTAR	Tugwell P. 1987	74	D	5	3	Past 2 weeks	11					
Measure of Activity Limitation (Total)	MAL	Goodacre L. 2007	75	D	15	5	Two weeks	1					
Measure of Activity Performance of the Hand (Total)	MAP-HAND	Paulsen T. 2010	76	D	18	5	Last time	3					
Multidimensional Health Assessment Questionnaire (Total)	MDHAQ	Pincus T. 1999	77	D*	10	4	Past week	98					
Modified Health Assessment Questionnaire (Total)	MHAQ	Pincus T. 1983	78	D*	8	4	Past week	206					
Michigan Hand Outcomes Questionnaire (Activities of daily living)	MHQ	Chung KC. 1998	6	D*	12	5	Past week	37					
Nottingham Health Profile (Physical mobility)	NHP	Hunt SM. 1985	8	D*	8	2	At the moment	38					
Oxford Elbow Score (Function)	OES	Dawson J. 2008	9	G	4	5	Past 4 weeks	4					
Personal Impact Health Assessment Questionnaire (Total)	PI-HAQ	Hewlett S. 2002	79	D	8	4	One week	2					
Patient-Reported Outcomes Measurement Information System – 29 Profile (Physical Function)	PROMIS-29-Profile	Hinchcliff M. 2011	11	G	4	5	7 days	2					

Patient-Reported Outcomes Measurement Information System – 29 Profile (Social Roles and Activities)	PROMIS-29-Profile	Hinchcliff M. 2011	11	G	4	5	7 days	2					
Patient-Reported Outcomes Measurement Information System (PROMIS) physical function form (PF-10a) (Total)	PROMIS-PF 10a-SF	Fries JF. 2009	80	D*	10	5	Now	7					
Patient-Reported Outcomes Measurement Information System (PROMIS) physical function form (PF-20) (Total)	PROMIS-PF 20-SF	Fries JF. 2009	81	D*	20	5	Now	5					
Performance and Satisfaction in Activities of Daily Living (Performance)	PS-ADL	Archenholtz B. 2008	82	D	12	4	Past week	1					
Performance and Satisfaction in Activities of Daily Living (Satisfaction)	PS-ADL	Archenholtz B. 2008	82	D	12	3	Past week	1					
Quick Disabilities of the Arm, Shoulder and Hand (DASH) Questionnaire (Total)	QuickDASH	Hammond A. 2018	83	D*	11	5	Last Week	21					
RAND 36-Item Health Survey (Physical functioning)	RAND-36	Hays RD. 1993	13	G	10	3	Past 4 weeks	20					
RAND 36-Item Health Survey (Role physical)	RAND-36	Hays RD. 1993	13	G	4	2	Past 4 weeks	20					
Rheumatoid and Arthritis Outcome Score (ADL)	RAOS	Bremander AB. 2003	14	D	17	5	Last week	6					

Rheumatoid Hand Disability Scale (Total)	RHDA	Guermazi M. 2004	84	D	10	4	Not specified	1					
Recent-Onset Arthritis Disability Index (Activities of daily living/work)	ROAD	Salaffi F. 2005	52	D	3	5	One week	6					
Recent-Onset Arthritis Disability Index (Total)	ROAD	Salaffi F. 2005	52	D	12	5	One week	6					
Score for Assessment and Quantification of Chronic Rheumatic Affections of the Hands (Function)	SACRAH	Leeb B. 2003	16	D	17	0-100	48 hours	2					
Self-Administered Foot Evaluation Questionnaire (Physical Functioning and Daily Living)	SAFE-Q	Niki H. 2011	17	D*	11	5	Past week	8					
Medical Outcomes Study Short Form 36-Item (Physical functioning)	SF-36	Ware JE. 1992	18	D*	10	3	Past 4 weeks	562					
Medical Outcomes Study Short Form 36-Item (Role-Physical)	SF-36	Ware JE. 1992	18	D*	4	5	Past 4 weeks	562					
World Health Organisation Disability Assessment Schedule –II (Life activities)	WHODAS-II	WHO 2004	53	G	8	5	Last 30 days	5					
Western Ontario McMaster Osteoarthritis Index (Function)	WOMAC	Bellamy N. 1988	20	G	17	5	Currently	57					

Evidence: Strong; Moderate; Weak; Absent

Supplementary Table 12: General characteristics and summary psychometric review of all versions of identified PROMs:
Social functioning (d7, d8, d9)

Name (Domain)	Acronym	First Author/ Year	REF (A)	Generic/ Disease Specific	N of items	Response Options	Recall period	Use	Psychometric Summary			
									Validity	Discrimination	Feasibility	Overall
Arthritis Impact Measurement Scales (Social activity)	AIMS	Meenan RF. 1980	1	D*	4	2-6	Past month	8				
Arthritis Impact Measurement Scales 2 (Social activities)	AIMS2	Meenan RF. 1992	2	D	5	5	Past month	52				
Arthritis Impact Measurement Scales 2-SF (Social)	AIMS2-SF	Guillemin F. 1997	33	D	4	5	Past 4 weeks	14				
Evaluation of daily activity questionnaire (Hobbies, leisure & social activities)	EDAQ	Nordenskiöld U. 1996	50	D	9	4	Last 2 weeks	3				
Patient Specific Leisure Scale (Total)	PSLS	Wikström I. 2009	85	D	6	11	Today	1				
RAND 36-Item Health Survey (Social functioning)	RAND-36	Hays RD. 1993	13	G	2	5	Past 4 weeks	20				
Self-Administered Foot Evaluation Questionnaire (Social Functioning)	SAFE-Q	Niki H. 2011	17	D*	6	5	Past week	8				
Medical Outcomes Study Short Form 36-Item (Social Functioning)	SF-36	Ware JE. 1992	18	D*	2	5	Past 4 weeks	562				
World Health Organisation Disability Assessment Schedule –II (Getting along with people)	WHODAS-II	WHO 2004	53	G	5	5	Last 30 days	5				

World Health Organisation Disability Assessment Schedule –II (Participation in society)	WHODAS-II	WHO 2004	53	G	8	5	Last 30 days	5						
World Health Organisation Quality of Life Instrument Short Form (Social relationships)	WHOQoL-Bref	Harper H. 1998	40	D*	3	5	Past 4 weeks	15						

Evidence: Strong; Moderate; Weak; Absent

Supplementary Table 13: General characteristics and summary psychometric review of all versions of identified PROMs:
Physical and social functioning (Composite: impairments, activities, participation, personal, well-being)

Name (Domain)	Acronym	First Author/ Year	REF (A)	Generic/ Disease Specific	N of items	Response Options	Recall period	Use	Psychometric Summary			
									Validity	Discrimination	Feasibility	Overall
Patient Reported Outcomes Thermometer (Total)	5T-PROs	Salaffi F. 2018	86	G	5	11	Last Week	1				
Arthritis Impact Measurement Scales (Total)	AIMS	Meenan RF. 1980	1	D*	46	2-6	Past month	8				
Cedars-Sinai Health-Related Quality of Life for Rheumatoid Arthritis Instrument (Physical activity)	CSHQ-RA	Weisman MH. 2003	35	D	8	5	Past 4 weeks	5				
Cedars-Sinai Health-Related Quality of Life for Rheumatoid Arthritis Instrument (Sexual well-being)	CSHQ-RA	Weisman MH. 2003	35	D	2	5	Past 4 weeks	5				
Cedars-Sinai Health-Related Quality of Life for Rheumatoid Arthritis Instrument Short Form (Total)	CSHQ-RA-SF	Chiou CF. 2004	87	D	11	5	Four weeks	2				
Foot Function Index (Total)	FFI	Budiman-Mak E. 1991	4	D	23	10	Past week	35				
The Flare Instrument (Total)	Flare-RA	Berthelot JM. 2012	88	D	13	11	Last month	7				
Michigan Hand Outcomes Questionnaire (Total)	MHQ	Chung KC. 1998	6	D*	37	5	Past week	37				
Modified Score for the Assessment and Quantification of Chronic Rheumatic Affections of the Hands (Total)	M-SACRAH	Sautner J. 2004	89	D	12	0-100	48 hours	6				

Musculoskeletal Health Questionnaire (Total)	MSK-HQ	Hill JC. 2016	90	G	14	5	Past 2 weeks	1
Oxford Elbow Score (Social-psychological)	OES	Dawson J. 2008	9	G	4	5	Past 4 weeks	4
Rheumatoid Arthritis Flare Questionnaire (Total)	RA-FQ	Bartlett SJ. 2017	91	D	5	11	7 days	1
Rheumatoid Arthritis Impact of Disease (Total)	RAID	Gossec L. 2009	92	D	7	11	Past week	39
Routine Assessment of Patient Index Data 3 (Total)	RAPID3	Pincus T. 2009	93	D	15	4/21	At this time	113
Score for Assessment and Quantification of Chronic Rheumatic Affections of the Hands (Total)	SACRAH	Leeb B. 2003	16	D	23	0-100	48 hours	2
Medical Outcomes Study Short Form 12-Item (Physical component)	SF-12	Ware JE. 1996	49	G	6	2-6	Four weeks	44
Short Form Score for the Assessment and Quantification of Chronic Rheumatic Affections of the Hands (Total)	SF-SACRAH	Rintelen B. 2009	94	D	5	11	Two days	3
Short Musculoskeletal Function Assessment Questionnaire (Bother index)	SMFA	Swiontkowski MF. 1999	95	G	12	5	This week	2
Short Musculoskeletal Function Assessment Questionnaire (Function index)	SMFA	Swiontkowski MF. 1999	95	G	34	5	This week	2

Short Form of The Valued Life Activities Disability Questionnaire (Total)	S-VLA	Katz PP. 2011	96	D	14	5	Not specified	1					
Valued Life Activities Scale (Total)	VLA	Katz PP. 2006	97	D	33	4	Not specified	2					
World Health Organisation Disability Assessment Schedule –II (Total)	WHODAS-II	WHO 2004	53	G	36	5	Last 30 days	5					
World Health Organisation Disability Assessment Schedule –II (Understanding and communicating)	WHODAS-II	WHO 2004	53	G	6	5	Last 30 days	5					
World Health Organisation Quality of Life Instrument Short Form (Physical health)	WHOQoL-Bref	Harper H. 1998	40	D*	7	5	Past 4 weeks	15					

Evidence: Strong; Moderate; Weak; Absent

Supplementary Table 14: General characteristics and summary psychometric review of all versions of identified PROMs:
Environmental factors (e)

Name (Domain)	Acronym	First Author/ Year	REF (A)	Generic/ Disease Specific	N of items	Response Options	Recall period	Use	Psychometric Summary			
									Validity	Discrimination	Feasibility	Overall
Arthritis Impact Measurement Scales 2 (Support from family and friends)	AIMS2	Meenan RF. 1992	2	D	4	5	Past month	52				
Consumer Quality Index Rheumatoid Arthritis (Total)	CQ-Index RA	Zuidgeest M. 2009	98	D	142	2-4, 11	One year	1				
Internalised Stigma of Mental Illness-Rheumatoid Arthritis (Total)	ISMI-RA	Corker E. 2016	99	D	29	4	Now	2				
Person Centred Care in Outpatient Clinics/Rheumatol (Total)	PCCoc/rheum	Bala SV. 2018	100	D*	21	4	Not Specified	2				
Patient-Health Care Provider Communication Scale (Total)	PHCPCS	Salt E. 2013	101	D	21	4	Not specified	1				
World Health Organisation Quality of Life Instrument Short Form (Environment)	WHOQoL-Bref	Harper H. 1998	40	D*	8	5	Past 4 weeks	15				

Evidence: Strong; Moderate; Weak; Absent

Supplementary Table 15: General characteristics and summary psychometric review of all versions of identified PROMs:
Personal factors (e.g. self-efficacy, coping)

Name (Domain)	Acronym	First Author/ Year	REF (A)	Generic/ Disease Specific	N of items	Response Options	Recall period	Use	Psychometric Summary			
									Validity	Discrimination	Feasibility	Overall
Arthritis Helplessness Index (Total)	AHI	Nicassio PM. 1985	102	D	15	4	Present	8				
Arthritis Self-Efficacy Scale (Self-efficacy - other symptoms)	ASES	Lorig K. 1989	103	G	6	10	At the moment	25				
Arthritis Self-Efficacy Scale (Self-efficacy - pain)	ASES	Lorig K. 1989	103	G	5	10	At the moment	25				
Arthritis Self-Efficacy Scale (Self-efficacy - function)	ASES	Lorig K. 1989	103	G	9	10	At the moment	25				
Arthritis Self-Efficacy Scale-8 (Total)	ASES-8	Gonzalez VM. 1995	104	D*	8	10	At the moment	4				
Brief Resilient Coping Scale (Total)	BRCS	Sinclair VG. 2004	105	D	4	5	Not specified	2				
Exercise Self-Efficacy Scale (Total)	ESES	Dzewaltowski D. 1989	106	D*	6	10	At present	2				
Joint Protection Self-Efficacy Scale (Total)	JP-SES	Niedermann K. 2011	107	D	10	4	At the moment	3				
Rheumatoid Arthritis Empowerment Scale (Total)	RAEH	Contreras-Yáñez I. 2018	108	D*	8	5	Currently	1				
Rheumatology Attitudes Index (Total)	RAI	Callahan LF. 1988	109	D	15	5	Not specified	4				
Rheumatoid Arthritis Self Efficacy Scale (Total)	RASE	Hewlett S. 2001	110	D	28	5	At the moment	11				

Risk Perception Questionnaire (Total)	RPQ	Contreras-Yáñez I. 2019	111	D	27	11	Currently	1					
Self-Care Behaviours Scale (Total)	SCBS	Nadrian H. 2019	112	D	25	5	Past year	1					
Self-Injection Assessment Questionnaire (PRE Module)	SIAQ	Keininger D. 2011	113	D*	7	5	At the moment	3					
Self-Injection Assessment Questionnaire (POST module)	SIAQ	Keininger D. 2011	113	D*	21	5	At the moment	3					
Vanderbilt Pain Management Inventory (Active coping)	VPMI	Brown GK. 1987	114	D	7	4	Not specified	2					
Vanderbilt Pain Management Inventory (Passive coping)	VPMI	Brown GK. 1987	114	D	11	4	Not specified	2					

Evidence: Strong; Moderate; Weak; Absent

Supplementary Table 16: General characteristics and summary psychometric review of all versions of identified PROMs:
Education (knowledge and needs)

Name (Domain)	Acronym	First Author/ Year	REF (A)	Generic/ Disease Specific	N of items	Response Options	Recall period	Use	Psychometric Summary			
									Validity	Discrimination	Feasibility	Overall
Educational Needs Assessment Tool (Arthritis process)	ENAT	Hardware B. 2004	115	D*	7	5	At present	11				
Educational Needs Assessment Tool (Feelings)	ENAT	Hardware B. 2004	115	D*	4	5	At present	11				
Educational Needs Assessment Tool (Movement)	ENAT	Hardware B. 2004	115	D*	5	5	At present	11				
Educational Needs Assessment Tool (Managing pain)	ENAT	Hardware B. 2004	115	D*	6	5	At present	11				
Educational Needs Assessment Tool (Self-help measures)	ENAT	Hardware B. 2004	115	D*	6	5	At present	11				
Educational Needs Assessment Tool (Support systems)	ENAT	Hardware B. 2004	115	D*	4	5	At present	11				
Educational Needs Assessment Tool (Total)	ENAT	Hardware B. 2004	115	D*	39	5	At present	11				
Educational Needs Assessment Tool (Treatments)	ENAT	Hardware B. 2004	115	D*	7	5	At present	11				
Heart Disease Fact Questionnaire- Rheumatoid Arthritis (Total)	HDFQ-RA	John H. 2009	116	D	13	2	At present	2				
RA Knowledge Assessment Scale (Total)	RA-KAS	Naqvi AA. 2019	117	D	13	2-5	Current	1				

Evidence: Strong; Moderate; Weak; Absent

Supplementary Table 17: General characteristics and summary psychometric review of all versions of identified PROMs:
Quality of life, including well-being, and general health

Name (Domain)	Acronym	First Author/ Year	REF (A)	Generic/ Disease Specific	N of items	Response Options	Recall period	Use	Psychometric Summary			
									Validity	Discrimination	Feasibility	Overall
Foot Health Status Questionnaire (Footwear)	FHSQ	Bennett PJ. 1998	5	G	3	5	Past week	4				
Foot Health Status Questionnaire (General foot health)	FHSQ	Bennett PJ. 1998	5	G	2	5	Past week	4				
Michigan Hand Outcomes Questionnaire (Patient satisfaction with hand function)	MHQ	Chung KC. 1998	6	D*	6	5	Past week	37				
Patient-Reported Outcomes Measurement Information System Global Heath SF1.1 (Total)	PROMIS-GH v1.1-SF	Hays RD. 2009	118	G	10	5/11	In general / Past 7 days	1				
Quality of Life-Rheumatoid Arthritis Scale (Total)	QoL-RA	Danao LL. 2001	119	D	8	10	Not specified	6				
RAND 36-Item Health Survey (General Health)	RAND-36	Hays RD. 1993	13	G	5	5	Past 4 weeks	20				
Rheumatoid and Arthritis Outcome Score (QoL)	RAOS	Bremander AB. 2003	14	D	4	5	Last week	6				
Rheumatoid Arthritis Quality of Life Scale (Total)	RAQoL	De Jong Z. 1997	120	D	30	2	At the moment	58				
Self-Administered Foot Evaluation Questionnaire (General Health and Well-Being)	SAFE-Q	Niki H. 2011	17	D*	5	5	Past week	8				

Medical Outcomes Study Short Form 36-Item (General Health)	SF-36	Ware JE. 1992	18	D*	5	5	Past 4 weeks	562					

Evidence: Strong; Moderate; Weak; Absent

Supplementary Table 18: General characteristics and summary psychometric review of all versions of identified PROMs:
Health utilities

Name (Domain)	Acronym	First Author/ Year	REF (A)	Generic/ Disease Specific	N of items	Response Options	Recall period	Use	Psychometric Summary			
									Validity	Discrimination	Feasibility	Overall
EuroQol (Utility)	EQ-5D	Hurst N. 1994	121	D*	5	3, 5	Today	239				
Health Utilities Index-2 (Index)	HUI-2	Torrance GW. 1996	122	G	7	3-5	1/2/4 weeks or usual	6				
Health Utilities Index-3 (Index)	HUI-3	Feeny D. 2002	123	G	8	5-6	1/2/4 weeks or usual	6				
Quality of Well-Being Scale (Index)	QWB	Kaplan RM. 1997	124	G	74	2-4	Three days	3				
Medical Outcomes Study Short Form 6D (Total)	SF-6D	Brazier J. 2002	125	D*	11	4-6	1 week or 4 weeks	54				

Evidence: Strong; Moderate; Weak; Absent
