# Supplementary material for: Use and detailed metric properties of patient-reported outcome measures for rheumatoid arthritis: a systematic review covering two decades
Source: RMD Open. 2021 Aug 10;7(2):e001707. doi: 10.1136/rmdopen-2021-001707 (PMC8356163; doi:10.1136/rmdopen-2021-001707)
Supplement: Supplementary data [file rmdopen-2021-001707supp003.pdf]

Supplementary file 3

PROM specific references

1. Meenan RF, Gertman PM, Mason JH. Measuring health status in arthritis, Arthritis & Rheumatism, 1980;23(2):146-52.

2. Meenan RF, Mason JH, Anderson JJ, Guccione AA, Kazis LE. AIMS2, The content and properties of a revised and expanded Arthritis Impact Measurement Scales Health Status Questionnaire, Arthritis & Rheumatism, 1992;35(1):1-10.

3. Bellamy N, Campbell J, Haraoui B, Buchbinder R, Hobby K, Roth JH, MacDermid JC. Dimensionality and clinical importance of pain and disability in hand osteoarthritis: Development of the Australian/Canadian (AUSCAN) Osteoarthritis Hand Index, Osteoarthritis and Cartilage, 2002;10(11):855-62.

4. Budiman-Mak E, Conrad KJ, Roach KE. The Foot Function Index: a measure of foot pain and disability, Journal of clinical epidemiology, 1991;44(6):561-70.

5. Bennett PJ, Patterson C. The foot health status questionnaire (FHSQ): a new instrument for measuring outcomes of foot care, Australasian J Podiatr Med 1998;32:55-9.

6. Chung KC, Pillsbury MS, Walters MR, Hayward RA. Reliability and validity testing of the Michigan Hand Outcomes Questionnaire, The Journal of hand surgery, 1998;23(4):575-87.

7. Gonzalez VM, Stewart A, Ritter PL, Lorig K. Translation and validation of arthritis outcome measures into Spanish. Arthritis Rheum. 1995 Oct;38(10):1429-46.

8. Hunt SM, McEwen J, McKenna SP. Measuring health status: A new tool for clinicians and epidemiologists, The Journal of the Royal College of General Practitioners 1985; 35 (273): 185-8.

9. Dawson J, Doll H, Boller I, Fitzpatrick R, Little C, Rees J, Jenkinson C, Carr AJ. The development and validation of a patient-reported questionnaire to assess outcomes of elbow surgery; J Bone Joint Surg, [Br] (2008) 90-B: 466-473.

10. Freynhagen R, Baron R, Gockel U, Tolle TR. painDETECT: a new screening questionnaire to identify neuropathic components in patients with back pain, Curr Med Res Opin, 2006;22(10):1911-20.

11. Hinchcliff M, Beaumont JL, Thavarajah K, Varga J, Chung A, Podlusky S, Carns M, Chang RW, Cella D. Validity of two new patient-reported outcome measures in systemic sclerosis: Patient-Reported Outcomes Measurement Information System 29-item Health Profile and Functional Assessment of Chronic Illness Therapy Dyspnea short form, Arthritis Care Res (Hoboken), 2011; 63(11):1620-1628.

12. Stone AA, Broderick JE, Junghaenel DU, Schneider S, Schwartz JE. PROMIS fatigue, pain intensity, pain interference, pain behavior, physical function, depression, anxiety, and anger scales demonstrate ecological validity. J Clin Epidemiol 2016;74:194-206.

13. Hays RD, Sherborne CD, Mazel RM. The RAND 36-Item Health Survey 1,0, Health Econ 1993: 2:217-27.

14. Bremander AB, Petersson IF, Roos EM. Validation of the Rheumatoid and Arthritis Outcome Score (RAOS) for the lower extremity, Health Qual Life Outcomes, 2003 Oct 17;1:55.

15. Anderson DL. Development of an instrument to measure pain in rheumatoid arthritis: Rheumatoid Arthritis Pain Scale (RAPS), Arthritis Rheum, 2001 Aug;45(4):317-23.

16. Leeb B, Sautner J, Andel I, Rintelen B. SACRAH: a score for assessment and quantification of chronic rheumatic affections of the hands, Rheumatology, 2003;42(10):1173-8.

17. Niki H, Tatsunami S, Haraguchi N, Aoki T, Okuda R, Suda Y, Takao M, Tanaka Y. Development of the patient-based outcome instrument for foot and ankle: part 2: results from the second field survey: validity of the Outcome Instrument for the foot and ankle version 2, J Orthop Sci, 2011 Sep;16(5):556-64.

18. Ware JE, Sherbourne CD. The MOS 36-item short-form health survey (SF-36): I, Conceptual framework and item selection, Medical care, 1992:473-83.

19. Melzack R. The short-form McGill Pain Questionnaire, Pain 1987;30: 191-7.

20. Bellamy N. Validation study of WOMAC: a health status instrument for measuring clinically-important patient-relevant outcomes following total hip or knee arthroplasty in osteoarthritis, J Orthop Rheumatol, 1988;1:95-108.

21. Nicklin J, Cramp F, Kirwan J, Greenwood R, Urban M, Hewlett S. Measuring fatigue in RA: a cross-sectional study to evaluate the Bristol Rheumatoid Arthritis Fatigue Multi-Dimensional Questionnaire, Visual Analog Scales, and Numerical Rating Scales, Arthritis Care Res 2010;62:1559-68.

22. Chalder T, Berelowitz G, Pawlikowska T, Watts L, Wessely S, Wright D, Wallace EP. Development of a fatigue scale, J Psychosom Res 1993;37:147-53.

23. Vercoulen J, Swanink C, Fennis J, Galama J, van der Meer J, Bleijenberg G. Dimensional assessment of chronic fatigue syndrome, J Psychosom Res 1994;38:383-92.

24. Yellen SB, Cella DF, Webster K, Blendowski C, Kaplan E. Measuring fatigue and other anemia-related symptoms with the Functional Assessment of Cancer Therapy (FACT) Measurement System, J Pain Symptom Manage 1997;13:63-74.

25. Krupp LB, LaRocca NG, Muir-Nash J, Steinberg AD. The Fatigue Severity Scale: application to patients with multiple sclerosis and systemic lupus erythematosis, Arch Neurol 1989;46:1121-3.

26. Tack B. Dimensions and correlates of fatigue in older adults with rheumatoid arthritis, San Francisco: University of California; 1991.

27. Smets E, Garssen B, Bonke B, De Haes J. The Multidimensional Fatigue Inventory (MFI): psychometric qualities of an instrument to assess fatigue, J Psychosomatic Res 1995;39:315-25.

28. McNair DM, Lorr M, & Droppleman LF. Manual for the Profile of Mood States, San Diego, CA: Educational and Industrial Testing Services, 1971.

29. Bingham III CO, Gutierrez AK, Butanis A, Bykerk VP, Curtis JR, Leong A, Lyddiatt A, Nowell WB, Orbai AM, Bartlett S. PROMIS Fatigue short forms are reliable and valid in adults with rheumatoid arthritis. J Patient Rep Outcomes. 2019 Dec; 3:14.

30. Bingham III CO, Gutierrez AK, Butanis A, Bykerk VP, Curtis JR, Leong A, Lyddiatt A, Nowell WB, Orbai AM, Bartlett S. PROMIS Fatigue short forms are reliable and valid in adults with rheumatoid arthritis. J Patient Rep Outcomes. 2019 Dec; 3:14.

31. Bingham III CO, Gutierrez AK, Butanis A, Bykerk VP, Curtis JR, Leong A, Lyddiatt A, Nowell WB, Orbai AM, Bartlett S. PROMIS Fatigue short forms are reliable and valid in adults with rheumatoid arthritis. J Patient Rep Outcomes. 2019 Dec; 3:14.

32. Ryan RM, Frederick C. On energy, personality, and health: subjective vitality as a dynamic reflection of well-being, J Pers, 1997;65(3):529-565.

33. Guillemin F, Coste J, Pouchot J, Ghézail M, Bregeon C, Sany J. The AIMS2-SF, A short form of the arthritis impact measurement scales 2, Arthritis & Rheumatism, 1997;40(7):1267-74.

34. Radloff LS. The CES-D scale: a self-report depression scale for research in the general population, Applied Psychological Measurement 1977, 1:385-401.

35. Weisman MH, Paulus HD, Russak SM, Lubeck DP, Chiou CF, Sengupta N, Ofman JJ, Borenstein J, Moadel AB, Sherbourne CD. Development of a new instrument for rheumatoid arthritis (RA): the Cedars-Sinai Health-Related Quality of Life Instrument (CSHQ-RA), Arthritis Rheum (Arthritis Care Res) 2003;49:78-84.

36. Lovibond SH, Lovibond PF. Manual for the depression anxiety stress scales, 2 edition, Sydney: Psychology Foundation; 1995.

37. Sinclair VG, Dowdy SW. Development and validation of the Emotional Intimacy Scale, J Nurs Meas, 2005 Winter;13(3):193-206.

38. Zigmond AS, Snaith RP. The hospital anxiety and depression scale, Acta Psychiatr Scand, 1983;67(6):361-70,

39. Sinclair VG, Wallston KA. The Development and Validation of the Psychological Vulnerability Scale, Cognitive Therapy and Research 1999;23(2):119-129.

40. Harper H, Power M, Group TW. Development of the World Health Organization WHOQOL-BREF quality of life assessment, Psychol Med 1998;28:551-8.

41. Soldatos CR, Dikeos DG, Paparrigopoulos TJ. Athens Insomnia Scale: Validation of an instrument based on ICD-10 criteria, J Psychosom Res 2000;48:555-60.

42. Jenkins DC, Stanton B, Niemcryk SJ, Rose RM. A scale for the estimation of sleep problems in clinical research. J Clin Epidemiol 1988;41(4):313-321.

43. Hays RD, Stewart AL. Measuring function and well being, The medical outcomes study approach sleep measures, Durham North Carolina, USA: Duke University Press 1992:235-259.

44. Williams B, Dissertation Advisor Bartels J. Coventry (UK): Coventry University; 2001, The body experience in the context of arthritis: a psychosocial perspective [dissertation].

45. Helliwell P, Reay N, Gilworth G, Redmond A, Slade A, Tennant A, Woodburn J. Development of a foot impact scale for rheumatoid arthritis, Arthritis Rheum 2005;53:418-422.

46. Sullivan JJ, Edgley K, Dehoux E. A survey of multiple sclerosis, Part 1, Perceived cognitive problems and compensatory strategy use, Can J Rehabil 1990;4:99-105.

47. Leeb BF, Haindl PM, Maktari A, Nothnagl T, Rintelen B. Patient-centered rheumatoid arthritis disease activity assessment by a modified RADAI. J Rheumatol. 2008;35(7):1294-9.

48. Banderas B, Skup M, Shields AL, Mazar I, Ganguli A. Development of the Rheumatoid Arthritis Symptom Questionnaire (RASQ): a patient reported outcome scale for measuring symptoms of rheumatoid arthritis, Current Medical Research and Opinion, 2017;33(9):1643-1651.

49. Ware JE, Kosinski M, Keller SD. A 12-Item Short-Form Health Survey: construction of scales and preliminary tests of reliability and validity, Medical care, 1996;34(3):220-33.

50. Nordenskiöld U, Grimby G, Hedberg M, Wright B, Linacre JM. The structure of an instrument for assessing the effects of assistive devices and altered working methods in women with rheumatoid arthritis, Arthritis Care Res, 1996;9(5):358-67.

51. Craig CL, Marshall AL, Sjöström M, Bauman AE, Booth ML, Ainsworth BE, Pratt M, Ekelund U, Yngve A, Sallis JF, Oja P. International Physical Activity Questionnaire: 12-country reli-ability and validity, Med Sci Sports Exerc 2003;35:1381-95.

52. Salaffi F, Bazzichi L, Stancati A, Neri R, Cazzato M, Consensi A, Grassi W, Bombardieri S. Development of a functional disability measurement tool to assess early arthritis: the Recent-Onset Arthritis Disability (ROAD) questionnaire, Clin Exp Rheumatol, 2005 Sep-Oct;23(5):628-36.

53. World Health Organization (WHO), WHODAS II - Disability Assessment Schedule Training Manual: A guide to administration [Internet], Geneve: WHO; 2004.

54. de Klerk E, van der Heijde D, van der Tempel H, van der Linden S. Development of a questionnaire to investigate patient compliance with antirheumatic drug therapy, J Rheumatol 1999;26:2635-2641.

55. Hughes LD, Done J, Young A. A 5 item version of the Compliance Questionnaire for Rheumatology (CQR5) successfully identifies low adherence to DMARDs. BMC Musculoskelet Disord. 2013;14:286.

56. Horne R, Weinman J. Self-regulation and self-management in asthma: exploring the role of illness perceptions and treatment beliefs in explaining non-adherence to preventer medication, Psychol Health 2002;17:17-32.

57. Salt E, Hall L, Peden AR, Home R. Psychometric properties of three medication adherence scales in patients with rheumatoid arthritis, J Nurs Meas, 2012;20(1):59-72,

58. Morisky DE, Green LW, Levine DM. Concurrent and predictive validity of a self-reported measure of medication adherence, Med Care 1986, 24(1):67-74.

59. Ende van den CHM, Rozing PM, Dijkmans BAC, Verhoef JAC, Voogt- van der Harst EM, Hazes JMW. Assessment of shoulder functions in rheumatoid arthritis, J Rheumatol 1996;23:2043-8.

60. Endicott J, Nee J. Endicott Work Productivity Scale (EWPS): a new measure to assess treatment effects, Psychopharmacol Bull 1997;33:13-16.

61. Gilworth G, Chamberlain MA, Harvey A, Woodhouse A, Smith J, Smyth MG, Tennant A. Development of a Work Instability Scale for Rheumatoid Arthritis, Arthritis & Rheumatism (Arthritis Care & Research) 2003;49(3):349-354.

62. Koopman C, Pelletier KR, Murray JF, Sharda CE, Berger ML, Turpin RS, Hackleman P, Gibson P, Holmes DM, Bendel T. Stanford Presenteeism Scale: health status and employee productivity, J Occup Environ Med 2002;44:14-20.

63. Zhang W, Bansback N, Kopec J, Anis AH. Measuring time input loss among patients with rheumatoid arthritis: validity and reliability of the Valuation of Lost Productivity questionnaire, J Occup Environ Med, 2011 May;53(5):530-6.

64. Gignac MA, Badley EM, Lacaille D, Cott CC, Adam P, Anis AH. Managing arthritis and employment: making arthritis-related work changes as a means of adaptation, Arthritis Rheum 2004;51:909-16.

65. Fujino Y, Uehara M, Izumi H, Nagata T, Muramatsu K, Kubo T, Oyama I, Matsuda S. Development and validity of a work functioning impairment scale based on the Rasch model among Japanese workers. J Occup Health. 2015;57(6):521-31.

66. Lerner D, Amick III BC, Rogers WH, Malspeis S, Bungay K, Cynn D. The work limitations questionnaire, Medical care, 2001;39(1):72-85.

67. Penta M, Thonnard JL, Tesio L. ABILHAND: a Rasch-built measure of manual ability, Arch Phys Med Rehabil, 1998 Sep;79(9):1038-42.

68. Duruöz M, Poiraudeau S, Fermanian J, Menkes C, Amor B, Dougados M, Revel M. Development and validation of a rheumatoid hand functional disability scale that assesses functional handicap, The Journal of rheumatology, 1996;23(7):1167-72.

69. Hudak PL, Amadio PC, Bombardier C. Development of an upper extremity outcome measure: the DASH (Disabilities of the Arm, Shoulder and Hand), Am J Ind Med 1996;29:602-8.

70. Budiman-Mak E, Conrad K, Stuck R, Matters M. Theoretical model and Rasch analysis to develop a revised Foot Function Index. Foot Ankle Int. 2006 Jul;27(7):519-27.

71. Fries JF, Spitz P, Kraines RG, Holman HR. Measurement of patient outcome in arthritis, Arthritis & Rheumatism, 1980;23(2):137-45.

72. Wolfe F, Michaud K, Pincus T. Development and validation of the health assessment questionnaire II: a revised version of the health assessment questionnaire, Arthritis Rheum 2004;50:3296-305.

73. Hammond A, Lincoln N. Development of the joint Protection behavior assessment, Arthritis Care Res 1999;12(3):200-207.

74. Tugwell P, Bombardier C, Buchanan WW, Goldsmith C, Grace E & Hanna B. The MACTAR patient preference disability questionnaire - An individualized functional priority approach for assessing improvement in physical disability in clinical trials in rheumatoid arthritis, J Rheumatol 1987;14:446-451..

75. Goodacre L, Smith J, Meddis D, Goodacre J. Development and validation of a patient-centred Measure of Activity Limitation (MAL) in rheumatoid arthritis, Rheumatology 2007;46:703-708.

76. Paulsen T, Grotle M, Garratt A, Kjeken I. Development and psychometric testing of the patient-reported measure of activity performance of the hand (MAP-Hand) in rheumatoid arthritis, J Rehabil Med, 2010 Jul;42(7):636-44.

77. Pincus T, Swearingen C, Wolfe F. Toward a multidimensional Health Assessment Questionnaire (MDHAQ): assessment of advanced activities of daily living and psychological status in the patient-friendly health assessment questionnaire format, Arthritis Rheum 1999;42:2220-30.

78. Pincus T, Summey JA, Soraci SA, Wallston KA, Hummon NP. Assessment of patient satisfaction in activities of daily living using a modified stanford health assessment questionnaire, Arthritis and Rheumatism 1983; 26: 1346-53,

79. Hewlett S, Smith A, and Kirwan J. Measuring the meaning of disability in rheumatoid arthritis: the Personal Impact Health Assessment Questionnaire (PI HAQ), Ann Rheum Dis, 2002 Nov; 61(11): 986-993.

80. Fries JF, Cella D, Rose M, Krishnan E, Bruce B. Progress in assessing physical function in arthritis: PROMIS short forms and computerized adaptive testing, J Rheumatol, 2009;36(9):2061-2066.

81. Fries JF, Cella D, Rose M, Krishnan E, Bruce B. Progress in assessing physical function in arthritis: PROMIS short forms and computerized adaptive testing, J Rheumatol, 2009;36(9):2061-2066.

82. Archenholtz B, Dellhag B. Validity and reliability of the instrument Performance and Satisfaction in Activities of Daily Living (PS-ADL) and its clinical applicability to adults with rheumatoid arthritis, Scand J Occup Ther, 2008 Mar;15(1):13-22.

83. Hammond A, Prior Y, Tyson S. Linguistic Validation, Validity and Reliability of the British English Versions of the Disabilities of the Arm, Shoulder and Hand (DASH) Questionnaire and QuickDASH in People With Rheumatoid Arthritis. BMC Musculoskelet Disord 2018;19(1):118.

84. Guermazi M, Kessomtini W, Poiraudeau S, Elleuch M, Fermarian J, Elleuch MH, Revel M. Development and validation of an Arabic rheumatoid hand disability scale, Disabil Rehabil, 2004 Jun 3;26(11):655-61.

85. Wikström I, Arvidsson B, Nilsson K, Roos E, Jacobsson LT. Reliability, validity and responsiveness of a new leisure index: the Patient-Specific Leisure Scale (PSLS), Musculoskeletal Care, 2009 Sep;7(3):178-93.

86. Salaffi F, Di Carlo M, Carotti M, Farah S. The Patient-Reported Outcomes Thermometer-5-Item Scale (5T-PROs): Validation of a New Tool for the Quick Assessment of Overall Health Status in Painful Rheumatic Diseases. Pain Res Manag 2018; 2018:3496846.

87. Chiou CF, Sherbourne CD, Ofman J, Lee M, Lubeck DP, Paulus HE, Weisman M. Development and validation of Cedars-Sinai Health-Related Quality of Life in Rheumatoid Arthritis (CSHQ-RA) short form instrument, Arthritis Rheum, 2004 Jun 15;51(3):358-64.

88. Berthelot JM, De Bandt M, Morel J, Benatig F, Constantin A, Gaudin P, Le Loet X, Maillefert JF, Meyer O, Pham T, Saraux A, Solau-Gervais E, Spitz E, Wendling D, Fautrel B, Guillemin F. A tool to identify recent or present rheumatoid arthritis fl are from both patient and physician perspectives: The ‘FLARE’ instrument, Ann Rheum Dis 2012;71:1110-1116.

89. Sautner J, Andel I, Rintelen B, Leeb BF. Development of the M-SACRAH, a modified, shortened version of SACRAH (Score for the Assessment and Quantification of Chronic Rheumatoid Affections of the Hands), Rheumatology (Oxford), 2004 Nov;43(11):1409-13.

90. Hill JC, Kang S, Benedetto E et al. Development and initial cohort validation of the Arthritis Research UK Musculoskeletal Health Questionnaire (MSK-HQ) for use across musculoskeletal care pathways. BMJ Open 2016;6:e012331.

91. Bartlett SJ, Barbic SP, Bykerk VP, Choy EH, Alten R, Christensen R, den Broeder A, Fautrel B, Furst DE, Guillemin F, Hewlett S, Leong AL, Lyddiatt AL, March L, Montie P, Pohl C, Voshaar MS, Woodworth TG, Bingham III CO. Content and Construct Validity, Reliability, and Responsiveness of the Rheumatoid Arthritis Flare Questionnaire: OMERACT 2016 Workshop Report, J Rheumatol, 2017;44:1536-1543.

92. Gossec L, Dougados M, Rincheval N, Balanescu A, Boumpas DT, Canadelo S, Carmona L, Daurès JP, de Wit M, Dijkmans BA, Englbrecht M, Gunendi Z, Heiberg T, Kirwan JR, Mola EM, Matucci-Cerinic M, Otsa K, Schett G, Sokka T, Wells GA, Aanerud GJ, Celano A, Dudkin A, Hernandez C, Koutsogianni K, Akca FN, Petre AM, Richards P, Scholte-Voshaar M, Von Krause G, Kvien TK. Elaboration of the preliminary Rheumatoid Arthritis Impact of Disease (RAID) score: a EULAR initiative, Ann Rheum Dis, 2009 Nov;68(11):1680-5.

93. Pincus T, Yazici Y, Bergman MJ. RAPID3, an index to assess and monitor patients with rheumatoid arthritis, without formal joint counts: similar results to DAS28 and CDAI in clinical trials and clinical care. Rheum Dis Clin North Am 2009;35(4):773-778.

94. Rintelen B, Haindl PM, Mai HT, Sautner J, Maktari A, Leeb BF. A tool for the assessment of hand involvement in rheumatic disorders in daily routine--the SF-SACRAH (short form score for the assessment and quantification of chronic rheumatic affections of the hands), Osteoarthritis Cartilage, 2009 Jan;17(1):59-63.

95. Swiontkowski MF, Engelberg R, Martin DP, Agel J. Short musculoskeletal function assessment questionnaire: Reliability, validity and responsiveness, J Bone Joint Surg [Am] 1999; 81a: 1245-1260.

96. Katz PP, Radvanski DC, Allen D, Buyske S, Schiff S, Nadkarni A, Rosenblatt L, Maclean R, Hassett AL. Development and Validation of a Short Form of the Valued Life Activities Disability Questionnaire for Rheumatoid Arthritis, Arthritis Care Res (Hoboken), 2011 Dec; 63(12): 1664-1671.

97. Katz PP, Morris A and Yelin EH. Prevalence and predictors of disability in valued life activities among individuals with rheumatoid arthritis, Ann Rheum Dis 2006; 65:763-769.

98. Zuidgeest M, Sixma H, Rademakers J. Measuring patients' experiences with rheumatic care: the consumer quality index rheumatoid arthritis, Rheumatology International: 2009, 30(2), 159-167.

99. Corker E, Henderson RC, Lempp H, Brown JS. Internalised stigma in people with rheumatoid arthritis: a cross sectional study to establish the psychometric properties of the ISMI-RA, BMC Musculoskelet Disord, 2016;17:244.

100. Bala SV, Forslind K, Fridlund B, Samuelson K, Svensson B, Hagell P. Person-centred care in nurse-led outpatient rheumatology clinics: Conceptualization and initial development of a measurement instrument. Musculoskeletal Care. 2018;16(2):287-295.

101. Salt E, Crofford LJ, Studts JL, Lightfoot R, Hall LA. Development of a quality of patient-health care provider communication scale from the perspective of patients with rheumatoid arthritis, Chronic Illn, 2013 Jun;9(2):103-15.

102. Nicassio PM, Wallston KA, Callahan LF, Herbert M, Pincus P. The measurement of helplessness in rheumatoid arthritis: the development of the Arthritis Helplessness Index, J Rheumatol 1985;12:462-7.

103. Lorig K, Chastain RL, Ung E, Shoor S, Holman HR. Development and evaluation of a scale to measure perceived self-efficacy in people with arthritis, Arthritis Rheum 1989;32:37-44.

104. Gonzalez VM, Stewart A, Ritter PL, Lorig K. Translation and validation of arthritis outcome measures into Spanish, Arthritis Rheum 1995;38(10):1429-1446.

105. Sinclair VG, Wallston KA. The development and psychometric evaluation of the Brief Resilient Coping Scale, Assessment, 2004 Mar;11(1):94-101.

106. Dzewaltowski D. Toward a model of exercise motivation, J Sport Exerc Psychol 1989;11:251-269.

107. Niedermann K, Forster A, Ciurea A, Hammond A, Uebelhart D, de Bie R. Development and psychometric properties of a joint protection self-efficacy scale, Scand J Occup Ther, 2011 Jun;18(2):143-52,

108. Contreras-Yáñez I, Ruiz-Medrano E, Hernández LDCR, Pascual-Ramos V. Psychometric Validation of an Empowerment Scale for Spanish-speaking Patients With Rheumatoid Arthritis. Arthritis Res Ther 2018; 20(1):244.

109. Callahan LF, Brooks RH, Pincus T, (1988). Further analysis of learned helplessness in rheumatoid arthritis using a 'Rheumatology Attitudes Index', Journal of Rheumatology, 15(3), 418-426.

110. Hewlett S, Cockshott Z, Kirwan J, Barrett J, Stamp J, Haslock I. Development and validation of a self-efficacy scale for use in British patients with rheumatoid arthritis, Rheumatology (Oxford) 2001; 40: 1221-30.

111. Contreras-Yáñez I, Lavielle P, Clark P, Pascual-Ramos V. Validation of a risk perception questionnaire developed for patients with rheumatoid arthritis. PLoS One 2019;14:7.

112. Nadrian H, Hosseini Niaz Y, Basiri Z, Tahamoli Roudsari A. Development and psychometric properties of a self-care behaviors scale (SCBS) among patients with rheumatoid arthritis [published correction appears in BMC Rheumatol. 2019 Aug 20;3:35]. BMC Rheumatol. 2019;3:4.

113. Keininger D, Coteur G. Assessment of self-injection experience in patients with rheumatoid arthritis: psychometric validation of the Self-Injection Assessment Questionnaire (SIAQ), Health and Quality of Life Outcomes 2011, 9:2.

114. Brown GK, Nicassio PM. Development of a questionnaire for the assessment of active and passive coping strategies in chronic pain patients, Pain, 1987 Oct;31(1):53-64.

115. Hardware B, Anne Lacey E, & Shewan J. Towards the development of a tool to assess educational needs in patients with arthritis, Clinical Effectiveness in Nursing 2004;8(2),111-117.

116. John H, Treharne GJ, Hale ED, Panoulas VF, Carroll D, Kitas GD. Development and initial validation of a heart disease knowledge questionnaire for people with rheumatoid arthritis, Patient Educ Couns, 2009 Oct;77(1):136-43.

117. Naqvi AA, Hassali MA, Iffat W, Zia M, Fatima M, Shakeel S, Khan I, Jahangir A, Kachela B, Nadir MN, Qureshi I, Bangash U. Development and validation of a novel rheumatoid arthritis knowledge assessment scale in Pakistani patients with rheumatoid arthritis. Int J Rheum Dis. 2019;22(11):2031-2044.

118. Hays RD, Bjorner JB, Revicki DA, Spritzer KL, Cella D. Development of physical and mental health summary scores from the patient-reported outcomes measurement information system (PROMIS) global items. Qual Life Res. 2009 Sep;18(7):873-80.

119. Danao LL, Padilla GV, Johnson DA. An English and Spanish quality of life measure for rheumatoid arthritis, Arthritis Rheum, 2001 Apr;45(2):167-73.

120. De Jong Z, Van Der Heijde D, McKenna SP, Whalley D. The Reliability and Construct Validity of The RAQoL: A Rheumatoid Arthritis Specific Quality Of Life Instrument, British Journal of Rheumatology 1997; 36: 878-883.

121. Hurst N, Jobanputra P, Hunter M, Lambert M, Lochhead A. Brown H, Validity of euroqol—a generic health status instrument—in patients with rheumatoid arthritis economic and health outcomes research group, Rheumatology, 1994;33(7):655-62.

122. Torrance GW, Feeny DH, Furlong WJ, Barr RD, Zhang Y, Wang Q. Multi-Attribute Preference Functions for A Comprehensive Health Status Classification System, Health Utilities Index Mark 2, Med Care, 1996 Jul;34(7):702-22.

123. Feeny D, Furlong W, Torrance GW, Goldsmith CH, Zhu Z, DePauw S, Denton M, Boyle M. Multi-attribute and single-attribute utility functions for the Health Utilities Index Mark 3 system, Med Care, 2002;40(2):113-28.

124. Kaplan RM, Sieber WJ, Ganiats TG. The Quality of Well Being scale: Comparison of the interviewer-administered version with a self-administered questionnaire, Psychology Health,1997;12:783-791.

125. Brazier J, Roberts J, Deverill M. The estimation of a preference-based measure of health from the SF-36, Journal of health economics, 2002;21(2):271-92.
