# Supplementary material for: Use and detailed metric properties of patient-reported outcome measures for rheumatoid arthritis: a systematic review covering two decades
Source: RMD Open. 2021 Aug 10;7(2):e001707. doi: 10.1136/rmdopen-2021-001707 (PMC8356163; doi:10.1136/rmdopen-2021-001707)
Supplement: Supplementary data [file rmdopen-2021-001707supp004.pdf]

#	Scale Acronym	Authors	Title	Journal	Year	Volume	Page
1	**AIMS**	Abello-Banfi M, et al.	Quality of life in rheumatoid arthritis: Validation of a Spanish version of the Arthritis Impact Measurement Scales (Spanish-AIMS)	J Rheumatol	1994	21(7)	250-1255
		Archenholtz B & Bjelle A	Evaluation of a Swedish Version of the Arthritis Impact Measurement Scales (AIMS)	Scand J Rheumatol	1995	24(2)	64-68
		Buchbinder R, et al.	Which outcome measures should be used in rheumatoid arthritis clinical trials? Clinical and quality-of-life measures' responsiveness to treatment in a randomized controlled trial	Arthritis Rheum	1995	38(11)	568-1580
		Coulton CJ, et al.	An assessment of the Arthritis Impact Measurement Scales in 3 ethnic groups	J Rheumatol	1989	16(8)	110-1115
		Englbrecht M, et al.	Measuring pain and efficacy of pain treatment in inflammatory arthritis: a systematic literature review	J Rheumatol	2012	Suppl 90	3-10
		Fitzpatrick R, et al.	A generic health status instrument in the assessment of rheumatoid arthritis	Br J Rheumatol	1992	31(2)	87-90
		Fitzpatrick R, et al.	Importance of sensitivity to change as a criterion for selecting health status measures	Qual Health Care	1992	1(2)	89-93
		Hendricson WD, et al.	Development and initial validation of a dual-language English-Spanish format for the Arthritis Impact Measurement Scales	Arthritis Rheum	1989	32(9)	153-1159
		Hill J, et al.	The arthritis impact measurement scales: an anglicized version to assess the outcome of British patients with rheumatoid arthritis	Br J Rheumatol	1990	29(3)	193-196
		Kazis LE, et al.	Effect Sizes for Interpreting Changes in Health Status	Med Care	1989	27(3)	178-S189
		Lorish CD, et al.	A Comparison of the Full and Short Versions of the Arthritis Impact Measurement Scales	Arthritis Care Res	1991	4(4)	168-173
		Potts MK & Brandt KD	Evidence of the validity of the Arthritis Impact Measurement Scales	Arthritis Rheum	1987	30(1)	93-96
		Senerdem N, et al.	The Use of Two Different Health Assessment Questionnaires in Turkish Rheumatoid Arthritis Population and Assessment of the Associations with Disability	Clin Rheumatol	1999	18(1)	33-37
		Spitz PW & Fries JF	The present and future of comprehensive outcome measures for rheumatic diseases	Clin Rheumatol	1987	6.Suppl 2	105-111
		Taal E, et al.	Evaluation of the Dutch Arthritis Impact Measurement Scales (DUTCH-AIMS) in patients with rheumatoid arthritis	Br J Rheumatol	1989	28(6)	487-491
2	**AIMS2**	Archenholtz B & Bjelle A	Reliability, validity, and sensitivity of a Swedish version of the revised and expanded arthritis impact measurement scales (AIMS2)	J Rheumatol	1997	24(7)	370-1377
		Arkela-Kautiainen M, et al.	Evaluation of the Arthritis Impact Measurement Scales (AIMS2) in Finnish patients with rheumatoid arthritis	Scand J Rheumatol	2003	32 (5)	300-305
		Brandão L, et al.	Health status in rheumatoid arthritis: Cross cultural evaluation of a Portuguese version of the arthritis impact measurement scales 2 (BRASIL- AIMS2)	J Rheumatol	1998	25(8)	499-1501
		Chu EM, et al.	Translation and Validation of Arthritis Impact Measurement Scales 2 Into Chinese: CAIMS2	Arthritis Rheum	2004	51(1)	20-27
		da Mota Falcão D, et al.	Translation and cultural adaptation of quality of life questionnaires: an evaluation of methodology	J Rheumatol	2003	30(2)	379-385
		Englbrecht M, et al.	Measuring pain and efficacy of pain treatment in inflammatory arthritis: a systematic literature review	J Rheumatol	2012	Suppl 90	3-10
		Evers AW, et al.	A comparison of two recently developed health status instruments for patients with arthritis: Dutch-AIMS2 and IRGL	Br J Rheumatol	1998	37(2)	157-164
		Haavardsholm EA, et al.	A comparison of agreement and sensitivity to change between AIMS2 and a short form of AIMS2 (AIMS2-SF) in more than 1,000 rheumatoid arthritis patients	J Rheumatol	2000	27(12)	810-2816
		Hagen KB, et al.	The responsiveness of health status measures in patients with rheumatoid arthritis: Comparison of disease-specific and generic instruments	J Rheumatol	1999	26(7)	474-1480
		Kvien TK, et al.	Performance of the Norwegian SF-36 Health Survey in Patients with Rheumatoid Arthritis. II. A Comparison of the SF-36 with Disease-Specific Measures	J Clin Epidemiol	1998	51(11)	077-1086
		Mason JH, et al.	Do Self-Reported Arthritis Symptom (IRADAR) and Health Status (AIMS2) Data Provide Duplicative or Colmplementary Information?	Arthritis Care Res	1992	5(3)	163-170
		Meenan RF, et al.	AIMS2: The Content and Properties of a Revised and Expanded Arthritis Impact Measurement Scales Health Status Questionnaire	Arthritis Rheum	1992	35(1)	1-10
		Pouchot J, et al.	Validation of the French version of the arthritis impact measurement scales 2 and comparison with the French version of the Nottingham health profile	Rev Rhum Engl Ed	1996	63(6)	389-404
		Riemsma RP, et al.	Evaluation of a Dutch version of the AIMS2 for patients with rheumatoid arthritis	Br J Rheumatol	1996	35	755-760
		Salaffi F, et al.	Responsiveness of Health Status Measures and Utility-based Methods in Patients with Rheumatoid Arthritis	Clin Rheumatol	2002	21(6)	478-487
		Sato H, et al.	Validity and reliability of a revised Japanese version of the Arthritis Impact Measurement Scales version 2 (AIMS2)	Mod Rheumatol	2000	10(4)	247-255
		Soosova & Macejova	Is the Arthritis Impact Measurement Scales 2 a good tool to assess quality of life in Slovak patients with rheumatoid arthritis?	Bratisl Lek Listy	2013	114(9)	534 - 539
		Taal E, et al.	Sensitivity to change of AIMS2 and AIMS2-SF components in comparison to M-HAQ and VAS-pain	Ann Rheum Dis	2004	63(12)	655-1658
		van der Giesen FJ, et al.	Responsiveness of the Michigan Hand Outcomes Questionnaire–Dutch Language Version in Patients With Rheumatoid Arthritis	Arch Phys Med Rehabil	2008	89(6)	121-1126
		Veehof MM, et al.	Comparison of Internal and External Responsiveness of the Generic Medical Outcome Study Short Form-36 (SF-36) with Disease-specific Measures in Rheumatoid Arthritis	J Rheumatol	2008	35(4)	610-617
		Waljee JF, et al.	Validity and Responsiveness of the Michigan Hand Questionnaire in Patients With Rheumatoid Arthritis: A Multicenter, International Study	Arthritis Care Res	2010	62(11)	569-1577
3	**AUSCAN**	Massy-Westropp N, et al.	Comparing the AUSCAN Osteoarthritis Hand Index, Michigan Hand Outcomes Questionnaire, and Sequential Occupational Dexterity Assessment for patients with rheumatoid arthritis	J Rheumatol	2004	31(10)	996-2001
4	**FFI**	Budiman-Mak E, et al.	The Foot Function Index: a measure of foot pain and disability	J Clin Epidemiol	1991	44(6)	561-570
		Muradin I & van der Heide HJ	The foot function index is more sensitive to change than the Leeds Foot Impact Scale for evaluating rheumatoid arthritis patients after forefoot or hindfoot reconstruction	Int Orthop	2016	40(4)	745-749
		Pourtier-Piotte C, et al.	French validation of the Foot Function Index (FFI)	Ann Phys Rehabil Med	2005	58	276-282
5	**FHSQ**	Ferreira AF, et al.	Brazilian version of the foot health status questionnaire (FHSQ-BR): cross-cultural adaptation and evaluation of measurement properties	Clinics	2008	63(5)	595-600
6	**MHQ**	Chung KC, et al.	Reliability and Validity Testing of the Michigan Hand Outcomes Questionnaire	J Hand Surg Am	1998	23(4)	575-587
		Dritsaki M, et al.	An empirical evaluation of the SF-12, SF-6D, EQ-5D and Michigan Hand Outcome Questionnaire in patients with rheumatoid arthritis of the hand	Health Qual Life Outcomes	2017	15(1)	20
		Massy-Westropp N, et al.	Comparing the AUSCAN Osteoarthritis Hand Index, Michigan Hand Outcomes Questionnaire, and Sequential Occupational Dexterity Assessment for patients with rheumatoid arthritis	J Rheumatol	2004	31(10)	996-2001
		Meireles SM, et al.	Cross-cultural adaptation and validation of the Michigan Hand Outcomes Questionnaire (MHQ) for Brazil: validation study	Sao Paulo Med J	2014	132(6)	339-347
		Sears ED, et al.	Validity and Responsiveness of the Jebsen–Taylor Hand Function Test	J Hand Surg Am	2010	35(1)	30-37
		Shauver MJ & Chung KC	The Minimal Clinically Important Difference of the Michigan Hand Outcomes Questionnaire	J Hand Surg Am	2009	34(3)	509-514
		van der Giesen FJ, et al.	Responsiveness of the Michigan Hand Outcomes Questionnaire–Dutch Language Version in Patients With Rheumatoid Arthritis	Arch Phys Med Rehabil	2008	89(6)	121-1126
		Waljee JF, et al.	Validity and Responsiveness of the Michigan Hand Questionnaire in Patients With Rheumatoid Arthritis: A Multicenter, International Study	Arthritis Care Res	2010	62(11)	569-1577
		Yasui T, et al.	Relationship between roentgenographic joint destruction in the hands and functional disorders among patients with rheumatoid arthritis	Mod Rheumatol	2017	27(5)	828-832
7	**MOS-PI**	Horta-Baas G, et al.	Evaluation of pain intensity in people with rheumatoid arthritis using the MOS intensity scale	Med Clin (Barc)	2019	153(3)	106-111
8	**NHP**	Akar S, et al.	Quality of life in patients with Takayasu's arteritis is impaired and comparable with rheumatoid arthritis and ankylosing spondylitis patients	Clin Rheumatol	2008	27(7)	859-865
		Borman P, et al.	A comparative evaluation of quality of life and life satisfaction in patients with psoriatic and rheumatoid arthritis	Clin Rheumatol	2007	26(3)	330-334
		Eberhardt K, et al.	Measuring health related quality of life in patients with rheumatoid arthritis - reliability, validity, and responsiveness of a Swedish version of RAQoL	Scand J Rheumatol	2002	31(1)	6-12
		Fitzpatrick R, et al.	A generic health status instrument in the assessment of rheumatoid arthritis	Br J Rheumatol	1992	31(2)	87-90
		Fitzpatrick R, et al.	Importance of sensitivity to change as a criterion for selecting health status measures	Qual Health Care	1992	1(2)	89-93
		Houssien DA, et al.	The Nottingham health profile as a measure of disease activity and outcome in rheumatoid arthritis	Br J Rheumatol	1997	36(1)	69-73
		Jenkinson C, et al.	The Nottingham health profile: An analysis of its sensitivity in differentiating illness groups	Soc Sci Med	1988	27(12)	411-1414
		Lovas K, et al.	Establishing a standard for patient-completed instrument adaptations in Eastern Europe: experience with the Nottingham Health Profile in Hungary	Health Policy	2003	63(1)	49-61
		Pouchot J, et al.	Validation of the French version of the arthritis impact measurement scales 2 and comparison with the French version of the Nottingham health profile	Rev Rhum Engl Ed	1996	63(6)	389-404
		Ribas SA, et al.	Sensitivity and specificity of assessment instruments of quality of life in rheumatoid arthritis	Rev Bras Reumatol Engl Ed	2016	56(5)	406-413
		Sivas F, et al.	The Nottingham health profile in rheumatoid arthritis: correlation with other health status measurements and clinical variables	Rheumatol Int	2004	24(4)	203-206
		Uutela T, et al.	Validity of the Nottingham Health Profile in a Finnish out-patient population with rheumatoid arthritis	Rheumatology	2003	42(7)	841-845
		Wilburn J, et al.	Further international adaptation and validation of the Rheumatoid Arthritis Quality of Life (RAQoL) questionnaire	Rheumatol Int	2015	35(4)	669-675
		Zlatkovic-Svenda M, et al.	Adaptation and validation of the Rheumatoid Arthritis Quality of Life (RAQoL) questionnaire for use in Serbia	Rheumatol Int	2017	37(4)	641-646
9	**OES**	Dawson J, et al.	Specificity and responsiveness of patient-reported and clinician-rated outcome measures in the context of elbow surgery, comparing patients with and without rheumatoid arthritis	Orthop Traumatol Surg Res	2012	98(6)	652-658
10	**PDQ (PainDetect)**	Rifbjerg-Madsen S, et al.	Psychometric properties of the painDETECT questionnaire in rheumatoid arthritis, psoriatic arthritis and spondyloarthritis: Rasch analysis and test-retest reliability	Health Qual Life Outcomes	2017	15(1)	110
11	**PROMIS-29-Profile**	Katz P, et al.	Performance of the Patient-Reported Outcomes Measurement Information System 29-Item Profile in Rheumatoid Arthritis, Osteoarthritis, Fibromyalgia, and Systemic Lupus Erythematosus	Arthritis Care Res	2017	69(9)	312-1321
12	**PROMIS-PI 3a-SF**	Bartlett SJ, et al.	Reliability and Validity of Selected PROMIS Measures in People with Rheumatoid Arthritis	PLoS One	2015	10(9)	:0138543
		Wohlfahrt A, et al.	Responsiveness of Patient-Reported Outcomes Measurement Information System Measures in Rheumatoid Arthritis Patients Starting or Switching a Disease-Modifying Antirheumatic Drug	Arthritis Care Res (Hoboken)	2019	71(4)	521-529
13	**RAND-36**	Laas K, et al.	Early improvement of health-related quality of life during treatment with etanercept and adalimumab in patients with rheumatoid arthritis in routine practice.	Clin Exp Rheumatol	2009	27(2)	315-20
14	**RAOS**	Bremander AB, et al.	Validation of the Rheumatoid and Arthritis Outcome Score (RAOS) for the lower extremity	Health Qual Life Outcomes	2003	1	55
		Duval A, et al.	Cross-cultural adaptation and validation of the French version of the Rheumatoid and Arthritis Outcome Score (RAOS)	Clin Exp Rheumatol	2010	28(6)	806-812
		Karatepe AG, et al.	The validity and reliability of the Turkish version of the Rheumatoid and Arthritis Outcome Score (RAOS) in patients with rheumatoid arthritis	Clin Rheumatol	2009	28(1)	47-51
		Negahban H, et al.	Reliability, validity, and responsiveness of the Persian version of the rheumatoid and arthritis outcome score (RAOS) in patients with rheumatoid arthritis	Clin Rheumatol	2015	34(1)	35-42
15	**RAPS**	Anderson DL	Development of an Instrument to Measure Pain in Rheumatoid Arthritis: Rheumatoid Arthritis Pain Scale (RAPS)	Arthritis Care Res	2001	45(4)	317-323
		Kianifard T, et al.	Validation and relevance of Rheumatoid Arthritis Pain Scale (RAPS) in Indian (Asian) patients suffering from rheumatoid arthritis	Clin Rheumatol	2016	35(1)	63-71
		Singh H, et al.	The Validity and Sensitivity of Rheumatoid Arthritis Pain Scale on a Different Ethnic Group From Indian Rheumatoid Arthritis Patients	Arch Rheumatol	2020	35(1)	90-96

		Tofani M, et al.	The Italian version of rheumatoid arthritis pain scale (IT-RAPS): Psychometric properties on community and clinical samples	Reumatismo	2019	71(1)	13-18
**16**	**SACRAH**	Leeb BF, et al.	SACRAH: a score for assessment and quantification of chronic rheumatic affections of the hands	Rheumatology	2003	42(10)	173-1178
**17**	**SAFE-Q**	Yano K, et al.	Validity and responsiveness of a self-administered foot evaluation questionnaire in rheumatoid arthritis	Mod Rheumatol	2015	25(3)	358-361
**18**	**SF-36**	Cella D, et al.	Validation of the Functional Assessment of Chronic Illness Therapy Fatigue Scale Relative to Other Instrumentation in Patients with Rheumatoid Arthritis	J Rheumatol	2005	32(5)	811-819
		Chogle AR, et al.	Comparison of the Indian version of Health Assessment Questionnaire Score and Short Form 36 Physical Function Score in rheumatoid arthritis using Rasch analysis	Indian J Rheumatol	2008	3(2)	52-57
		Dures EK, et al.	Reliability and sensitivity to change of the Bristol Rheumatoid Arthritis Fatigue scales	Rheumatology	2013	52(10)	832-1839
		Durmus B, et al.	Can the patient-reported outcome instruments determine disease activity in rheumatoid arthritis?	Bratisl Lek Listy	2011	112(10)	555-561
		Englbrecht M, et al.	Measuring pain and efficacy of pain treatment in inflammatory arthritis: a systematic literature review	J Rheumatol	2012	90	3-10
		Feroz AH, et al.	The Bengali Short Form-36 was acceptable, reliable, and valid in patients with rheumatoid arthritis	J Clin Epidemiol	2012	65(11)	227-1235
		Gao L, et al.	Psychometric properties of the Chinese version of Arthritis Self-Efficacy Scale-8 (ASES-8) in a rheumatoid arthritis population	Rheumatol Int	2017	37(5)	751-756
		Hammond A, et al.	The reliability and validity of the English version of the Evaluation of Daily Activity Questionnaire for people with rheumatoid arthritis	Rheumatology	2015	54(9)	605-1615
		Hays RD, et al.	Responsiveness and minimally important difference for the Patient-Reported Outcomes Measurement Information System (PROMIS) 20-item physical functioning short form in a prospective observational study of rheumatoid arthritis	Ann Rheum Dis	2015	74(1)	104-107
		Hurst NP, et al.	Comparison of the MOS short form-12 (SF12) health status questionnaire with the SF36 in patients with rheumatoid arthritis	Br J Rheumatol	1998	37(8)	862-869
		Koh ET, et al.	The reliability, validity and sensitivity to change of the Chinese version of SF-36 in oriental patients with rheumatoid arthritis	Rheumatology	2006	45(8)	023-1028
		Kvien TK, et al.	Performance of the Norwegian SF-36 Health Survey in Patients with Rheumatoid Arthritis. II. A Comparison of the SF-36 with Disease-Specific Measures	J Clin Epidemiol	1998	51(11)	077-1086
		Lima Eda S, et al.	Translation, cultural adaptation and reproducibility of the Oxford Shoulder Score questionnaire for Brazil, among patients with rheumatoid arthritis	Sao Paulo Med J	2016	134(1)	40-46
		Linde L, et al.	Health-Related Quality of Life: Validity, Reliability, and Responsiveness of SF-36, EQ-15D, EQ-5D, RAQoL, and HAQ in Patients with Rheumatoid Arthritis	J Rheumatol	2008	35(8)	528-1537
		Martin M, et al.	Item response theory methods can improve the measurement of physical function by combining the modified health assessment questionnaire and the SF-36 physical function scale	Qual Life Res	2007	16(4)	647-660
		Nazary-Moghadam S, et al.	Adaptation, reliability and validity testing of a Persian version of the Health Assessment Questionnaire-Disability Index in Iranian patients with rheumatoid arthritis	J Bodyw Mov Ther	2017	21(1)	133-140
		Negahban H, et al.	Reliability, validity, and responsiveness of the Persian version of the rheumatoid and arthritis outcome score (RAOS) in patients with rheumatoid arthritis	Clin Rheumatol	2015	34(1)	35-42
		Nicklin J, et al.	Measuring Fatigue in Rheumatoid Arthritis: A Cross-Sectional Study to Evaluate the Bristol Rheumatoid Arthritis Fatigue Multi-Dimensional Questionnaire, Visual Analog Scales, and Numerical Rating Scales	Arthritis Care Res	2010	62(11)	559-1568
		Oude Voshaar MA, et al.	Assessment of Fatigue in Rheumatoid Arthritis: A Psychometric Comparison of Single-item, Multiitem, and Multidimensional Measures	J Rheumatol	2015	42(3)	413-20
		Oude Voshaar MA, et al.	Measuring Disease Exacerbation and Flares in Rheumatoid Arthritis: Comparison of Commonly Used Disease Activity Indices and Individual Measures.	J Rheumatol	2017	44(8)	118-1124
		Oude Voshaar MA, et al.	Validity and measurement precision of the PROMIS physical function item bank and a content validity driven 20-item short form in rheumatoid arthritis compared with traditional measures	Rheumatology	2015	54(12)	221-2229
		Pakpour AH, et al.	Health-related quality of life in young adult patients with rheumatoid arthritis in Iran: reliability and validity of the Persian translation of the PedsQL™ 4.0 Generic Core Scales Young Adult Version	Clin Rheumatol	2013	32(1)	15-22
		Pouchot J, et al.	Determination of the minimal clinically important difference for seven fatigue measures in rheumatoid arthritis	J Clin Epidemiol	2008	61(7)	705-713
		Russell AS, et al.	The responsiveness of generic health status measures as assessed in patients with rheumatoid arthritis receiving infliximab	J Rheumatol	2003	30(5)	941-947
		Ruta D, et al.	Measuring health status in British patients with rheumatoid arthritis: reliability, validity and responsiveness of the short form 36-item health survey (SF-36)	Br J Rheumatol	1998	37(4)	425-436
		Strand V, et al.	Health-related Quality of Life Outcomes of Adalimumab for Patients with Early Rheumatoid Arthritis: Results from a Randomized Multicenter Study	J Rheumatol	2012	39(1)	63-72
		Taylor WJ & Mcpherson KM	Using Rasch Analysis to Compare the Psychometric Properties of the Short Form 36 Physical Function Score and the Health Assessment Questionnaire Disability Index in Patients With Psoriatic Arthritis and Rheumatoid Arthritis	Arthritis Rheum	2007	57(5)	723-729
		ten Klooster PM, et al.	Development and evaluation of a crosswalk between the SF-36 physical functioning scale and Health Assessment Questionnaire disability index in rheumatoid arthritis	Health Qual Life Outcomes	2013	11	199
		ten Klooster PM, et al.	Performance of the Dutch SF-36 version 2 as a measure of health-related quality of life in patients with rheumatoid arthritis	Health Qual Life Outcomes	2013	11	77
		Tugwell P, et al.	Clinical improvement as reflected in measures of function and health-related quality of life following treatment with leflunomide compared with methotrexate in patients with rheumatoid arthritis: sensitivity and relative efficiency to detect a treatment	Arthritis Rheum	2000	43(3)	506-514
		Veehof MM, et al.	Comparison of Internal and External Responsiveness of the Generic Medical Outcome Study Short Form-36 (SF-36) with Disease-specific Measures in Rheumatoid Arthritis	J Rheumatol	2008	35(4)	610-617
		Ward MM, et al.	Clinically important changes in short form-36 scales for use in rheumatoid arthritis clinical trials: the impact of low responsiveness	Arthritis Care Res	2014	66(12)	783-1789
		Weinblatt ME, et al.	Effects of Fostamatinib (R788), an Oral Spleen Tyrosine Kinase Inhibitor, on Health-related Quality of Life in Patients with Active Rheumatoid Arthritis: Analyses of Patient-reported Outcomes from a Randomized, Double-blind, Placebo-controlled Trial	J Rheumatol	2013	40(4)	369-378
		Wells G, et al.	Responsiveness of patient reported outcomes including fatigue, sleep quality, activity limitation, and quality of life following treatment with abatacept for rheumatoid arthritis	Ann Rheum Dis	2008	67(2)	260-265
		Wolfe F	Fatigue Assessments in Rheumatoid Arthritis: Comparative Performance of Visual Analog Scales and Longer Fatigue Questionnaires in 7760 Patients	J Rheumatol	2004	31(10)	896-1902
**19**	**SF-MPQ**	Burckhard CS, et al.	A Swedish Version of the Short-Form McGill Pain Questionnaire	Scand J Rheumatol	1994	23(2)	77-81
		Hawker GA, et al.	Measures of adult pain: Visual Analog Scale for Pain (VAS Pain), Numeric Rating Scale for Pain (NRS Pain), McGill Pain Questionnaire (MPQ), Short-Form McGill Pain Questionnaire (SF-MPQ), Chronic Pain Grade Scale (CPGS), Short Form-36 Bodily Pain Sca	Arthritis Care Res	2011	63(Suppl 11)	240-S252
		Yakut Y, et al.	Reliability and validity of the Turkish version short-form McGill pain questionnaire in patients with rheumatoid arthritis	Clin Rheumatol	2007	26(7)	083-1087
**20**	**WOMAC**	Wolfe F	Determinants of WOMAC function, pain and stiffness scores: evidence for the role of low back pain, symptom counts, fatigue and depression in osteoarthritis, rheumatoid arthritis and fibromyalgia.	Rheumatology	1999	38(4)	355-361
		Wolfe F & Kong SX	Rasch analysis of the Western Ontario MacMaster questionnaire (WOMAC) in 2205 patients with osteoarthritis, rheumatoid arthritis, and fibromyalgia.	Ann Rheum Dis	1999	58(9)	563-568
**21**	**BRAF-MDQ**	Dures EK, et al.	Reliability and sensitivity to change of the Bristol Rheumatoid Arthritis Fatigue scales	Rheumatology	2013	52(10)	832-1839
		Feldthusen C, et al.	EXPLANATORY FACTORS AND PREDICTORS OF FATIGUE IN PERSONS WITH RHEUMATOID ARTHRITIS: A LONGITUDINAL STUDY	J Rehabil Med	2016	48(5)	469-476
		Gao L, et al.	Validity and Reliability of the Chinese Version of FACIT-F and BRAF-MDQ in Rheumatoid Arthritis Patients	Chinese General Practice	2019	22(17)	111-2115
		Hewlett S, et al.	The revised Bristol Rheumatoid Arthritis Fatigue measures and the Rheumatoid Arthritis Impact of Disease scale: validation in six countries	Rheumatology	2018	57(2)	300-308
		Kirwan J, et al.	Validity and Responsiveness of the Bristol Rheumatoid Arthritis Fatique Multidimensional Questionnaire (BRAF-MDQ) in a Randomized Clinical Trial	Value Health	2014	17(7)	A568-569
		Nicklin J, et al.	Measuring Fatigue in Rheumatoid Arthritis: A Cross-Sectional Study to Evaluate the Bristol Rheumatoid Arthritis Fatigue Multi-Dimensional Questionnaire, Visual Analog Scales, and Numerical Rating Scales	Arthritis Care Res	2010	62(11)	559-1568
		Nikolaus S, et al.	Construct Validation of a Multidimensional Computerized Adaptive Test for Fatigue in Rheumatoid Arthritis	PLoS One	2015	10(12)	:0145008
		Oude Voshaar MA, et al.	Assessment of Fatigue in Rheumatoid Arthritis: A Psychometric Comparison of Single-item, Multiitem, and Multidimensional Measures	J Rheumatol	2015	42(3)	413-20
		Oude Voshaar MAH, et al.	Psychometric properties and cross-language equivalence of the revised Bristol Rheumatoid Arthritis Fatigue and the Rheumatoid Arthritis Impact of Disease scales in rheumatoid arthritis	Quality of Life Research	2019	28(9)	543-2552
		Sarı F, et al.	Reliability, validity, and cross-cultural adaptation of the Turkish version of the Bristol Rheumatoid Arthritis Fatigue Multi-Dimensional Questionnaire	Clinical Rheumatology	2018	37(6)	465-1470
**22**	**CFS**	Lwin CTT, et al.	The assessment of fatigue in primary Sjogren's syndrome	Scand J Rheumatol	2003	32(1)	33-37
		Pouchot J, et al.	Determination of the minimal clinically important difference for seven fatigue measures in rheumatoid arthritis	J Clin Epidemiol	2008	61(7)	705-713
**23**	**CIS8R**	Evers AW, et al.	Tailored cognitivebehavioral therapy in early rheumatoid arthritis for patients at risk: an RCT	Pain	2002	100(1-2)	141-153
		Hoving JL, et al.	Perceived work ability, quality of life and fatigue in patients with rheumatoid arthritis after a 6 month course of TNF inhibitors: prospective intervention study and partial economic evaluation.	Scand J Rheumatol	2009	38(4)	246-250
		Van Hoogmoed D, et al.	How to assess fatigue in rheumatoid arthritis: validity and reliability of the Checklist Individual Strength [abstract]	Arthritis Rheum	2008	58(suppl)	S868
**24**	**FACIT-F**	Cella D, et al.	Validation of the Functional Assessment of Chronic Illness Therapy Fatigue Scale Relative to Other Instrumentation in Patients with Rheumatoid Arthritis	J Rheumatol	2005	32(5)	811-819
		Dures EK, et al.	Reliability and sensitivity to change of the Bristol Rheumatoid Arthritis Fatigue scales	Rheumatology	2013	52(10)	832-1839
		Gao L, et al.	Psychometric properties of the Chinese version of Arthritis Self-Efficacy Scale-8 (ASES-8) in a rheumatoid arthritis population	Rheumatol Int	2017	37(5)	751-756
		Gao L, et al.	Validity and Reliability of the Chinese Version of FACIT-F and BRAF-MDQ in Rheumatoid Arthritis Patients	Chinese General Practice	2019	22(17)	111-2115
		Pouchot J, et al.	Determination of the minimal clinically important difference for seven fatigue measures in rheumatoid arthritis	J Clin Epidemiol	2008	61(7)	705-713
**25**	**FSS**	Cross M, et al.	Association Between Measures of Fatigue and Health-Related Quality of Life in Rheumatoid Arthritis and Osteoarthritis	Patient	2008	1(2)	97-104
		Mancuso CA, et al.	Psychosocial variables and fatigue: a longitudinal study comparing individuals with rheumatoidarthritis and healthy controls.	J Rheumatol	2006	33(8)	496-1502
		Pouchot J, et al.	Determination of the minimal clinically important difference for seven fatigue measures in rheumatoid arthritis	J Clin Epidemiol	2008	61(7)	705-713
**26**	**MAF**	Belza BL	Comparison of self-reported fatigue in rheumatoid arthritis and controls	J Rheumatol	1995	22(4)	639-643
		Belza BL, et al.	Correlates of fatigue in older adults with rheumatoid arthritis	Nurs Res	1993	42(2)	93-99
		Cella D, et al.	Validation of the Functional Assessment of Chronic Illness Therapy Fatigue Scale Relative to Other Instrumentation in Patients with Rheumatoid Arthritis	J Rheumatol	2005	32(5)	811-819
		Jump RL, et al.	History of affective disorder and the experience of fatigue in rheumatoid arthritis	Arthritis Rheum	2004	51(2)	239-245
		Pouchot J, et al.	Determination of the minimal clinically important difference for seven fatigue measures in rheumatoid arthritis	J Clin Epidemiol	2008	61(7)	705-713
		Wolfe F	Fatigue Assessments in Rheumatoid Arthritis: Comparative Performance of Visual Analog Scales and Longer Fatigue Questionnaires in 7760 Patients	J Rheumatol	2004	31(10)	896-1902
**27**	**MFI**	Pouchot J, et al.	Determination of the minimal clinically important difference for seven fatigue measures in rheumatoid arthritis	J Clin Epidemiol	2008	61(7)	705-713
**28**	**POMS-Fatigue**	Balsamo S, et al.	Exercise and Fatigue in Rheumatoid Arthritis	Isr Med Assoc J	2014	16(1)	57-60
		Belza BL	Comparison of self-reported fatigue in rheumatoid arthritis and controls	J Rheumatol	1995	22(4)	639-643
		Dures EK, et al.	Reliability and sensitivity to change of the Bristol Rheumatoid Arthritis Fatigue scales	Rheumatology	2013	52(10)	832-1839
		Jamison M, et al.	Correlates of Falls and Fear of Falling Among Adults With Rheumatoid Arthritis	Arthritis Rheum	2003	49(5)	673-680
		Tack BB	Self-Reported Fatigue in Rheumatoid Arthritis. A pilot study	Arthritis Care Res	1990	3(3)	154-157
**29**	**PROMIS Fatigue 4a**	Bingham III CO, et al.	PROMIS Fatigue short forms are reliable and valid in adults with rheumatoid arthritis	J Patient Rep Outcomes	2019	3(1)	14

30	**PROMIS Fatigue 7a**	Bartlett SJ, et al.	Combining online and in-person methods to evaluate the content validity of PROMIS fatigue short forms in rheumatoid arthritis	Qual Life Res	2018	27(9)	443-2451
		Bingham III CO, et al.	PROMIS Fatigue short forms are reliable and valid in adults with rheumatoid arthritis	J Patient Rep Outcomes	2019	3(1)	14
31	**PROMIS Fatigue 8a**	Bartlett SJ, et al.	Combining online and in-person methods to evaluate the content validity of PROMIS fatigue short forms in rheumatoid arthritis	Qual Life Res	2018	27(9)	443-2451
		Bingham III CO, et al.	PROMIS Fatigue short forms are reliable and valid in adults with rheumatoid arthritis	J Patient Rep Outcomes	2019	3(1)	14
32	**SVS**	Rouse PC, et al.	Measuring the positive psychological wellbeing of people with rheumatoid arthritis: a cross-sectional validation of the subjective vitality scale	Arthritis Res Ther	2015	17	312
33	**AIMS2-SF**	Askary-Ashtiani AR, et al.	Translation and validation of the Persian version of the Arthritis Impact Measurement Scales 2-Short Form (AIMS2-SF) in patients with rheumatoid arthritis	Clin Rheumatol	2009	28(5)	521-527
		Guillemin F, et al.	THE AIMS2-SF: A Short Form of the Arthritis Impact Measurement Scales 2	Arthritis Rheum	1997	40(7)	267-1274
		Haavardsholm EA, et al.	A comparison of agreement and sensitivity to change between AIMS2 and a short form of AIMS2 (AIMS2-SF) in more than 1,000 rheumatoid arthritis patients	J Rheumatol	2000	27(12)	810-2816
		Josefsson KA, et al.	Swedish version of the multi dimensional health assessment questionnaire – translation and psychometric evaluation	BMC Musculoskelet Disord	2013	14	178
		Negahban H, et al.	Reliability, validity, and responsiveness of the Persian version of the rheumatoid and arthritis outcome score (RAOS) in patients with rheumatoid arthritis	Clin Rheumatol	2015	34(1)	35-42
		Taal E, et al.	Psychometric properties of a Dutch short form of the Arthritis Impact Measurement Scales 2 (Dutch-AIMS2-SF)	Rheumatology	2003	42(3)	427-434
		Taal E, et al.	Sensitivity to change of AIMS2 and AIMS2-SF components in comparison to M-HAQ and VAS-pain	Ann Rheum Dis	2004	63(12)	655-1658
		ten Klooster PM, et al.	Confirmatory Factor Analysis of the Arthritis Impact Measurement Scales 2 Short Form in Patients With Rheumatoid Arthritis	Arthritis Rheum	2008	59(5)	692-698
34	**CES-D**	Blalock SJ, et al.	Validity of the Center for Epidemiological Studies Depression Scale in Arthritis Population	Arthritis Rheum	1989	32(8)	991-997
		Callahan LF, et al.	The Beck Depression Inventory, Center for Epidemiological Studies Depression Scale (CES-D), and General Well-Being Schedule Depression Subscale in Rheumatoid Arthritis	Arthritis Care Res	1991	4(1)	3-11
		Cho MJ, et al.	Concordance between two measures of depression in the Hispanic Health and Nutrition Examination Survey	Soc Psychiatry Psychiatr Epide	1993	28(4)	156-163
		Covic T, et al.	A longitudinal evaluation of the Center for Epidemiologic Studies-Depression scale (CES-D) in a Rheumatoid Arthritis Population using Rasch Analysis	Health Qual Life Outcomes	2007	5(41)	1-8
		Covic T, et al.	Variability in depression prevalence in early rheumatoid arthritis: a comparison of the CES-D and HAD-D Scales	BMC Musculoskelet Disord	2009	10(18)	1-9
		McQuillan J, et al.	A comparison of self-reports of distress and affective disorder diagnoses in rheumatoid arthritis: A receiver operator characteristic analysis	Arthritis Care Res	2003	49(3)	368-376
		Rhee SH, et al.	A Confirmatory Factor Analysis of the Center for Epidemiologic Studies Depression Scale in Rheumatoid Arthritis Patients: Additional Evidence for a Four-Factor Model	Arthritis Care Res	1999	12(6)	392-400
35	**CSHQ-RA**	Chiou CF, et al.	Development and Validation of the Revised Cedars-Sinai Health-Related Quality of Life for Rheumatoid Arthritis Instrument	Arthritis Rheum	2006	55(6)	856-863
		Chiou CF, et al.	Revalidation of the Original Cedars-Sinai Health-Related Quality of Life in Rheumatoid Arthritis Questionnaire	J Rheumatol	2006	33(2)	256-262
		Russak SM, et al.	Validation of a Rheumatoid Arthritis Health-Related Quality of Life Instrument, the CSHQ-RA	Arthritis Rheum	2003	49(6)	798-803
		Weisman MH, et al.	Development of a New Instrument for Rheumatoid Arthritis: The Cedars-Sinai Health-Related Quality of Life Instrument (CSHQ-RA)	Arthritis Rheum	2003	49(1)	78-84
36	**DASS**	Covic T, et al.	Depression and Anxiety in Patients with Rheumatoid Arthritis: Prevalence rates based on a comparison of the Depression, Anxiety and Stress Scale (DASS) and the Hospital, Anxiety and Depression Scale (HADS)	BMC Psychiatry	2012	12	6
37	**EIS**	Sinclair VG & Dowdy SW	Development and validation of Emotional Intimacy Scale	J Nurs Meas	2005	13(3)	193-206
38	**HADS**	Covic T, et al.	Depression and Anxiety in Patients with Rheumatoid Arthritis: Prevalence rates based on a comparison of the Depression, Anxiety and Stress Scale (DASS) and the Hospital, Anxiety and Depression Scale (HADS)	BMC Psychiatry	2012	12	6
		Covic T, et al.	Variability in depression prevalence in early rheumatoid arthritis: a comparison of the CES-D and HAD-D Scales	BMC Musculoskelet Disord	2009	10	18
		Gao L, et al.	Psychometric properties of the Chinese version of Arthritis Self-Efficacy Scale-8 (ASES-8) in a rheumatoid arthritis population	Rheumatol Int	2017	37(5)	751-756
		Hill J, et al.	The arthritis impact measurement scales: an anglicized version to assess the outcome of British patients with rheumatoid arthritis	Br J Rheumatol	1990	29(3)	193-196
		Kojima M, et al.	Psychosocial factors, disease status, and quality of life in patients with rheumatoid arthritis	J Psychosom Res	2009	67(5)	425-431
		Lundgren S, et al.	The Swedish version of the Multidimensional Health Locus of Control scales, form C. Aspects of reliability and validity in patients with rheumatoid arthritis	Adv Physiother	2007	9(1)	16-22
		Rouse PC, et al.	Measuring the positive psychological wellbeing of people with rheumatoid arthritis: a cross-sectional validation of the subjective vitality scale	Arthritis Res Ther	2015	17	312
		Suarez-Mendoza AA, et al.	Measurement of Depression in Mexican Patients with Rheumatoid Arthritis: Validity of the Beck Depression Inventory	Arthritis Care Res	1997	10(3)	194-199
		Traki L, et al.	Responsiveness of the EuroQol EQ-5D and Hospital Anxiety and Depression Scale (HADS) in rheumatoid arthritis patients receiving tocilizumab	Clin Rheumatol	2014	33(8)	055-1060
39	**PVS**	Sinclair VG & Wallston KA	Psychological Vulnerability Predicts Increases in Depressive Symptoms in Individuals With Rheumatoid Arthritis	Nurs Res	2010	59(2)	140-146
		Sinclair VG & Wallston KA	The Development and Validation of the Psychological Vulnerability Scale	Cognit Ther Res	1999	23(2)	119-129
40	**WHOQoL-Bref**	Taylor WJ, et al.	Quality of life of people with rheumatoid arthritis as measured by the World Health Organization Quality of Life Instrument, short form (WHOQOL-BREF): score distributions and psychometric properties.	Arthritis Rheum	2004	51(3)	350-357
41	**AIS**	Westhovens R, et al.	Sleep Problems in Patients with Rheumatoid Arthritis	J Rheumatol	2014	41(1)	31-40
42	**JSS**	Jenkins DC, et al.	A scale for the estimation of sleep problems in clinical research	J Clin Epidemiol	1988	41(4)	313-321
43	**MOS-Sleep**	Wells G, et al.	Investigation into the impact of abatacept on sleep quality in patients with rheumatoid arthritis, and the validity of the MOS-Sleep questionnaire Sleep Disturbance Scale	Ann Rheum Dis	2010	69(10)	768-1773
44	**ABES**	Boyington JE, et al.	Factor Structure of the Arthritis Body Experience Scale (ABES) in a U.S. Population of People with Osteoarthritis (OA), Rheumatoid Arthritis (RA), Fibromyalgia (FM) and Other Rheumatic Conditions	Open Rheumatol J	2008	2	64-70
45	**FIS**	Helliwell P, et al.	Development of a Foot Impact Scale for Rheumatoid Arthritis	Arthritis Rheum	2005	53(3)	418-422
		Woodburn J, et al.	Adaptation and Crosscultural Validation of the Foot Impact Scale for Rheumatoid Arthritis Using Rasch Analysis	Arthritis Care Res	2012	64(7)	986-992
		Woodburn J, et al.	Rasch analysis of Dutch-translated version of the Foot Impact Scale for rheumatoid arthritis	Rheumatology	2011	50(7)	315-1319
46	**PDQ-SF**	Julian LJ, et al.	Validity of Brief Screening Tools for Cognitive Impairment in Rheumatoid Arthritis and Systemic Lupus Erythematosus	Arthritis Care Res	2012	64(3)	448-454
47	**RADAI-5**	Bossert M, et al.	Evaluation of self-report questionnaires for assessing rheumatoid arthritis activity: a cross-sectional study of RAPID3 and RADAI5 and flare detection in 200 patients	Joint Bone Spine	2012	79(1)	57-62
		Leeb BF, et al.	Patient-centered rheumatoid arthritis disease activity assessment by a modified RADAI	J Rheumatol	2008	35(7)	294-1299
		Leeb BF, et al.	A comparison of patient questionnaires and composite indexes in routine care of rheumatoid arthritis patients	Joint Bone Spine	2009	76(6)	658-64
		Rintelen B, et al.	Remission in rheumatoid arthritis: a comparison of the 2 newly proposed ACR/EULAR remission criteria with the rheumatoid arthritis disease activity index-5, a patient self-report disease activity index	J Rheumatol	2013	40(4)	394-400
		Rintelen B, et al.	The rheumatoid arthritis disease activity index-5 in daily use. Proposal for disease activity categories	J Rheumatol	2009	36(5)	918-924
		Salaffi F, et al.	New Indices study group. The comparative responsiveness of the patient self-report questionnaires and composite disease indices for assessing rheumatoid arthritis activity in routine care	Clin Exp Rheumatol	2012	30(6)	912-921
		Sunar I, et al.	Translation and validation of the Turkish language version of the Rheumatoid Arthritis Disease Activity Index-5	Int J Rheum Dis	2017	20(12)	012-2019
		Uhlig T, et al.	Test-retest reliability of disease activity core set measures and indices in rheumatoid arthritis	Ann Rheum Dis	2009	68(6)	972-975
48	**RASQ**	Banderas B, et al.	Development of the Rheumatoid Arthritis Symptom Questionnaire (RASQ): a patient reported outcome scale for measuring symptoms of rheumatoid arthritis	Curr Med Res Opin	2017	33(9)	643-1651
		Banderas B, et al.	Psychometric evaluation of the Rheumatoid Arthritis Symptom Questionnaire (RASQ) in an observational study	Curr Med Res Opin	2017	33(12)	121-2128
49	**SF-12**	Dritsaki M, et al.	An empirical evaluation of the SF-12, SF-6D, EQ-5D and Michigan Hand Outcome Questionnaire in patients with rheumatoid arthritis of the hand	Health Qual Life Outcomes	2017	15(1)	20
		Hu H, et al.	Psychometric validation of Chinese Health Assessment Questionnaire for use in rheumatoid arthritis patients in China	Int J Rheum Dis	2017	20(12)	987-1992
		Hurst NP, et al.	Comparison of the MOS short form-12 (SF12) health status questionnaire with the SF36 in patients with rheumatoid arthritis	Br J Rheumatol	1998	37(8)	862-869
50	**EDAQ**	Hammond A, et al.	Linguistic validation and cultural adaptation of an English version of the Evaluation of Daily Activity Questionnaire in rheumatoid arthritis	Health Qual Life Outcomes	2014	12	143
		Hammond A, et al.	The reliability and validity of the English version of the Evaluation of Daily Activity Questionnaire for people with rheumatoid arthritis	Rheumatology	2015	54(9)	605-1615
51	**IPAQ-SF**	Tierneya M, et al.	Criterion validity of the International Physical ActivityQuestionnaire Short Form (IPAQ-SF) for use in patients withrheumatoid arthritis: comparison with the SenseWear Armband	Physiotherapy	2015	101(2)	193-197
52	**ROAD**	Salaffi F, et al.	Classical test theory and Rasch analysis validation of the Recent-Onset Arthritis Disability questionnaire in rheumatoid arthritis patients	Clin Rheumatol	2013	32(2)	211-217
		Salaffi F, et al.	Comparison of the Recent-Onset Arthritis Disability questionnaire with the Health Assessment Questionnaire disability index in patients with rheumatoid arthritis.	Clin Exp Rheumatol	2010	28(6)	855-865
		Salaffi F, et al.	Development of a functional disability measurement tool to assess early arthritis: the Recent-Onset Arthritis Disability (ROAD) questionnaire.	Clin Exp Rheumatol	2005	23(5)	628-636
		Salaffi F, et al.	Measuring functional disability in early rheumatoid arthritis: the validity, reliability and responsiveness of the Recent-Onset Arthritis Disability (ROAD) index	Clin Exp Rheumatol	2005	3(5 Suppl 39)	S31-42
		Salaffi F, et al.	The use of computer touch-screen technology for the collection of patient-reported outcome data in rheumatoid arthritis: comparison with standardized paper questionnaires	Clin Exp Rheumatol	2009	27(3)	459-468
53	**WHODAS-II**	Meesters JJ, et al.	Validity and responsiveness of the World Health Organization Disability Assessment Schedule II to assess disability in rheumatoid arthritis patients	Rheumatology	2010	49(2)	326-333
54	**CQR**	Lee JY, et al.	Cultural Adaptation of a Compliance Questionnaire for Patients with Rheumatoid Arthritis to a Korean Version	Korean J Intern Med	2011	26(1)	28-33
		Salgado E, et al.	Spanish transcultural adaptation and validation of the English version of the compliance questionnaire in rheumatology	Rheumatology International	2018	38(3)	467-472
55	**CQR-5**	Hughes LD, et al.	A 5 item version of the Compliance Questionnaire for Rheumatology (CQR5) successfully identifies low adherence to DMARDs	BMC Musculoskelet Disord	2013	14	286
		Ometto F, et al.	Adherence in rheumatoid arthritis patients assessed with a validated Italian version of the 5-item Compliance Questionnaire for Rheumatology	Clin Exp Rheumatol	2019	37(6)	915-922
56	**MARS-5**	De Cuyper E, et al.	Determinants of methotrexate adherence in rheumatoid arthritis patients	Clin Rheumatol	2016	35(5)	335-1339
57	**MARS-9**	Salt E, et al.	Psychometric properties of three medication adherence scales in patients with rheumatoid arthritis	J Nurs Meas	2012	20(1)	59-72
58	**MAS**	Salt E, et al.	Psychometric properties of three medication adherence scales in patients with rheumatoid arthritis	J Nurs Meas	2012	20(1)	59-72
59	**SFA**	van den Ende CH, et al.	Assessment of shoulder function in rheumatoid arthritis	J Rheumatol	1996	23(12)	043-2048
		Vermeulen HM, et al.	Responsiveness of the shoulder function assessment scale in patients with rheumatoid arthritis	Ann Rheum Dis	2006	65(2)	239-241
60	**EWPS**	Tang K, et al.	Sensibility of five at-work productivity measures was endorsed by patients with osteoarthritis or rheumatoid arthritis	J Clin Epidemiol	2013	66(5)	546-556
61	**RA-WIS**	Gilworth G, et al.	Adaptation and cross-cultural validation of the rheumatoid arthritis work instability scale (RA–WIS)	Ann Rheum Dis	2009	68(11)	686-1690

		Gilworth G, et al.	Development of a Work Instability Scale for Rheumatoid Arthritis	Arthritis Rheum	2003	49(3)	349-354
		Revicki D, et al.	Reliability and Validity of the Work Instability Scale for Rheumatoid Arthritis	Value Health	2015	18(8)	008-1015
		Tang K, et al.	Disease-Related Differential Item Functioning in the Work Instability Scale for Rheumatoid Arthritis: Converging Results From Three Methods	Arthritis Care Res	2011	63(8)	159-1169
		Tang K, et al.	Sensibility of five at-work productivity measures was endorsed by patients with osteoarthritis or rheumatoid arthritis	J Clin Epidemiol	2013	66(5)	546-556
**62**	**SPS-6**	Tang K, et al.	Sensibility of five at-work productivity measures was endorsed by patients with osteoarthritis or rheumatoid arthritis	J Clin Epidemiol	2013	66(5)	546-556
**63**	**VOLP**	Zhang W, et al.	Development of a Composite Questionnaire, the Valuation of Lost Productivity, to Value Productivity Losses: Application in Rheumatoid Arthritis	Value Health	2012	15(1)	46-54
		Zhang W, et al.	Measuring Time Input Loss Among Patients With Rheumatoid Arthritis Validity and Reliability of the Valuation of Lost Productivity Questionnaire	J Occup Environ Med	2011	53(5)	530-536
**64**	**WALS**	Tang K, et al.	Sensibility of five at-work productivity measures was endorsed by patients with osteoarthritis or rheumatoid arthritis	J Clin Epidemiol	2013	66(5)	546-556
**65**	**Wfun**	Ishimaru T, et al.	Validity and responsiveness of the Work Functioning Impairment Scale (WFun) in rheumatoid arthritis patients: A multicenter prospective study	Mod Rheumatol	2020	30(5)	821-827
**66**	**WLQ**	Tang K, et al.	Sensibility of five at-work productivity measures was endorsed by patients with osteoarthritis or rheumatoid arthritis	J Clin Epidemiol	2013	66(5)	546-556
		Walker N, et al.	Work limitations among working persons with rheumatoid arthritis: results, reliability, and validity of the work limitations questionnaire in 836 patients	J Rheumatol	2005	32(6)	006-1012
**67**	**ABILHAND**	Arnould C, et al.	Can manual ability be measured with a generic ABILHAND scale? A cross-sectional study conducted on six diagnostic groups	BMJ Open	2012	2	e001807
		Başakçı Çalık B, et al.	Reliability and validity of the Turkish version of the ABILHAND questionnaire in rheumatoid arthritis individuals, based on rasch analysis	Arch Rheumatol	2019	34(4)	395-405
		Batcho CS, et al.	Responsiveness of the ABILHAND Questionnaire in Measuring Changes in Rheumatoid Arthritis Patients	Arthritis Care Res	2011	63(1)	135-141
		Durez P, et al.	Validation of the ABILHAND questionnaire as a measure of manual ability in patients with rheumatoid arthritis	Ann Rheum Dis	2007	66(8)	098-1105
		Penta M, et al.	ABILHAND: A Rasch-Built Measure of Manual Ability	Arch Phys Med Rehabil	1998	79(9)	038-1042
		Simone AM, et al.	Generic ABILHAND questionnaire can measure manual ability across a variety of motor impairments	Int J Rehabil Res	2011	34(2)	131-140
**68**	**CHFS**	Chiari A, et al.	Translation, cultural adaptation and reproducibility of the Cochin Hand Functional Scale questionnaire for Brazil	Clinics	2011	66(5)	731-736
		Duruöz MT, et al.	Development and validation of a rheumatoid hand functional disability scale that assesses functional handicap.	J Rheumatol	1996	23(7)	1167-72
		Lefevre-Colau MM, et al.	Responsiveness of the Cochin rheumatoid hand disability scale after surgery	Rheumatology	2001	40(8)	843-850
		Meireles SM, et al.	Cross-cultural adaptation and validation of the Michigan Hand Outcomes Questionnaire (MHQ) for Brazil: validation study	Sao Paulo Med J	2014	132(6)	339-347
		Poiraudeau S, et al.	The Ability of the Cochin Rheumatoid Arthritis Hand Functional Scale to Detect Change During the Course of Disease	Arthritis Care Res	2000	13(5)	296-303
		Poole JL, et al.	Reliability and Validity of a Self-report of Hand Function in Persons with Rheumatoid Arthritis	J Hand Ther	2006	19(1)	12-6
**69**	**DASH**	Aktekin LA, et al.	Disability of Arm Shoulder and Hand Questionnaire in rheumatoid arthritis patients: relationship with disease activity, HAQ, SF-36	Rheumatol Int	2011	31(6)	823-826
		Bilberg A, et al.	Disability of the Arm, Shoulder and Hand questionnaire in Swedish patients with Rheumatoid Arthritis : A validity study	J Rehabil Med	2012	44(1)	7-11
		Chiari-Grisar C, et al.	Performance of the Disabilities of the Arm, Shoulder and Hand Outcome Questionnaire and the Moberg Picking Up Test in Patients With Finger Joint Arthroplasty	Arch Phys Med Rehabil	2006	87(2)	203-206
		Dawson J, et al.	Specificity and responsiveness of patient-reported and clinician-rated outcome measures in the context of elbow surgery, comparing patients with and without rheumatoid arthritis	Orthop Traumatol Surg Res	2012	98(6)	652-658
		Hammond A, et al.	Linguistic validation, validity and reliability of the British English versions of the Disabilities of the Arm, Shoulder and Hand (DASH) questionnaire and QuickDASH in people with rheumatoid arthritis	BMC Musculoskelet Disord	2018	19	118
		Lima Eda S, et al.	Translation, cultural adaptation and reproducibility of the Oxford Shoulder Score questionnaire for Brazil, among patients with rheumatoid arthritis	Sao Paulo Med J	2016	134(1)	40-46
		Ochi K, et al.	Construct validity, reliability, response rate, and association with disease activity of the quick disabilities of the arm, shoulder and hand questionnaire in the assessment of rheumatoid arthritis	Mod Rheumatol	2015	25(2)	241-245
		Orfale AG, et al.	Translation into Brazilian Portuguese, cultural adaptation and evaluation of the reliability of the Disabilities of the Arm, Shoulder and Hand Questionnaire	Braz J Med Biol Res	2005	38(2)	293-302
		Prodinger B, et al.	Revisiting the disabilities of the arm, shoulder and hand (DASH) and QuickDASH in rheumatoid arthritis	BMC Musculoskelet Disord	2019	20	41
		Raven EE, et al.	Construct Validity and Reliability of the Disability of Arm, Shoulder and Hand Questionnaire for Upper Extremity Complaints in Rheumatoid Arthritis	J Rheumatol	2008	35(12)	12
		Yasui T, et al.	Relationship between roentgenographic joint destruction in the hands and functional disorders among patients with rheumatoid arthritis	Mod Rheumatol	2017	27(5)	828-832
**70**	**FFI-RS**	Rutkowski R, et al.	Adaptation and Validation of the Foot Function Index-Revised Short Form into Polish	Biomed Res Int	2017	2017	6051698
**71**	**HAQ**	Abourazzak FE, et al.	Psychometric evaluation of a Moroccan version of health assessment questionnaire for use in Moroccan patients with rheumatoid arthritis	Rheumatol Int	2008	28(12)	197-1203
		Adams J, et al.	Responsiveness of Self-Report and Therapist-Rated Upper Extremity Structural Impairment and Functional Outcome Measures in Early Rheumatoid Arthritis	Arthritis Care Res	2010	62(2)	274-278
		Aletaha D & Ward MM	Duration of rheumatoid arthritis influences the degree of functional improvement in clinical trials	Ann Rheum Dis	2006	65(2)	227-233
		Aletaha D, et al.	Treatment-related improvement in physical function varies with duration of rheumatoid arthritis: a pooled analysis of clinical trial results	Ann Rheum Dis	2008	67(2)	238-243
		Arvidson NG, et al.	Short-term effects of the TNFa antagonist infliximab on the acute phase reaction and activities of daily life in patients with rheumatoid arthritis	Scand J Clin Lab Invest	2007	67(3)	337-342
		Bardwell WA, et al.	Rheumatoid Arthritis Severity Scale: a brief, physician-completed scale not confounded by patient self-report of psychological functioning	Rheumatology	2002	41(1)	38-45
		Barra L, et al.	Real-World Anti-Tumor Necrosis Factor Treatment in Rheumatoid Arthritis, Psoriatic Arthritis, and Ankylosing Spondylitis: Cost-Effectiveness Based on Number Needed to Treat to Improve Health Assessment Questionnaire	J Rheumatol	2009	36(7)	421-1428
		Batcho CS, et al.	Responsiveness of the ABILHAND Questionnaire in Measuring Changes in Rheumatoid Arthritis Patients	Arthritis Care Res	2011	63(1)	135-141
		Benbouazza K, et al.	Profile and course of early rheumatoid arthritis in Morocco: a two-year follow-up study	BMC Musculoskelet Disord	2011	12	266
		Chogle AR, et al.	Comparison of the Indian version of Health Assessment Questionnaire Score and Short Form 36 Physical Function Score in rheumatoid arthritis using Rasch analysis	Indian J Rheumatol	2008	3(2)	52-57
		Citera G, et al.	Validation and Crosscultural Adaptation of an Argentine Spanish Version of the Health Assessment Questionnaire Disability Index	J Clin Rheumatol	2004	10(3)	110-115
		Cole JC, et al.	Single-factor scoring validation for the Health Assessment Questionnaire-Disability Index (HAQ-DI) in patients with systemic sclerosis and comparison with early rheumatoid arthritis patients	Qual Life Res	2006	15(8)	383-1394
		Cole JC, et al.	Validation of Single-Factor Structure and Scoring Protocol for the Health Assessment Questionnaire-Disability Index	Arthritis Rheum	2005	53(4)	536-542
		da Mota Falcão D, et al.	Translation and cultural adaptation of quality of life questionnaires: an evaluation of methodology	J Rheumatol	2003	30(2)	379-385
		Durán J, et al.	Evaluation of the health assessment questionnaire disability index in chilean patients with rheumatoid arthritis [Evaluación del health asessment questionnaire disability index (Haqdi) en pacientes chilenos con artritis reumatoide]	Revista Medica de Chile	2019	147(5)	612-617
		Duval A, et al.	Cross-cultural adaptation and validation of the French version of the Rheumatoid and Arthritis Outcome Score (RAOS)	Clin Exp Rheumatol	2010	28(6)	806-812
		El Meidany YM, et al.	Cross-cultural adaptation and validation of an Arabic Health Assessment Questionnaire for use in rheumatoid arthritis patients	Joint Bone Spine	2003	70(3)	195-202
		El Meidany YM, et al.	Evaluating changes in health status: sensitivity to change of the modified Arabic health assessment questionnaire in patients with rheumatoid arthritis	Joint Bone Spine	2003	70(6)	509-514
		Ellegaard K, et al.	Ultrasound Doppler measurements predict success of treatment with anti-TNF-a drug in patients with rheumatoid arthritis: a prospective cohort study	Rheumatology	2011	50(3)	506-512
		Fitzpatrick R, et al.	Importance of sensitivity to change as a criterion for selecting health status measures	Qual Health Care	1992	1(2)	89-93
		Fries J, et al.	The PROMIS of Better Outcome Assessment: Responsiveness, Floor and Ceiling Effects, and Internet Administration	J Rheumatol	2011	38(8)	759-1764
		Fries JF, et al.	Improved responsiveness and reduced sample size requirements of PROMIS physical function scales with item response theory	Arthritis Res Ther	2011	13(5)	R147
		Hussein H, et al.	Cross-cultural adaptation and validation of the Malay health assessment questionnaire for use in rheumatoid arthritis	Int J Rheum Dis	2008	11(3)	237-240
		Kumar A, et al.	Validation of an Indian version of the Health Assessment Questionnaire in patients with rheumatoid arthritis	Rheumatology	2002	41(12)	457-1459
		Linde L, et al.	Health-Related Quality of Life: Validity, Reliability, and Responsiveness of SF-36, EQ-15D, EQ-5D, RAQoL, and HAQ in Patients with Rheumatoid Arthritis	J Rheumatol	2008	35(8)	528-1537
		Marra CA, et al.	A comparison of generic, indirect utility measures (the HUI2, HUI3, SF-6D, and the EQ-5D) and disease-specific instruments (the RAQoL and the HAQ) in rheumatoid arthritis	Soc Sci Med	2005	60(7)	571-1582
		Marra CA, et al.	Are indirect utility measures reliable and responsive in rheumatoid arthritis patients?	Qual Life Res	2005	14(5)	333-1344
		Matsuda Y, et al.	Validation of a Japanese Version of the Stanford Health Assessment Questionnaire in 3,763 Patients With Rheumatoid Arthritis	Arthritis Rheum	2003	49(6)	784-788
		Minnock P, et al.	Fatigue is a reliable, sensitive and unique outcome measure in rheumatoid arthritis	Rheumatology	2009	48(12)	533-1536
		Osiri M, et al.	Comprehensibility, reliability, validity, and responsiveness of the Thai version of the Health Assessment Questionnaire in Thai patients with rheumatoid arthritis	Arthritis Res Ther	2009	11(4)	R129
		Osiri M, et al.	Evaluation of functional ability of Thai patients with rheumatoid arthritis by the use of a Thai version of the Health Assessment Questionnaire	Rheumatology	2001	40(5)	555-558
		Pope JE, et al.	The Minimally Important Difference for the Health Assessment Questionnaire in Rheumatoid Arthritis Clinical Practice Is Smaller Than in Randomized Controlled Trials	J Rheumatol	2009	36(2)	254-259
		Ripat J, et al.	A comparison of the Canadian Occupational Performance Measure and the Health Assessment Questionnaire	Can J Occup Ther	2001	68(4)	247-253
		Rohekar G & Pope J	Test-Retest Reliability of Patient Global Assessment and Physician Global Assessment in Rheumatoid Arthritis	J Rheumatol	2009	36(10)	178-2182
		Sahin F, et al.	Comparison of functional disability scales and their relevance to radiological progression in patients with rheumatoid arthritis in remission	Clin Exp Rheumatol	2006	24(5)	540-545
		Salaffi F, et al.	Measuring functional disability in early rheumatoid arthritis: the validity, reliability and responsiveness of the Recent-Onset Arthritis Disability (ROAD) index	Clin Exp Rheumatol	2005	3(5 Suppl 39)	S31-42
		Schefte DB & Hetland ML	An open-source, self-explanatory touch screen in routine care. Validity of filling in the Bath measures on Ankylosing Spondylitis Disease Activity Index, Function Index, the Health Assessment Questionnaire and Visual Analogue Scales in comparison with	Rheumatology	2010	49(1)	99-104
		Scott DL & Strand V	The effects of disease-modifying anti-rheumatic drugs on the Health Assessment Questionnaire score. Lessons from leflunomide clinical trials database	Rheumatology	2002	41(8)	899-909
		Seror R, et al.	Measure of function in rheumatoid arthritis: individualised or classical scales?	Ann Rheum Dis	2010	69(1)	97-101
		Tammaru M, et al.	The adaptation, reliability and validity testing of the Estonian version of the Health Assessment Questionnaire's Disability Index	Rheumatol Int	2007	28(1)	51-59
		Taylor WJ & Mcpherson KM	Using Rasch Analysis to Compare the Psychometric Properties of the Short Form 36 Physical Function Score and the Health Assessment Questionnaire Disability Index in Patients With Psoriatic Arthritis and Rheumatoid Arthritis	Arthritis Rheum	2007	57(5)	723-729
		Taylor WJ, et al.	Quality of life of people with rheumatoid arthritis as measured by the World Health Organization Quality of Life Instrument, short form (WHOQOL-BREF): score distributions and psychometric properties.	Arthritis Rheum	2004	51(3)	350-357
		ten Klooster PM, et al.	Rasch Analysis of the Dutch Health Assessment Questionnaire Disability Index and the Health Assessment Questionnaire II in Patients With Rheumatoid Arthritis	Arthritis Rheum	2008	59(12)	721-1728
		Thorsen H, et al.	Adaptation into Danish of the Stanford Health Assessment Questionnaire (HAQ) and the Rheumatoid Arthritis Quality of Life Scale (RAQoL)	Scand J Rheumatol	2001	30(2)	103-109

		Uhlig T, et al.	Comparison of the Health Assessment Questionnaire (HAQ) and the modified HAQ (MHAQ) in patients with rheumatoid arthritis	Rheumatology	2006	45(4)	454-458
		Vaidya B, et al.	Translation, cross-cultural adaptation and validation of Nepali version of Health Assessment Questionnaire-Disability Index in rheumatoid arthritis patients from Nepal	Int J Rheum Dis	2019	22(10)	871-1876
		Van Groen MM, et al.	Application of the health assessment questionnaire disability index to various rheumatic diseases	Qual Life Res	2010	19(9)	255-1263
		Veehof MM, et al.	Comparison of Internal and External Responsiveness of the Generic Medical Outcome Study Short Form-36 (SF-36) with Disease-specific Measures in Rheumatoid Arthritis	J Rheumatol	2008	35(4)	610-617
		Verhoeven AC, et al.	Responsiveness of the core set, response criteria, and utilities in early rheumatoid arthritis	Ann Rheum Dis	2000	59(12)	966-974
		Vermeulen HM, et al.	Responsiveness of the shoulder function assessment scale in patients with rheumatoid arthritis	Ann Rheum Dis	2006	65(2)	239-241
		Weisscher N, et al.	The Academic Medical Center Linear Disability Score Item Bank: Psychometric Properties of a New Generic Disability Measure in Rheumatoid Arthritis	J Rheumatol	2007	34(6)	222-1228
		Wells G, et al.	Responsiveness of patient reported outcomes including fatigue, sleep quality, activity limitation, and quality of life following treatment with abatacept for rheumatoid arthritis	Ann Rheum Dis	2008	67(2)	260-265
		Wikström I, et al.	Reliability, validity and responsiveness of a new leisure index: The Patient-Specific Leisure Scale (PSLS)	Musculoskelet Care	2009	7(3)	178-193
		Wolfe F	Which HAQ is best? A comparison of the HAQ, MHAQ and RA-HAQ, a difficult 8 item HAQ (DHAQ), and a rescored 20 item HAQ (HAQ20): analyses in 2,491 rheumatoid arthritis patients following leflunomide initiation	J Rheumatol	2001	28(5)	982-989
**72**	**HAQ-II**	Oude Voshaar MA, et al.	Crosscultural Measurement Equivalence of the Health Assessment Questionnaire II	Arthritis Care Res	2013	65(6)	000-1004
		ten Klooster PM, et al.	Rasch Analysis of the Dutch Health Assessment Questionnaire Disability Index and the Health Assessment Questionnaire II in Patients With Rheumatoid Arthritis	Arthritis Rheum	2008	59(12)	721-1728
		Wolfe F, et al.	Development and Validation of the Health Assessment Questionnaire II A Revised Version of the Health Assessment Questionnaire	Arthritis Rheum	2004	50(10)	296-3305
**73**	**JPBA-S**	Hammond A & Lincoln N	Development of the Joint Protection Behavior Assessment	Arthritis Care Res	1999	12(3)	200-207
		Niedermann A, et al.	Development and Validation of a German Version of the Joint Protection Behavior Assessment in Patients With Rheumatoid Arthritis	Arthritis Rheum	2007	57(2)	249-255
		Tonga E, et al.	Development and reliability of a Turkish version of the Short Form-Joint Protection Behavior Assessment (JPBA-S)	J Hand Ther	2016	29(3)	275-280
**74**	**MACTAR**	Brigitte M, et al.	A study compared nine patient-specific indices for musculoskeletal disorders	J Clin Epidemiol	2005	58(8)	791-801
		Brodin N, et al.	The McMaster Toronto Arthritis patient preference questionnaire (MACTAR): a methodological study of reliability and minimal detectable change after a 6 week-period of acupuncture treatmentin patients with rheumatoid arthritis	BMC Res Notes	2017	10	687
		Munters LA, et al.	Disabilities of importance for patients to improve – using a patient preference tool in rheumatoid arthritis	Disabil Rehabil	2014	36(21)	762-1767
		Verhoeven AC, et al.	Validity of the MACTAR questionnaire as a functional index in a rheumatoid arthritis clinical trial. The McMaster Toronto Arthritis	J Rheumatol	2000	27(12)	801-2809
**75**	**MAL**	Goodacre L, et al.	Development and validation of a patient-centred Measure of Activity Limitation (MAL) in rheumatoid arthritis	Rheumatology	2007	46(4)	703-708
**76**	**MAP-Hand**	Paulsen T, et al.	Development and Psychometric Testing of the Patient Reported Measure of Activity Performance of the Hand (MAP-HAND) in Rheumatoid Arthritis	J Rehabil Med	2010	42(7)	636-644
		Prior Y, et al.	Measure of activity performance of the hand (MAP-Hand) questionnaire: linguistic validation, cultural adaptation and psychometric testing in people with rheumatoid arthritis in the UK	BMC Musculoskelet Disord	2018	19(1)	275
**77**	**MDHAQ**	Arkela-Kautiainen M, et al.	Evaluation of the MDHAQ in Finnish patients with RA [corrected]	J Rheumatol	2005	32(8)	426-1431
		Josefsson KA, et al.	Swedish version of the multi dimensional health assessment questionnaire – translation and psychometric evaluation	BMC Musculoskelet Disord	2013	14	178
		Song Y, et al.	Multi-dimensional health assessment questionnaire in China: reliability, validity and clinical value in patients with rheumatoid arthritis.	PLoS One	2014	9(5)	e97952
		Yokogawa N, et al.	Validation of RAPID3 using a Japanese version of Multidimensional Health Assessment Questionnaire with Japanese rheumatoid arthritis patients: characteristics of RAPID3 compared to DAS28 and CDAI	Mod Rheumatol	2015	25(2)	264-269
**78**	**MHAQ**	Arvidson NG, et al.	Simple function tests, but not themodified HAQ, correlate with radiological joint damage in rheumatoid arthritis	Scand J Rheumatol	2002	31(3)	146-150
		Benitha R & Tikly M	Functional disability and health-related quality of life in South Africans with rheumatoid arthritis and systemic lupus erythematosus	Clin Rheumatol	2007	26(1)	24-29
		Escalante A, et al.	Cross-Cultural Equivalence of a Brief Helplessness Scale for Spanish-Speaking Rheumatology Patients in the United States	Arthritis Care Res	1999	12(5)	341-350
		Hagen KB, et al.	The responsiveness of health status measures in patients with rheumatoid arthritis: Comparison of disease-specific and generic instruments	J Rheumatol	1999	26(7)	474-1480
		Johnson AH, et al.	The mechanical joint score: a new clinical index of joint damage in rheumatoid arthritis	Rheumatology	2002	41(2)	189-195
		Kvien TK, et al.	Performance of health status measures with a pen based personal digital assistant	Ann Rheum Dis	2005	64(10)	480-1484
		Martin M, et al.	Item response theory methods can improve the measurement of physical function by combining the modified health assessment questionnaire and the SF-36 physical function scale	Qual Life Res	2007	16(4)	647-660
		Russell AS, et al.	The responsiveness of generic health status measures as assessed in patients with rheumatoid arthritis receiving infliximab	J Rheumatol	2003	30(5)	941-947
		Uhlig t, et al.	Comparison of the Health Assessment Questionnaire (HAQ) and the modified HAQ (MHAQ) in patients with rheumatoid arthritis	Rheumatology	2006	45(4)	454-458
		Uhlig t, et al.	Responsiveness of the International Classification of Functioning, Disability and Health (ICF) Core Set for rheumatoid arthritis	Ann Rheum Dis	2009	68(6)	879-884
		Wolfe F	Which HAQ is best? A comparison of the HAQ, MHAQ and RA-HAQ, a difficult 8 item HAQ (DHAQ), and a rescored 20 item HAQ (HAQ20): analyses in 2,491 rheumatoid arthritis patients following leflunomide initiation	J Rheumatol	2001	28(5)	982-989
**79**	**PI-HAQ**	Hewlett S, et al.	Measuring the meaning of disability in rheumatoid arthritis: the Personal Impact Health Assessment Questionnaire (PI HAQ)	Ann Rheum Dis	2002	61(11)	986-993
		Shakibi MR, et al.	Validation of the Personal Impact Health Assessment Questionnaire in Patients with Rheumatoid Arthritis in Kerman, Iran	Turk J Rheumatol	2012	27(2)	121-127
**80**	**PROMIS-PF 10a-SF**	Fries JF, et al.	Improved responsiveness and reduced sample size requirements of PROMIS physical function scales with item response theory	Arthritis Res Ther	2011	13(5)	R147
		Izadi Z, et al.	Effects of language, insurance and race/ethnicity on measurement properties of the PROMIS Physical Function Short Form 10a in rheumatoid arthritis	Arthritis Care Res (Hoboken)	2019	71(7)	925-935
		Mahmoud GA, et al.	Cross cultural adaptation and validation of an Arabic version of selected PROMIS measures for use in rheumatoid arthritis patients	The Egyptian Rheumatologist	2019	41(3)	177-182
		Schalet B, et al.	Validity of PROMIS physical function measured in diverse clinical samples	J Clin Epidemiol	2016	73	112-118
		Wahl E, et al.	Validity and Responsiveness of a 10-Item Patient-Reported Measure of Physical Function in a Rheumatoid Arthritis Clinic Population	Arthritis Care Res	2017	69(3)	338-346
**81**	**PROMIS-PF 20-SF**	Fries JF, et al.	Improved responsiveness and reduced sample size requirements of PROMIS physical function scales with item response theory	Arthritis Res Ther	2011	13(5)	R147
		Hays RD, et al.	Responsiveness and minimally important difference for the Patient-Reported Outcomes Measurement Information System (PROMIS) 20-item physical functioning short form in a prospective observational study of rheumatoid arthritis	Ann Rheum Dis	2015	74(1)	104-107
		Oude Voshaar MA, et al.	Validity and measurement precision of the PROMIS physical function item bank and a content validity driven 20-item short form in rheumatoid arthritis compared with traditional measures	Rheumatology	2015	54(12)	221-2229
**82**	**PS-ADL**	Archenholtz B & Dellhag B	Validity and reliability of the instrument Performance and Satisfaction in Activities of Daily Living (PS-ADL) and its clinical applicability to adults with rheumatoid arthritis	Scand J Occup Ther	2008	15(1)	13-22
**83**	**QuickDASH**	Hammond A, et al.	Linguistic validation, validity and reliability of the British English versions of the Disabilities of the Arm, Shoulder and Hand (DASH) questionnaire and QuickDASH in people with rheumatoid arthritis	BMC Musculoskelet Disord	2018	19	118
		Prodinger B, et al.	Revisiting the disabilities of the arm, shoulder and hand (DASH) and QuickDASH in rheumatoid arthritis	BMC Musculoskelet Disord	2019	20	41
**84**	**RHDA**	Guermazi M, et al.	Development and validation of an Arabic rheumatoid hand disability scale	Disabil Rehabil	2004	26(11)	655-661
**85**	**PSLS**	Wikström I, et al.	Reliability, validity andresponsiveness of a new leisure index: The Patient-Specific Leisure Scale (PSLS)	Musculoskelet Care	2009	7(3)	178-193
**86**	**5T-PROs**	Salaffi F, et al.	The Patient-Reported Outcomes Thermometer-5-Item Scale (5T-PROs): Validation of a New Tool for the Quick Assessment of Overall Health Status in Painful Rheumatic Diseases	Pain Res Manag	2018	2018	3496846
**87**	**CSHQ-RA-SF**	Chiou CF, et al.	Development and Validation of Cedars-Sinai Health-Related Quality of Life in Rheumatoid Arthritis (CSHQ-RA) Short Form Instrument	Arthritis Rheum	2004	51(3)	358-364
		Chiou CF, et al.	Revalidation of the Original Cedars-Sinai Health-Related Quality of Life in Rheumatoid Arthritis Questionnaire	J Rheumatol	2006	33(2)	256-262
**88**	**FLARE-RA**	Barroso N, et al.	The American English version of the validated French Flare Assessment in RA Questionnaire (FLARE-RA)	Clin Rheumatol	2020	39(1)	189-199
		Berthelot JM, et al.	A tool to identify recent or present rheumatoid arthritis flare from both patient and physician perspectives: The 'FLARE' instrument	Ann Rheum Dis	2012	71(7)	110-1116
		de Thurah A, et al.	Patient self-assessment of flare in rheumatoid arthritis: criterion and concurrent validity of the Flare instrument	Clin Rheumatol	2016	35(2)	467-471
		Fautrel B, et al.	Validation of FLARE-RA, a Self-Administered Tool to Detect Recent or Current Rheumatoid Arthritis Flare	Arthritis Rheumatol	2017	69(2)	309-319
		Maribo T, et al.	Patient-self assessment of flare in rheumatoid arthritis:translation and reliability of the Flare instrument	Clin Rheumatol	2016	35(4)	053-1058
		Myasoedova, E	Definition and construct validation of clinically relevant cutoffs on the Flare Assessment in Rheumatoid Arthritis (FLARE-RA) questionnaire	Semin Arthritis Rheum	2020	50	261-265
**89**	**M-SACRAH**	Sautner J, et al.	Development of the M-SACRAH, a modified shortened version of SACRAH (Score for assessment and Qauntification of Chronic Rheumatoid Affections of the Hands)	Rheumatology	2004	43(11)	409-1413
		Singh H, et al.	Assessment of hand functions in rheumatoid arthritis using SF-SACRAH (short form score for the assessment and quantification of chronic rheumatoid affections of the hands) and its correlation to disease activity	Rheumatol Int	2012	32(11)	413-3419
**90**	**MSK-HQ**	Norton S, et al.	Validation of the Musculoskeletal Health Questionnaire in Inflammatory Arthritis: A Psychometric Evaluation	Rheumatology (Oxford)	2019	58(1)	45-51
**91**	**RA-FQ**	Bartlett SJ, et al.	Content and Construct Validity, Reliability, and Responsiveness of the Rheumatoid Arthritis Flare Questionnaire: OMERACT 2016 Workshop Report	J Rheumatol	2017	44(10)	536-1543
**92**	**RAID**	Dougados M, et al.	Defining cut-off values for disease activity states and improvement scores for patient-reported outcomes: the example of the Rheumatoid Arthritis Impact of Disease (RAID)	Arthritis Res Ther	2012	14(3)	R129
		Fautrel B, et al.	Validation of FLARE-RA, a Self-Administered Tool to Detect Recent or Current Rheumatoid Arthritis Flare	Arthritis Rheum	2017	69(2)	309-319
		Ferreira RJO, et al.	The Portuguese Rheumatoid Arthritis Impact of Disease (RAID) score and its measurement equivalence in three countries: validation study using Rasch Models	Qual Life Res	2018	27(11)	909-2921
		Gossec L, et al.	Elaboration of the preliminary Rheumatoid Arthritis Impact of Disease (RAID) score: a EULAR initiative	Ann Rheum Dis	2009	68(11)	680-1685
		Gossec L, et al.	Finalisation and validation of the rheumatoid arthritis impact of disease score, a patient-derived composite measure of impact of rheumatoid arthritis: a EULAR initiative	Ann Rheum Dis	2011	70(6)	935-942
		Heiberg T, et al.	Performance of the Rheumatoid Arthritis Impact of Disease (RAID) score in relation to other patient-reported outcomes in a register of patients with rheumatoid arthritis	Ann Rheum Dis	2011	70(6)	080-1082
		Hewlett S, et al.	The revised Bristol Rheumatoid Arthritis Fatigue measures and the Rheumatoid Arthritis Impact of Disease scale: validation in six countries	Rheumatology	2018	57(2)	300-308
		Holten K, et al.	Comparative analyses of responsiveness between the Rheumatoid Arthritis Impact of Disease score, other patient-reported outcomes and disease activity measures: secondary analyses from the ARCTIC study	RMD Open	2018	4(2)	e000754
		Oude Voshaar MAH, et al.	Psychometric properties and cross-language equivalence of the revised Bristol Rheumatoid Arthritis Fatigue and the Rheumatoid Arthritis Impact of Disease scales in rheumatoid arthritis	Quality of Life Research	2019	28(9)	543-2552
		Salaffi F, et al.	Validity of the rheumatoid arthritis impact of disease (RAID) score and definition of cut-off points for disease activity states in a population-based European cohort of patients with rheumatoid arthritis	Joint Bone Spine	2018	85(3)	317-322
		ter Wee MM, et al.	Content validity of the Dutch Rheumatoid Arthritis Impact of Disease (RAID) score: results of focus group discussions in established rheumatoid arthritis patients and comparison with the International Classification of Functioning, Disability and Health c	Arthritis Res Ther	2016	18	22

**93**	**RAPID3**	Boone NW, et al.	Routine Assessment of Patient Index Data 3 (RAPID3) alone is insufficient to monitor disease activity in rheumatoid arthritis in clinical practice	RMD Open	2019	5(2)	e001050
		Bossert M, et al.	Evaluation of self-report questionnaires for assessing rheumatoid arthritis activity: a cross-sectional study of RAPID3 and RADAI5 and flare detection in 200 patients	Joint Bone Spine	2012	79(1)	57-62
		Horta-Baas G, et al.	Evaluation of the activity of rheumatoid arthritis in clinical practice. Agreement between self-rated clinimetric evaluation and clinical evaluation with activity indexes: DAS28, CDAI and SDAI	Med Clin (Barc)	2017	149(7)	293-299
		Leeb BF, et al.	A comparison of patient questionnaires and composite indexes in routine care of rheumatoid arthritis patients	Joint Bone Spine	2009	76(6)	658-64
		Pincus T, et al.	RAPID3, an index to assess and monitor patients with rheumatoid arthritis, without formal joint counts: similar results to DAS28 and CDAI in clinical trials and clinical care	Rheum Dis Clin North Am	2009	35(4)	773-778
		Salaffi F, et al.	New Indices study group. The comparative responsiveness of the patient self-report questionnaires and composite disease indices for assessing rheumatoid arthritis activity in routine care	Clin Exp Rheumatol	2012	30(6)	912-921
		Uhlig T, et al.	Test-retest reliability of disease activity core set measures and indices in rheumatoid arthritis	Ann Rheum Dis	2009	68(6)	972-975
		Yokogawa N, et al.	Validation of RAPID3 using a Japanese version of Multidimensional Health Assessment Questionnaire with Japanese rheumatoid arthritis patients: characteristics of RAPID3 compared to DAS28 and CDAI	Mod Rheumatol	2015	25(2)	264-269
**94**	**SF-SACRAH**	Rintelen B, et al.	A tool for the assessment of hand involvement in rheumatic disorders in daily routine e the SF-SACRAH (short form score for the assessment and quantification of chronic rheumatic affections of the hands)	Osteoarthritis Cartilage	2009	17(1)	59-63
		Stummer U, et al.	The Short Form Score for the Assessment and Quantification of Chronic Rheumatic Affections of the Hands in Daily Clinical Routines—Its Sensitivity to Change and Preliminary Patient Relevant Variation Values: A Pilot Study	Front Med (Lausanne)	2017	4	6
**95**	**SMFA**	Wollmerstedt N, et al.	Reliability, validity and responsiveness of the German Short Musculoskeletal Function Assessment Questionnaire in patients undergoing surgical or conservative inpatient treatment	Qual Life Res	2006	15(7)	233-1241
**96**	**S-VLA**	Katz PP, et al.	Development and Validation of a Short Form of the Valued Life Activities Disability Questionnaire for Rheumatoid Arthritis	Arthritis Care Res	2011	63(12)	664-1671
**97**	**VLA**	Björk M, et al.	Validation and internal consistency of the Swedish version of the Valued Life Activities scale	Clin Rehabil	2016	30(12)	211-1219
**98**	**CQ-Index RA**	Zuidgeest M, et al.	Measuring patients' experiences with rheumatic care: the consumer quality index rheumatoid arthritis.	Rheumatol Int	2009	30(2)	159-167
**99**	**ISMI-RA**	Corker E, et al.	Internalised stigma in people with rheumatoid arthritis: a cross sectional study to establish the psychometric properties of the ISMI-RA	BMC Musculoskelet Disord	2016	17	244
**100**	**PCCoc/rheum**	Bala SV, et al.	Measuring person-centred care in nurse-led outpatient rheumatology clinics	Musculoskeletal Care	2018	16(2)	296-304
		Bala SV, et al.	Person-centred care in nurse-led outpatient rheumatology clinics: Conceptualization and initial development of a measurement instrument.	Musculoskeletal Care	2018	16(2)	287-295
**101**	**PHCPCS**	Salt E, et al.	Development of a quality of patient–health care provider communication scale from the perspective of patients with rheumatoid arthritis	Chronic Illn	2013	9(2)	103-115
**102**	**AHI**	Hagglund HJ, et al.	Discriminant and convergent validity of self-report measures of affective distress in patients with RA	J Rheumatol	1989	16(11)	1428-32
		Lindroth Y, et al.	Learned Helplessness and its Correlation to Impairment, Pain,Anxiety and Depression in Rheumatoid Arthritis	Scand J Rheumatol	1994	23(6)	299-304
		Nicasso PM, et al.	The measurement of helplessness in rheumatoid arthritis. The development of the arthritis helplessness index	J Rheumatol	1985	12(3)	462-467
		Stein MJ, et al.	Factor structure of the Arthritis Helplessness Index	J Rheumatol	1988	15(3)	427-432
**103**	**ASES**	Primdahl J, et al.	Self-efficacy in Rheumatoid Arthritis: Translation and Test of Validity, Reliability and Sensitivity of the Danish Version of the Rheumatoid Arthritis Self-Efficacy Questionnaire (RASE)	Musculoskelet Care	2010	8(3)	123-135
**104**	**ASES-8**	Gao L, et al.	Psychometric properties of the Chinese version of Arthritis Self-Efficacy Scale-8 (ASES-8) in a rheumatoid arthritis population	Rheumatol Int	2017	37(5)	751-756
		Silva RVTD, et al.	Translation to Brazilian Portuguese, cultural adaptation and psychometric properties of 8-item Arthritis Self-Efficacy Scale (ASES-8)	Sao Paulo Med J	2019	137(1)	6-12
**105**	**BRCS**	Sinclair VG & Wallston KA	The Development and Psychometric Evaluation of the Brief Resilient Coping Scale	Assessment	2004	11(1)	94-101
**106**	**ESES**	Nessen T, et al.	The Swedish Exercise Self-Efficacy Scale (ESES-S): reliability and validity in a rheumatoid arthritis population	Disabil Rehabil	2015	37(2)	130-2134
**107**	**JP-SES**	Niedermann K, et al.	Development and psychometric properties of a joint protectionself-efficacy scale	Scand J Occup Ther	2011	18(2)	143-152
		Zhao WH, et al.	Validation of the Chinese version of joint protection self-efficacy scale in patients with rheumatoid arthritis	Clin Rheumatol	2019	38(8)	119-2127
**108**	**RAEH**	Contreras-Yáñez I, et al.	Psychometric Validation of an Empowerment Scale for Spanish-speaking Patients With Rheumatoid Arthritis	Arthritis Res Ther	2018	20(1)	244
**109**	**RAI**	Callahan LF, et al.	Further analysis of learned helplessness in rheumatoid arthritis using a "Rheumatology Attitudes Index"	J Rheumatol	1988	15(3)	418-426
**110**	**RASE**	Brady TJ	Measures of self-efficacy: Arthritis Self-Efficacy Scale (ASES), Arthritis Self-Efficacy Scale-8 Item (ASES-8), Children's Arthritis Self-Efficacy Scale (CASE), Chronic Disease Self-Efficacy Scale (CDSES), Parent's Arthritis Self-Efficacy Scale (PASE), and Rheumato	Arthritis Care Res	2011	63(S11)	473-S485
		Garratt AM, et al.	Measurement properties of instruments assessing self-efficacy in patients with rheumatic diseases	Rheumatology	2014	53(7)	161-1171
		Hewlett S, et al.	Development and validation of a self efficacy scale for use in British patients with RA	Rheumatology	2001	40(11)	221-1230
		Hewlett S, et al.	Sensitivity to change of the Rheumatoid Arthritis Self-Efficacy scale (RASE) and predictors of change in self-efficacy	Musculoskelet Care	2008	6(1)	49-67
		Primdahl J, et al.	Self-efficacy in Rheumatoid Arthritis: Translation and Test of Validity, Reliability and Sensitivity of the Danish Version of the Rheumatoid Arthritis Self-Efficacy Questionnaire (RASE)	Musculoskelet Care	2010	8(3)	123-135
**111**	**RPQ**	Contreras-Yáñez , et al.	Validation of a risk perception questionnaire developed for patients with rheumatoid arthritis	PLoS One	2019	14(7)	ꞏ0219921
**112**	**SCBS**	Nadrian H, et al.	Development and psychometric properties of a self-care behaviors scale (SCBS) among patients with rheumatoid arthritis [published correction appears in BMC Rheumatol. 2019 Aug 20;3:35]	BMC Rheumatol	2019	3	4
**113**	**SIAQ**	Keininger D & Coteur G	Assessment of self-injection experience in patients with rheumatoid arthritis: psychometricvalidation of the Self-Injection Assessment Questionnaire (SIAQ)	Health Qual Life Outcomes	2011	9	2
**114**	**VPMI**	Brown GK & Nicassio PM	Development of a questionnaire for the assessment of active and passive coping strategies in chronic pain patients	Pain	1987	31(1)	53-64
		Smith CA, et al.	Beyond good and bad coping: A multidimensional examination of coping with pain in persons with rheumatoid arthritis	Ann Behav Med	1997	19(1)	11-21
**115**	**ENAT**	Cruz A, et al.	Cross-cultural validation of the portuguese version of the Educational Needs Assessment Tool (PORTENAT)	Acta Reumatol Port	2015	40(3)	242-253
		Drăgoi RG, et al.	Patient education, disease activity and physical function: can we be more targeted? A cross sectional study among people with rheumatoid arthritis, psoriatic arthritis and hand osteoarthritis	Arthritis Res Ther	2013	15(5)	R156
		Ndosi M, et al.	Cross-cultural validation of the Educational Needs Assessment Tool in RA in 7 European countries	BMC Musculoskelet Disord	2011	12	110
		Sierakowska M, et al.	Developing the Polish Educational Needs Assessment Tool (Pol-ENAT) in rheumatoid arthritis and systemic sclerosis: a cross-cultural validation study using Rasch analysis	Qual Life Res	2015	24(3)	721-733
**116**	**HDFQ-RA**	Frayssac T, et al.	Translation and adaptation of the French version of the Heart Disease Fact Questionnaire – Rheumatoid Arthritis (HDFQ-RA 1&2)	Joint Bone Spine	2017	84(6)	693-698
		John H, et al.	Development and initial validation of a heart disease knowledge questionnaire for people with rheumatoidarthritis	Patient Educ Couns	2009	77(1)	136-143
**117**	**RA-KAS**	Naqvi AA, et al.	Development and validation of a novel rheumatoid arthritis knowledge assessment scale in Pakistani patients with rheumatoid arthritis	Int J Rheum Dis	2019	22(11)	031-2044
**118**	**PROMIS-GH v1.1-S**	Wohlfahrt A, et al.	Responsiveness of Patient-Reported Outcomes Measurement Information System Measures in Rheumatoid Arthritis Patients Starting or Switching a Disease-Modifying Antirheumatic Drug	Arthritis Care Res (Hoboken)	2019	71(4)	521-529
**119**	**QOL-RA**	Danao LL, et al.	An English and Spanish Quality of Life Measure for Rheumatoid Arthritis	Arthritis Care Res	2001	45(2)	167-173
		Sizova LV	Validation of the Russian version of the Quality of Life-Rheumatoid Arthritis Scale (QOL-RA Scale)	Adv Med Sci	2009	54(1)	27-31
**120**	**RAQoL**	de Jong Z, et al.	The reliability and construct validity of the RAQoL: a rheumatoid arthritis-specific quality of life instrument	Br J Rheumatol	1997	36(8)	878-883
		Duarte C, et al.	Validation of Rheumatoid Arthritis Quality of Life (RAQoL) Questionnaire into Portuguese Language [Validation of Rheumatoid Arthritis Quality of Life (RAQoL) Questionnaire into Portuguese Language]	Acta Reumatol Port	2019	44(1)	65-70
		Greenwood MC, et al.	A simple extension to the Rheumatoid Arthritis Quality of Life Questionnaire (RAQol) to explore individual patient concerns and monitor group outcome in clinical practice	Rheumatology	2006	45(1)	61-65
		Greenwood MC, et al.	Touch-screen computer systems in the rheumatology clinic offer a reliable and user-friendly means of collecting quality-of-life and outcome data from patients with rheumatoid arthritis	Rheumatology	2006	45(1)	66-71
		Heaney A, et al.	A Review of the Psychometric Properties and Use of the Rheumatoid Arthritis Quality of Life Questionnaire (RAQoL) in Clinical Research	Curr Rheumatol Rev	2017	13(3)	197-205
		Hedin PJ, et al.	The Rheumatoid Arthritis Quality of Life (RAQoL) for Sweden: adaptation and validation	Scand J Rheumatol	2006	35(2)	117-123
		Linde L, et al.	Health-Related Quality of Life: Validity, Reliability, and Responsiveness of SF-36, EQ-15D, EQ-5D, RAQoL, and HAQ in Patients with Rheumatoid Arthritis	J Rheumatol	2008	35(8)	528-1537
		Marra CA, et al.	A comparison of generic, indirect utility measures (the HUI2, HUI3, SF-6D, and the EQ-5D) and disease-specific instruments (the RAQoL and the HAQ) in rheumatoid arthritis	Soc Sci Med	2005	60(7)	571-1582
		Swinkels RA, et al.	Which are the best instruments for measuring disabilities in gait and gait-related activities in patients with rheumatic disorders	Clin Exp Rheumatol	2004	22(1)	25-33
		Tijhuis GJ, et al.	The validity of the Rheumatoid Arthritis Quality of Life (RAQoL) questionnaire	Rheumatology	2001	40(10)	112-1119
		Waimann CA, et al.	Quality of life of patients with rheumatoid arthritis in Argentina: reliability, validity, and sensitivity to change of a Spanish version of the Rheumatoid Arthritis Quality of Life questionnaire	Clin Rheumatol	2012	31(7)	065-1071
		Whalley D, et al.	Quality of life in rheumatoid arthritis	Br J Rheumatol	1997	36(8)	884-888
		Wilburn J, et al.	Further international adaptation and validation of the Rheumatoid Arthritis Quality of Life (RAQoL) questionnaire	Rheumatol Int	2015	35(4)	669-675
		Zlatkovic-Svenda M, et al.	Adaptation and validation of the Rheumatoid Arthritis Quality of Life (RAQoL) questionnaire for use in Serbia	Rheumatol Int	2017	37(4)	641-646
**121**	**EQ-5D**	Adams R, et al.	Understanding the Relationship between the EQ-5D, SF-6D, HAQ and Disease Activity in Inflammatory Arthritis	Pharmacoeconomics	2010	28(6)	477-487
		Ariza-Ariza R, et al.	Assessing Utility Values in Rheumatoid Arthritis: A Comparison Between Time Trade-Off and the EuroQol	Arthritis Rheum	2006	55(5)	751-756
		Barton GR, et al.	A comparison of the performance of the EQ-5D and the SF-6D for individuals aged >or= 45 years	Health Econ	2008	17(7)	815-832
		Buitinga L, et al.	Comparative Responsiveness of the EuroQol-5D and Short Form 6D to Improvement in Patients With Rheumatoid Arthritis Treated With Tumor Necrosis Factor Blockers: Results of the DutchRheumatoid Arthritis Monitoring Registry	Arthritis Care Res	2012	64(6)	826-832
		Conner-Spady B & Suarez-Almazo	Variation in the estimation of quality-adjusted life-years by different preference-based instruments.	Med Care	2003	41(7)	791-801
		Dritsaki M, et al.	An empirical evaluation of the SF-12, SF-6D, EQ-5D and Michigan Hand Outcome Questionnaire in patients with rheumatoid arthritis of the hand	Health Qual Life Outcomes	2017	15(1)	20
		Ferreira LN, et al.	Comparing the Performance of the SF-6D and the EQ-5D in Different Patient Groups	Acta Med Port	2014	27(2)	236-245
		Gaujoux-Viala C, et al.	Comparison of the EQ-5D and the SF-6D Utility Measures in 813 Patients with Early Arthritis: Results from the ESPOIR Cohort	J Rheumatol	2011	38(8)	576-1584
		Groothuis-Oudshoorn CG, et al.	Development and validation of the Dutch version of the London Handicap Scale	J Back Musculoskelet Rehabil	2015	28(2)	401-408
		Harrison MJ, et al.	Exploring the validity of estimating EQ-5D and SF-6D utility values from the health assessment questionnaire in patients with inflammatory arthritis	Health Qual Life Outcomes	2010	8	21
		Harrison MJ, et al.	The comparative responsiveness of the EQ-5D and SF-6D to change in patients with inflammatory arthritis	Qual Life Res	2009	18(9)	195-1205
		Hu H, et al.	Psychometric validation of Chinese Health Assessment Questionnaire for use in rheumatoid arthritis patients in China	Int J Rheum Dis	2017	20(12)	987-1992
		Hurst NP, et al.	Measuring health-related quality of life in rheumatoid arthritis: validity, responsiveness and reliability of EuroQol (EQ-5D)	Br J Rheumatol	1997	36(5)	551-559
		Hurst NP, et al.	Validity of Euroqol--a generic health status instrument--in patients with rheumatoid arthritis. Economic and Health Outcomes Research Group	Br J Rheumatol	1994	33(7)	655-662

		Kaplan RM, et al.	Comparison of Measured Utility Scores and Imputes Scores From the SF 36 in Patients with Rheumatoid Arthritis	Med Care	2005	43(1)	79-87
		Linde L, et al.	Health-Related Quality of Life: Validity, Reliability, and Responsiveness of SF-36, EQ-15D, EQ-5D, RAQoL, and HAQ in Patients with Rheumatoid Arthritis	J Rheumatol	2008	35(8)	528-1537
		Luo N, et al.	A comparison of the EuroQol-5D and the Health Utilities Index mark 3 in patients with rheumatic disease.	J Rheumatol	2003	30(10)	268-2274
		Marra CA, et al.	A Comparison of Four Indirect Methods of Assessing Utility Values in Rheumatoid Arthritis	Med Care	2004	42(11)	125-1131
		Marra CA, et al.	A comparison of generic, indirect utility measures (the HUI2, HUI3, SF-6D, and the EQ-5D) and disease-specific instruments (the RAQoL and the HAQ) in rheumatoid arthritis	Soc Sci Med	2005	60(7)	571-1582
		Marra CA, et al.	Are indirect utility measures reliable and responsive in rheumatoid arthritis patients?	Qual Life Res	2005	14(5)	333-1344
		Russell AS, et al.	The responsiveness of generic health status measures as assessed in patients with rheumatoid arthritis receiving infliximab	J Rheumatol	2003	30(5)	941-947
		Salaffi F, et al.	A comparison of utility measures using EQ-5D and SF-6D preference-based generic instruments in patients with rheumatoid arthritis	Clin Exp Rheumatol	2011	29(4)	661-671
		Traki L, et al.	Responsiveness of the EuroQol EQ-5D and Hospital Anxiety and Depression Scale (HADS) in rheumatoid arthritis patients receiving tocilizumab	Clin Rheumatol	2014	33(8)	055-1060
**122**	**HUI-2**	Marra CA, et al.	A comparison of generic, indirect utility measures (the HUI2, HUI3, SF-6D, and the EQ-5D) and disease-specific instruments (the RAQoL and the HAQ) in rheumatoid arthritis	Soc Sci Med	2005	60(7)	571-1582
		Marra CA, et al.	Are indirect utility measures reliable and responsive in rheumatoid arthritis patients?	Qual Life Res	2005	14(5)	333-1344
**123**	**HUI-3**	Marra CA, et al.	A comparison of generic, indirect utility measures (the HUI2, HUI3, SF-6D, and the EQ-5D) and disease-specific instruments (the RAQoL and the HAQ) in rheumatoid arthritis	Soc Sci Med	2005	60(7)	571-1582
		Marra CA, et al.	Are indirect utility measures reliable and responsive in rheumatoid arthritis patients?	Qual Life Res	2005	14(5)	333-1344
**124**	**QWB**	Bombardier C & Raboud J	A comparison of health-related quality-of-life measures for rheumatoid arthritis research. The Auranofin Cooperating Group.	Control Clin Trials	1991	12(4 Suppl)	243-S256
**125**	**SF-6D**	Buitinga L, et al.	Comparative Responsiveness of the EuroQol-5D and Short Form 6D to Improvement in Patients With Rheumatoid Arthritis Treated With Tumor Necrosis Factor Blockers: Results of the DutchRheumatoid Arthritis Monitoring Registry	Arthritis Care Res	2012	64(6)	826-832
		Campolina AG, et al.	The SF-6D Brazil questionnaire: generation models and applications in health economics	Rev Assoc Med Bras	2010	56(4)	409-14
		Dritsaki M, et al.	An empirical evaluation of the SF-12, SF-6D, EQ-5D and Michigan Hand Outcome Questionnaire in patients with rheumatoid arthritis of the hand	Health Qual Life Outcomes	2017	15(1)	20
		Ferreira LN, et al.	Comparing the Performance of the SF-6D and the EQ-5D in Different Patient Groups	Acta Med Port	2014	27(2)	236-245
		Gaujoux-Viala C, et al.	Comparison of the EQ-5D and the SF-6D Utility Measures in 813 Patients with Early Arthritis: Results from the ESPOIR Cohort	J Rheumatol	2011	38(8)	576-1584
		Harrison MJ, et al.	The comparative responsiveness of the EQ-5D and SF-6D to change in patients with inflammatory arthritis	Qual Life Res	2009	18(9)	195-1205
		Harrison MJ, et al.	The Validity and Responsiveness of Generic Utility Measures in Rheumatoid Arthritis: A Review	J Rheumatol	2008	35(4)	592-602
		Marra CA, et al.	A comparison of generic, indirect utility measures (the HUI2, HUI3, SF-6D, and the EQ-5D) and disease-specific instruments (the RAQoL and the HAQ) in rheumatoid arthritis	Soc Sci Med	2005	60(7)	571-1582
		Marra CA, et al.	Are indirect utility measures reliable and responsive in rheumatoid arthritis patients?	Qual Life Res	2005	14(5)	333-1344
		Russell AS, et al.	The responsiveness of generic health status measures as assessed in patients with rheumatoid arthritis receiving infliximab	J Rheumatol	2003	30(5)	941-947
		Salaffi F, et al.	A comparison of utility measures using EQ-5D and SF-6D preference-based generic instruments in patients with rheumatoid arthritis	Clin Exp Rheumatol	2011	29(4)	661-671
