# Supplementary material for: Use and detailed metric properties of patient-reported outcome measures for rheumatoid arthritis: a systematic review covering two decades
Source: RMD Open. 2021 Aug 10;7(2):e001707. doi: 10.1136/rmdopen-2021-001707 (PMC8356163; doi:10.1136/rmdopen-2021-001707)
Supplement: Supplementary data [file rmdopen-2021-001707supp005.pdf]

	nemonic	Full Name
1	AQoL	Assessment of Quality of Life
2	BAQ	Body Awareness Questionnaire
3	BDI-II	Beck depression Inventory -II
4	BI	Barthel Index
5	CIJSS	Chronic Illness Job Strain Scale
6	DELAY	Delays in Evaluating Arthritis Early Questionnaire
7	ECS	Effective Consumer Scale
8	ESS	Epworth Sleepiness Scale
9	FAAM	Foot and Ankle Ability Measure
10	FAOQ	Foot and Ankle Outcome Questionnaire
11	FFF-R	Revised Foot Function Index
12	FIM	Functional Independence Measure
13	FSFI	Female Sexual Function Index
14	GCO	General Coping Questionnaire
15	GDS	Geriatric Depression Scale
16	GSES	Generalised Self-efficacy Scale
17	heiQ	The Health Education Impact Questionnaire
18	HLQ	Health and Labor Questionnaire
19	HPQ	Health and Work Perfomance Questionnaire
20	H5-D	Health State descriptive system
21	IPA	Participation and Autonomy Questionnaire
22	ISI	Insomnia Severity Index
23	LHS	London Handicap Scale
24	LSI	Life satisfaction index
25	LSNS	Lubben Social Network Scale
26	MADRS	Montgomery-Asberg Depression Rating Scale
27	MHIQ	THE McMASTER HEALTH INDEX QUESTIONNAIRE
28	MPQ	McGill Pain Questionnaire
29	OBQ	Occupational Balance Questionnaire
30	PEQ	Patient Experiences Questionnaire
31	PGWBI	Psychological General Well-being Index
32	PHQ-2	Patient Health Questionnaire -2
33	PHQ-9	Patient Health Questionnaire -9
34	PMI	Pain Management Inventory
35	PMS	Profile of Moods State
36	ProF	Profile of Fatigue
37	PROMIS-PI	PROMIS pain interference
38	PSQI	Pittsburg Sleep Quality Index
39	QOLS	Quality of Life Scale (QOLS)
40	SAQ	Sleep Assessment Questionnaire
41	SCL-90-R	Symptom Checklist-90-Revised
42	SIP	Sickness Impact Profile
43	SOC	Sense of Coherence Scale
44	SPADI	Shoulder Pain and Disability Index
45	SQ-RA	Satisfaction Questionnaire for RA
46	XSMFA	Extra Short Musculoskeletal Function Assessment Questionnaire
47	ZDS	Zung Self-rating Depression Scale
